# Coproporphyrin I PBPK model in the open systems pharmacology suite enables precise prediction of OATP 1B- mediated drug interactions through calibrated *In vivo* inhibition constants

**DOI:** 10.3389/fphar.2026.1828033

**Published:** 2026-07-28

**Authors:** Tobias Kanacher, Moriah Pellowe, Christina Kovar, Johanna Eriksson, Jing Wu, David Busse, Jose David Gómez-Mantilla, Peter Stopfer, Ibrahim Ince, Fenglei Huang

**Affiliations:** 1 Pharmetheus AB, Uppsala, Sweden; 2 Boehringer Ingelheim Pharma Inc., Ridgefield, CT, United States; 3 Boehringer Ingelheim Pharma GmbH & Co. KG, Biberach, Germany

**Keywords:** biomarkers, coproporphyrins (CP-I), drug-drug interactions (DDI), OATP1B, Open Systems Pharmacology (OSP), PBPK, statins

## Abstract

**Background:**

Organic Anion Transporting Polypeptide (OATP) 1B1/3 inhibition can lead to clinically significant drug-drug interactions (DDIs). Coproporphyrin-I (CP-I) is an established endogenous biomarker for assessing the OATP1B inhibitory activity of new chemical entities. However, current physiologically based pharmacokinetic (PBPK) models for CP-I are restricted to proprietary software, limiting widespread adoption and collaboration.

**Methods:**

We developed and qualified an open-source PBPK model for CP-I using the Open Systems Pharmacology (OSP) Suite. The model describes the endogenous synthesis and OATP1B1/MRP2-mediated disposition of CP-I. It was integrated into a DDI network comprising the precipitants rifampicin, probenecid, and cyclosporine A, and the object drugs atorvastatin, pitavastatin, and rosuvastatin. A key methodological feature was the estimation of *in vivo* inhibition constants (K_i,OATP1B_) for precipitants based on clinical CP-I data rather than relying solely on *in vitro* measurements.

**Results:**

The CP-I model successfully captured baseline plasma concentration-time profiles with a Geometric Mean Fold Error (GMFE) of 1.15. In the DDI network analysis, using CP-I-derived *in vivo* K_i,OATP1B_ values for the strong OATP1B inhibitors rifampicin and cyclosporine yielded superior predictive accuracy compared to *in vitro* values, with most predicted DDI ratios for statins falling within the 2-fold acceptance range. Specifically for cyclosporine, the model required an adjustment of the endogenous CP-I synthesis rate to account for baseline differences in the specific study population; incorporating this allowed for the estimation of an *in vivo* K_i,OATP1B_ (4.7 nM) that accurately predicted interactions with pitavastatin and rosuvastatin. Notably, probenecid, a weak OATP1B inhibitor, showed an *in vivo* K_i,OATP1B_ estimate within a similar range of its *in vitro* measured value. Consequently, the scaling factor between *in vitro* and *in vivo* K_i,OATP1B_ appears to be a compound-specific parameter requiring independent identification for each precipitant.

**Conclusion:**

This study presents a qualified, freely available PBPK framework for CP-I. It demonstrates that calibrating participant models using early clinical biomarker data enables reliable prospective prediction of OATP1B-mediated DDIs. This workflow supports model-informed drug development (MIDD) strategies, potentially reducing the need for dedicated clinical DDI studies.

## 1 Introduction

Organic Anion Transporting Polypeptides (OATP) 1B1 and 1B3 are multi-specific hepatic uptake transporters that mediate the clearance of many drugs, such as bosentan, fexofenadine, paclitaxel and 3-hydroxy-3-methyl-glutaryl-coenzyme A **(**HMG-CoA) inhibitors (statins), as well as endogenous compounds from the bloodstream (Kullak-Ublick et al., 2001; Maeda, 2015; Huang et al., 2017; Galetin et al., 2024; Bories et al., 2025). Inhibition of OATP1B1/3 activity can lead to significant drug-drug interactions (DDIs), which can result in serious adverse events such as myopathy, rhabdomyolysis, elevated liver enzymes, and acute kidney injury (Balasubramanian and Maideen, 2021). Accurately assessing the potential for OATP1B-mediated DDIs is a critical step in drug development, with regulatory agencies like the U.S. FDA identifying key objects (e.g., rosuvastatin, pitavastatin, atorvastatin) and precipitants (e.g., rifampicin, cyclosporine A) to guide these investigations (FDA, 2025).

Coproporphyrin-I (CP-I), a byproduct of heme synthesis, is a sensitive and established endogenous biomarker for clinically relevant OATP1B inhibition (Lai, 2023; Galetin et al., 2024; FDA, 2025). Recognizing its clinical relevance, the International Transporter Consortium (ITC) recently designated CP-I as a Tier 1 biomarker (Galetin et al., 2024). The suitability of CP-I for this application stems from its disposition pathway, which relies almost exclusively on OATP1B-mediated hepatic uptake for its clearance. This dependency makes plasma CP-I concentrations a direct indicator of OATP1B activity, highly sensitive to pharmacological inhibition.

To translate changes in CP-I concentrations into quantitative DDI predictions, physiologically based pharmacokinetic (PBPK) modeling is an valuable tool. However, despite the biomarker’s importance, the application of CP-I modeling has been constrained by the limited accessibility of existing models, which are exclusively implemented in proprietary commercial software, such as Napp version 2.31 (Yoshikado et al., 2018; Mochizuki et al., 2022) or restricted commercial PBPK software (Kimoto et al., 2022). This lack of a publicly available, transparent model hinders broader academic and industry collaboration in model-informed drug development (MIDD).

Recognizing this limitation, our primary objective was to develop, qualify, and publicly release the first comprehensive PBPK CP-I model within the open-source Open Systems Pharmacology (OSP) Suite (PK-Sim® and MoBi®). Additionally, model’s utility was demonstrated by incorporating it into a DDI network alongside established models of OATP1B objects (atorvastatin, pitavastatin, rosuvastatin) and precipitants (probenecid, rifampicin and cyclosporine A). The performance of this network was evaluated using clinical DDI data to establish a qualified, freely accessible tool to better ensure patient safety by mitigating the risk of OATP-mediated DDIs for new chemical entities.

## 2 Materials and methods

### 2.1 Modeling strategy

The overall workflow for the development, evaluation, and qualification of the PBPK models is illustrated in Supplementary Figure S1. The strategy consisted of three consecutive phases: (1) Model development of the PBPK CP-I model, (2) model evaluation using different K_i,OATP1B_, and (3) qualification of the PBPK-DDI network.

### 2.2 Software

PBPK modeling was performed using PK-Sim® (model development in version 11.2, and final models shared in version 12) and MoBi® modeling software**,** which are components of the freely accessible and open-source OSP Suite. The source code is publicly available on GitHub (Open Systems Pharmacology, 2025), providing access to published models and tutorials. Plots were generated using R version 4.2.2 (R Core Team, 2025) running within RStudio version 2022.12.0.353 (Posit Software, PBC). Pharmacokinetic parameters were generated from simulated and observed data using the R package ncappc (Version 0.3.0).

Parameter optimizations were conducted using the Levenberg-Marquardt and Monte-Carlo algorithms within the OSP Suite software. Subsequently, a sensitivity analysis was conducted in PK-Sim® to evaluate the impact of key or uncertain input parameters, such as the *in vivo* inhibition constant (K_i,OATP1B_), on simulation outcomes.

### 2.3 PBPK model development

#### 2.3.1 CP-I model development

A whole-body PBPK model for CP-I was developed based on available literature on CP-I in human plasma, both in the absence and presence of OATP precipitants rifampicin and probenecid (Supplementary Figure S1). The model was built and calibrated using published clinical data on CP-I plasma concentrations in healthy subjects, at baseline and following co-administration of the OATP inhibitors rifampicin and probenecid. The study designs, dosing regimens, and subject demographics for the clinical data used for model development are detailed in Supplementary Table S1.

The general approach to PBPK model construction follows methods previously detailed by Kuepfer et al. (2016). Relevant adult anthropometric (height, weight) and physiological parameters (e.g., blood flows, organ volumes, binding protein concentrations, hematocrit, cardiac output) were obtained from published literature (Willmann et al., 2007) and implemented as default values in PK-Sim® for adult simulations. The activity and variability of plasma proteins and active processes integrated into PK-Sim® are documented in the publicly available PK-Sim® Ontogeny Database Version 7.3, or referenced individually when specific to a different process.

A population-average PBPK model was developed for CP-I and calibrated using mean plasma concentration data from clinical studies that reported CP-I levels at baseline and following administration of OATP1B inhibitors (Lai et al., 2016; Kunze et al., 2018; Shen et al., 2018; Takehara et al., 2018; Zhang et al., 2020). Simulations were performed using representative individuals whose characteristics were based on the average demographic data from each study. In the absence of specific demographic information, a default profile representing a healthy 30-year-old, 73 kg, 176 cm European male was assumed. As an approximation, the model incorporated tissue-specific expression levels for OATP1B1 and MRP2, the key transporters responsible for CP-I disposition (Meyer et al., 2012). Finally, the endogenous synthesis of CP-I was simulated as a continuous zero-order infusion into liver at a rate of 1.09 mg/kg over 1 year using an estimated value from Yoshikaido et al. (2022). The physicochemical properties of CP-I, obtained through a literature search and used as input parameters for model development, are summarized in Table 1.

**TABLE 1:** Parameter estimates of the CP-I model (basic PK and physicochemical parameters for CP-I.

Model Parameter (Unit)	Value	Source	References
MW (g/mol)	654.71	Literature	Yoshikado et al. (2018)
pKa acid	3.56	Literature	Yoshikado et al. (2018)
pKa base	5.18	Literature	Yoshikado et al. (2018)
Fraction unbound, fu (%)	0.66	Literature	Yoshikado et al. (2018)
Lipophilicity (log units)	2.53	Literature	Yoshikado et al. (2018)
Solubility at pH = 7.0 (mg/mL)	0.03	Literature	DrugBank
GFR fraction	1	assumed	PK-Sim parameter for fraction renal excretion
OATP1B1/3 km (µmol/L)	0.13	Literature	Bednarczyk and Boiselle (2016)
OATP1B1/3 kcat (1/min)	6.6	Optimized	-
MRP2 Km (µmol/L)	7.7	Literature	Gilibili et al. (2017)
MRP2 kcat (1/min)	6.0	Optimized-	​
CP-I synthesis rate in liver intercellular used in MoBi (nmol/h/kg)	0.19	Literature	Yoshikado et al. (2022)
Infusion rate into the liver intracellular used in PK-sim (nmol/h/kg)	0.19	Literature	Yoshikado et al. (2022)

CP-I, coproporphyrin I; fu, fraction unbound in plasma; Lipophilicity, partition coefficient between octanol and water; MRP2, multidrug resistance-associated protein 2; MW, molecular weight; OATP1B, Organic Anion Transporting Polypeptides 1B; pKa, acid dissociation constant of conjugate acid or base.

#### 2.3.2 Model parameters and assumptions for the CP-I model

The PBPK model for CP-I was developed to describe its reabsorption, distribution, metabolism, and elimination by integrating standard human anatomical and physiological parameters from the PK-Sim® database with compound-specific processes, including endogenous synthesis and transporter-mediated hepatic disposition. Physicochemical and kinetic parameters were primarily sourced from literature; however, parameters that were not available or were inadequate to describe the observed concentration-time profiles were fitted (optimized) using the parameter identification module in PK-Sim® (Table 1).

##### 2.3.2.1 Absorption

To exclude intestinal reabsorption, the specific intestinal permeability parameter for CP-I was set to 0. This decision was supported by two observations: (1) initial attempts at parameter identification yielded unrealistically low values, and (2) fixing the parameter to a calculated value led to unreasonable fits, with the fraction excreted in feces conflicting with literature data (Table 1). CP-I solubility was sourced from DrugBank (Table 1).

##### 2.3.2.2 Distribution

CP-I is highly bound to plasma proteins (>99%), with albumin serving as its principal binding partner (Table 1). Consequently, a fraction unbound (plasma, reference value) of 0.66% was applied in the PBPK model. Lipophilicity, characterized by a logP value of 2.53 (Table 1), was considered an important determinant of the volume of distribution. The Rodgers and Rowland model (Rodgers and Rowland, 2006) was selected for predicting tissue:plasma partition coefficients, as it provided a superior fit to observed data among the five methods available in PK-Sim®.

##### 2.3.2.3 Metabolism and elimination

The model included two hepatic transport proteins, OATP1B1 and MRP2, using Michaelis-Menten kinetics. For both transporters, tissue specific mRNA expression based on the inbuild RT-PCR gene expression data (Nishimura et al., 2003) was used as a surrogate for protein abundance (Meyer et al., 2012). Each transporter’s activity was characterized as a saturable Michaelis-Menten process, where K_m_ values were sourced from literature, and k_cat_ values were optimized using clinical data. Additionally, renal elimination was assumed to occur solely via (passive) glomerular filtration, scaled by the unbound fraction. This was implemented by setting the glomerular filtration rate (GFR) fraction parameter to 1, a setting which produced a simulated renal clearance consistent with literature-observed values.

##### 2.3.2.4 CP-I synthesis

Initial model development was conducted in MoBi® to capture the synthesis rate as an explicit reaction within the liver intracellular compartment, ensuring accurate mechanistic reflection. While MoBi® offers high flexibility for defining such custom reactions, PK-Sim® allows for more straightforward linking of participant and substrate models. Therefore, the model was transferred to PK-Sim® to facilitate these integrations. During this transfer, the endogenous CP-I synthesis rate from Mobi® was replaced by a synthesis rate implemented as a continuous zero-order infusion. This infusion, informed by the work of Yoshikado et al. (2022), was directed into the liver’s intracellular space over a 1-year period. This approach was verified to produce simulated results equivalent to those generated by the MoBi® model.

##### 2.3.2.5 Parameter identification and sensitivity analysis

The model development process included parameter identification, i.e., the optimization of certain key parameter values to refine model performance. To reduce manual involvement, the “Parameter Identification” module in PK-Sim® was used. This functionality minimizes the residuals between observed data and simulation outputs by varying selected input parameters within a predefined range. To ensure results were identifiable and reproducible, multiple optimizations with randomized initial values were applied, predominantly using the Levenberg-Marquardt algorithm or Monte-Carlo simulations.

Subsequently, a sensitivity analysis was conducted to assess model reliability and identify key drivers of variability. This analysis, also performed in PK-Sim®, evaluated the impact of key or uncertain input parameters, such as the *in vivo* inhibition constant (K_i_), on simulation outcomes (e.g., AUC_last_ or C_max_ and curve shape).

#### 2.3.3 Precipitant and object drug models

The PBPK models for the OATP1B object drugs (atorvastatin, pitavastatin, rosuvastatin) and precipitants (rifampicin, probenecid, cyclosporine A) were obtained from the OSP community repository on GitHub (Open Systems Pharmacology, 2025). These models have been previously qualified, and their detailed parameterization is documented in the respective model qualification reports available in the repository.

For the current study, the predictive performance of each model was verified to ensure suitability for the investigated DDI scenarios The full qualification of these models, including goodness-of-fit plots and comparisons of simulated versus observed plasma concentration-time profiles, is available in their respective reports in the OSP repository. The specific model versions and repository links are listed in Supplementary Table S2.

### 2.4 Evaluation of PBPK CP-I model

Following successful parameter identification, the predictive performance of the PBPK CP-I model was evaluated both quantitatively and through visual inspection of goodness-of-fit plots (Supplementary Figure S1). Model accuracy was assessed by calculating the Geometric Mean Fold Error (GMFE) (Equation 1), which quantifies the average fold-error between observed and simulated PK parameters and observed plasma concentrations.

$$GMFE=10\frac{\sum \left|\log\left(\frac{Pred}{Obs}\right)\right|}{n}$$ (1)

where both Pred and Obs refer to analyzed PK parameter and nis the number of studies. A GMFE value between 1 and 2.0 (2-fold) was considered indicative of an acceptable model performance.

Goodness-of-fit was visually diagnosed by plotting simulated versus observed plasma concentrations, and inspecting the distribution of data points around the line of unity for systematic bias or trends. As the model represents a mean physiological state with a single, constant baseline concentration for the endogenous object, it does not account for inter-individual variability in baseline concentrations. Consequently, a vertical spread of observed data points corresponding to the single simulated baseline concentration was anticipated.

To verify the performance of the baseline model, the simulated steady-state plasma concentration-time profile of CP-I was compared with clinically observed baseline CP-I PK profiles in healthy subjects. The specific clinical studies used for this comparison are summarized in Supplementary Table S1. Model performance was assessed by visually overlaying the simulated concentration-time profile with the reported mean and variability (if available) plasma concentrations from each clinical study.

### 2.5 DDI model evaluation: Inhibitor parameterization and sensitivity

To simulate DDIs, *in vivo* inhibition constants (K_i,OATP1B_) were determined for the OATP1B precipitants rifampicin and probenecid.

The *in vivo* K_i,OATP1B_ for rifampicin and probenecid was determined by fitting the verified PBPK CP-I model to data from 6 clinical DDI studies (Lai et al., 2016; Kunze et al., 2018; Shen et al., 2018; Takehara et al., 2018; Mori et al., 2020; Zhang et al., 2020). The parameterization involved an initial manual visual sensitivity assessment to define a reasonable parameter space, after which the final value was optimized using the automated “Parameter Identification” module (Levenberg-Marquardt algorithm). The objective of this parameter identification was the minimization of residuals between the simulated concentration-time profiles and the observed clinical data (with and without the precipitant). A similar process was used to define the K_i,OATP1B_ for probenecid, using clinical data from Zhang et al. (2020).

The resulting *in vivo* K_i_ values estimated by the model were then compared with published values (Bednarczyk and Boiselle, 2016; Kimoto et al., 2022; Mochizuki et al., 2022) to ensure physiological plausibility.

Subsequently, a local sensitivity analysis was conducted to evaluate the impact of the newly estimated rifampicin K_i,OATP1B_ on DDI simulation outcomes. This analysis followed the approach described by Kimoto et al. (2022), systematically varying the K_i_ value and observing the corresponding change in the predicted AUC_last_ ratio.

### 2.6 Qualification of the DDI model network

**FIGURE 1:**
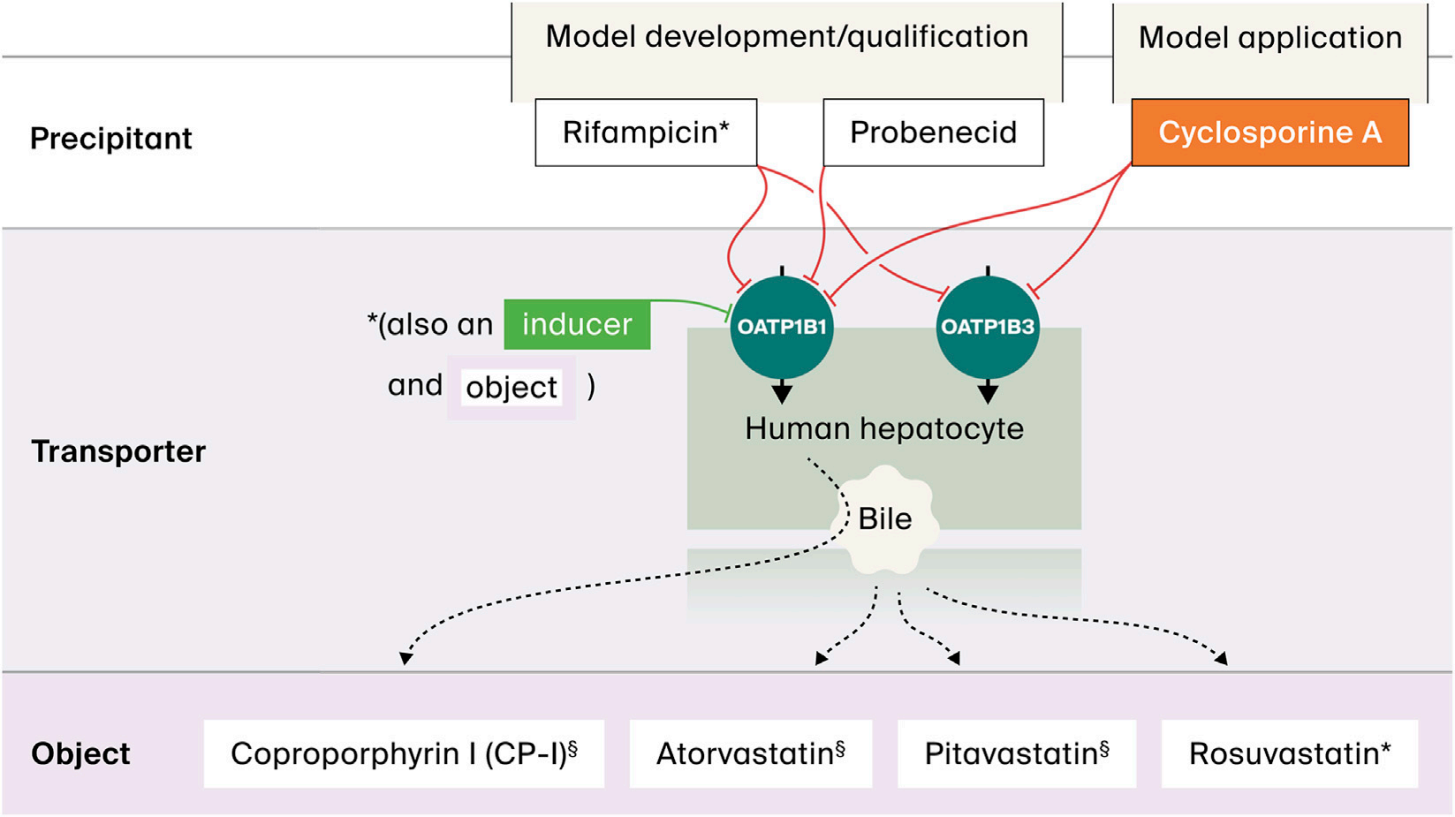
Schematic of OATP1B1- and OATP1B3-mediated DDIs network in human hepatocytes. The hepatic uptake transporters OATP1B1 and OATP1B3 facilitate the clearance of objects, including statins (atorvastatin, pitavastatin, rosuvastatin) and the endogenous biomarker CP-I, into the bile. Precipitant drugs, such as probenecid and rifampicin, can inhibit the function of these transporters (indicated by red lines). Rifampicin also exhibits a complex role as an inducer of OATP1B1 (indicated by the green arrow). CP-I, Coproporphyrin I; DDI, Drug-drug interaction; OATP1B, Organic Anion Transporting Polypeptides 1B.

To qualify the model for predicting OATP1B1/3-mediated DDIs, a PBPK modeling network was established using previously verified PBPK models for selected OATP1B1/3 precipitants and object drugs (Figure 1). The PBPK models for the object drugs atorvastatin, pitavastatin, and rosuvastatin were sourced directly from the OSP Suite community repository (see Supplementary Table S2) and used as published.

The atorvastatin model is designed to serve as an object drug for CYP3A4-and OATP1B1/1B3-mediated DDIs. It has been developed and validated using data from 25 clinical studies, covering a wide dosing range (1.0–80.0 mg) across both single and multiple oral doses.

The pitavastatin model is designed to serve as an object drug for OATP1B1-mediated DDIs. It has been developed and validated using data from 6 clinical studies, covering a range of 0.2–4 mg including intravenous and oral administration.

The rosuvastatin model is designed to serve as an object drug for OATP1B1/1B3-mediated DDIs. It has been developed and validated using data from 42 clinical studies covering a wide dosing range (0.002–80.0 mg) including both intravenous and oral administration.

The newly developed and verified CP-I model was also integrated into the network.

Network qualification was performed by simulating a series of clinical DDI scenarios (Supplementary Figure S1). First, the *in vivo* K_i,OATP1B_ for rifampicin and probenecid were estimated by fitting the verified baseline CP-I model to their respective clinical DDI data. These estimated *in vivo* K_i,OATP1B_ values were then implemented into the PBPK models for rifampicin and probenecid.

Finally, the predictive performance of the complete DDI network was qualified by simulating the following interactions and comparing the predicted DDI effect (AUC_last_ and C_max_ ratios) with reported clinical outcomes: (1) Rifampicin with rosuvastatin, atorvastatin, pitavastatin, and CP-I; (2) Probenecid with rosuvastatin and CP-I. A summary of these DDI studies is provided in Supplementary Table S3, and an overview of the doses and population for each study is available in Supplementary Table S4. The simulated plasma concentration-time profiles were subjected to non-compartmental analysis (NCA) to derive pharmacokinetic parameters, including the AUC_last_ and the C_max_.

The specific dosing regimens for each simulation and the corresponding clinical studies used for verification are summarized in Table 2.

**TABLE 2:** Summary table for OATP1B1/3 DDI with predicted *versus* observed AUC_last_ and C_max_ ratios of CP-I, atorvastatin, rosuvastatin, and pitavastatin, after administration of precipitant.

Precipitant	Object	AUC_last_ ratio			C_max_ ratio			References
		Predicted	Observed	Pred/Obs	Predicted	Observed	Pred/Obs	
Rifampicin, 600 mg, p.o	Coproporphyrin I, p.o	2.80	3.89	0.72	4.39	5.10	0.86	Lai et al. (2016)
Rifampicin, 600 mg, p.o	Coproporphyrin I, p.o	3.28	3.08	1.07	4.39	4.57	0.96	Kunze et al. (2018)
Rifampicin, 150 mg, p.o., SD	Coproporphyrin I, p.o	1.57	1.60	0.98	2.50	2.66	0.94	Mori et al. (2020)
Rifampicin, 300 mg, p.o., SD	Coproporphyrin I, p.o	2.04	2.32	0.88	3.45	3.29	1.05	Mori et al. (2020)
Rifampicin, 600 mg, p.o., SD	Coproporphyrin I, p.o	2.90	3.55	0.82	4.39	5.08	0.86	Mori et al. (2020)
Rifampicin, 600 mg, p.o	Coproporphyrin I, p.o	2.71	3.22	0.84	4.05	5.14	0.79	Shen et al. (2018)
Probenecid, 1,000 mg, p.o	Coproporphyrin I, p.o	1.36	1.44	0.94	1.63	1.46	1.12	Zhang et al. (2020)
Rifampicin, 300 mg, p.o., SD	Coproporphyrin I, p.o	2.06	2.20	0.94	3.45	3.09	1.11	Takehara et al. (2018)
Rifampicin, 600 mg, p.o., SD	Coproporphyrin I, p.o	2.90	3.43	0.84	4.39	4.56	0.96	Takehara et al. (2018)
Rifampicin, 300 mg, p.o., SD	Pitavastatin, p.o	5.48	2.40	2.29	3.83	3.16	1.21	Takehara et al. (2018)
Rifampicin, 600 mg, p.o., SD	Pitavastatin, p.o	10.29	2.88	3.58	5.11	2.98	1.72	Takehara et al. (2018)
Rifampicin, 150 mg, p.o., SD	Pitavastatin, p.o	3.15	3.58	0.88	2.61	3.24	0.80	Mori et al. (2020)
Rifampicin, 300 mg, p.o., SD	Pitavastatin, p.o	5.74	4.63	1.24	3.83	4.04	0.95	Mori et al. (2020)
Rifampicin, 600 mg, p.o., SD	Pitavastatin, p.o	10.71	5.41	1.98	5.11	3.77	1.35	Mori et al. (2020)
Rifampicin, 600 mg, p.o	Pitavastatin, p.o	10.33	5.73	1.80	4.11	7.20	0.57	Chen et al. (2013)
Rifampicin, 600 mg, p.o	Pitavastatin, p.o	8.86	4.68	1.89	3.71	3.94	0.94	Prueksaritanont et al. (2014)
Rifampicin, 600 mg, i.v., p.o., SD	Pitavastatin, p.o	9.11	5.81	1.57	3.99	5.47	0.73	Prueksaritanont et al. (2014)
Rifampicin, 600 mg, i.v., p.o	Rosuvastatin, p.o	4.85	3.68	1.32	12.40	5.94	2.09	Prueksaritanont et al. (2014)
Rifampicin, 600 mg, p.o., SD	Rosuvastatin, p.o	5.42	4.82	1.12	13.85	8.88	1.56	Prueksaritanont et al. (2014)
Rifampicin, 600 mg, p.o., SD	Rosuvastatin, p.o	5.85	5.85	1.00	16.87	12.41	1.36	Prueksaritanont et al. (2017)
Rifampicin, 600 mg, i.v., p.o	Rosuvastatin, p.o	4.39	3.97	1.11	13.34	7.94	1.68	Wu et al. (2017)
Rifampicin, 600 mg, p.o., SD	Rosuvastatin, p.o	4.91	3.75	1.31	14.60	9.96	1.46	Wiebe et al. (2020)
Probenecid, 1,000 mg, p.o., SD	Rosuvastatin, p.o	2.36	2.63	10.90	3.3	4.55	0.73	Wiebe et al. (2020)
Rifampicin, 600 mg, p.o., MD	Atorvastatin, p.o	0.70	0.26	2.66	1.20	0.60	1.99	Backman et al. (2005)
Rifampicin, 600 mg, i.v., SD	Atorvastatin, p.o	9.37	7.89	1.19	16.74	10.83	1.55	Lau et al. (2007)
Rifampicin, 150 mg, p.o., SD	Atorvastatin, p.o	2.47	3.40	0.73	3.98	7.22	0.55	Mori et al. (2020)
Rifampicin, 300 mg, p.o., SD	Atorvastatin, p.o	4.45	5.25	0.85	7.51	11.10	0.68	Mori et al. (2020)
Rifampicin, 600 mg, p.o., SD	Atorvastatin, p.o	8.84	7.39	1.20	14.27	13.06	1.09	Mori et al. (2020)
Rifampicin, 300 mg, p.o., SD	Atorvastatin, p.o	4.45	3.53	1.26	7.51	9.72	0.77	Takehara et al. (2018)
Rifampicin, 600 mg, p.o., SD	Atorvastatin, p.o	8.84	4.85	1.82	14.27	13.27	1.08	Takehara et al. (2018)

AUC_last_, area under the plasma concentration-time curve from time zero to the last measurable concentration; C_max_, maximum plasma concentration; CP-I, coproporphyrin I; i. v., intravenous; MD, multiple doses; Obs, observed; OATP1B1/B3, Organic Anion Transporting Polypeptide (OATP) 1B1/3; PROB, probenecid; Pred, predicted; p. o., oral administration; RIF, rifampicin; SD, single dose.

### 2.7 Application of the DDI network

Following the qualification of the PBPK-DDI network, the framework was extended to include cyclosporine A as a precipitant, using the model sourced from the OSP repository (see Supplementary Table S2). The strategy for this model application is illustrated in Supplementary Figure S2. The analysis followed a sequential, three-step process using clinical data from Mochizuki et al. (2022). First, the baseline CP-I concentrations in the study population (n = 10, Japanese subjects) were compared to the model’s development dataset. As the baseline levels differed, the endogenous CP-I synthesis rate was re-optimized by fitting the model to the mean baseline (control) CP-I plasma concentrations from this specific study. Second, using this population-adjusted baseline model, the *in vivo* K_i,OATP1B_ for cyclosporine A was estimated. This was achieved by fitting the model to the CP-I plasma concentration-time profiles observed during co-administration with different cyclosporine A doses and regimens. The resulting *in vivo* K_i,OATP1B_ was then compared to published *in vitro* values (Kimoto et al., 2022). Additionally, the *in vivo*/*in vitro* K_i,OATP1B_ ratio was evaluated against previously derived ratios for rifampicin and probenecid.

And the ratio of the *in vivo/in vitro* K_i,OATP1B_ was compared to previously derived ratios for rifampicin and probenecid. Finally, to qualify the predictive performance of this newly estimated parameter, the cyclosporine A *in vivo* K_i,OATP1B_ was implemented. The DDI network was then used to prospectively simulate the DDI effect of cyclosporine A on the object drugs pitavastatin and rosuvastatin. The predicted AUC_last_ and C_max_ interaction ratios were evaluated against observed clinical data.

## 3 Results

### 3.1 Development and evaluation of the baseline PBPK CP-I model

The final parameters for the developed PBPK for CP-I are summarized in Table 1.

The PBPK model successfully captured the observed clinical plasma concentration-time profiles of CP-I. The model’s predictive accuracy was high, with a GMFE of 1.15, falling well within the predefined acceptance range of 1–2.0. A goodness-of-fit plot demonstrated a strong correlation between simulated and observed concentrations, with data points distributed symmetrically around the line of unity, indicating a lack of significant systematic bias (Supplementary Figure S3).

As anticipated by the model’s design, a horizontal spread of observed data points was evident at the baseline CP-I concentration, representing the inter-individual variability observed in clinical subjects relative to the single simulated baseline from the ‘mean state’ model (Supplementary Figure S3). The simulated steady-state CP-I concentration of 0.92 nM was in agreement with the mean baseline concentrations reported across multiple clinical studies (Supplementary Figure S3). This confirmed the model provided a reliable framework for predicting DDIs.

### 3.2 Estimation of *in vivo* K_i,OATP1B_ and assessment of predictive performance

**FIGURE 2:**
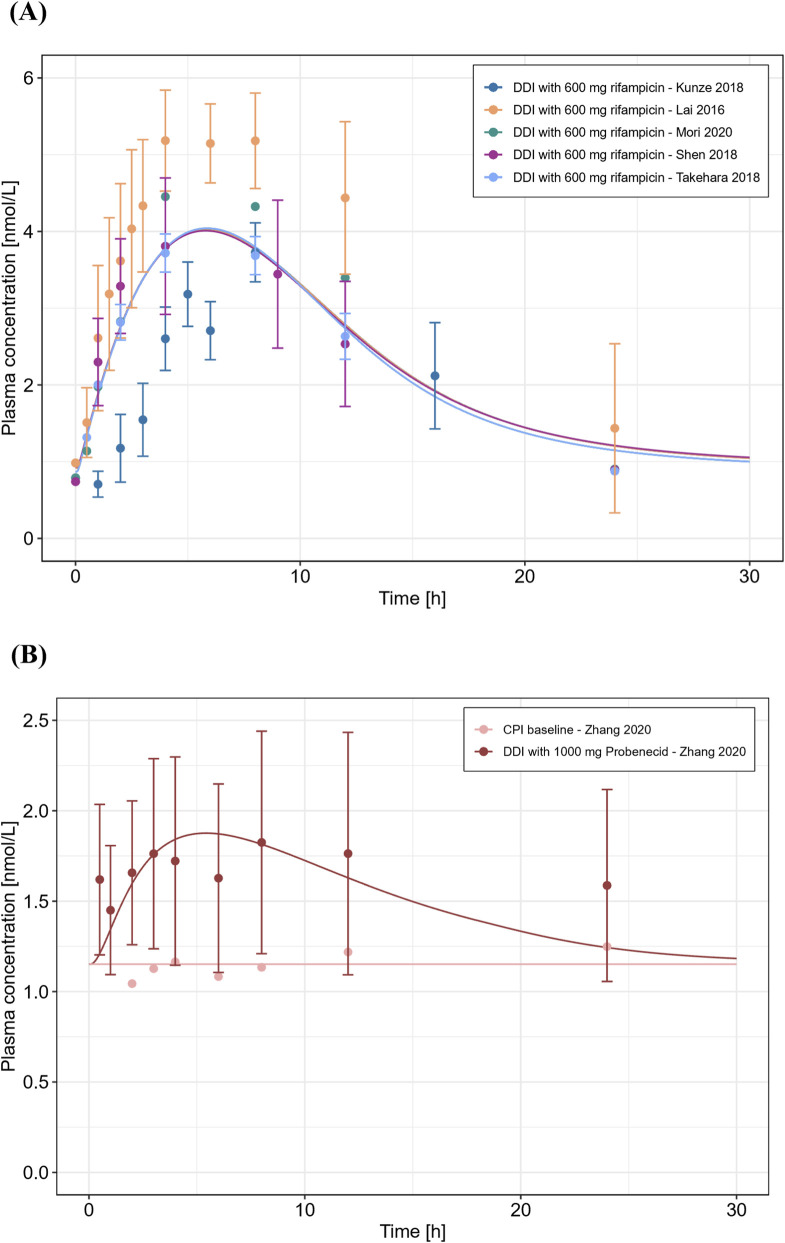
Simulated CP-I levels at steady state, without and with single doses of 600 mg rifampicin **(A)** or 1,000 mg probenecid **(B)**. The solid lines represent the PBPK model simulations for a typical individual. Note that in **(A)**, the purple (Shen et al., 2018) and light blue (Takehara et al., 2018) lines are distinguishable due to varying biometrics, while other profiles are overlaid because their biometric parameters are nearly equivalent. The circles represent observed clinical data taken from the literature: dark blue (Kunze et al., 2018), orange (Lai et al., 2016), green (Mori et al., 2020), purple (Shen et al., 2018), and light blue (Takehara et al., 2018). Data are presented as mean ± SD. CP-I, Coproporphyrin I; DDI, Drug-drug interaction; PBPK, physiologically based pharmacokinetic; SD, standard deviation.

The verified baseline model was applied to clinical DDI studies involving the OATP1B inhibitors rifampicin and probenecid. The model accurately reproduced the observed CP-I plasma concentration-time profiles both at baseline (Supplementary Figure S3) and following co-administration of rifampicin (Figure 2A; Supplementary Figures S4A–D) and probenecid (Figure 2B).

By performing a simultaneous fit—weighted by sample size—of the clinical data from Lai et al. (2016), Kunze et al. (2018), Shen et al. (2018), Takehara et al. (2018), the *in vivo* K_i,OATP1B_ for rifampicin was estimated at 0.17 µM (95% CI: 0.12–0.22 µM) based on CP-I plasma levels (Supplementary Table S6). This is about 2-fold more potent than the reported *in vitro* values (0.29 µM (Kimoto et al., 2022) and 0.47 [Hirano 2006]). For probenecid the model was fitted the data from Zhang et al. (2020). Notably the Baseline data from this study is at the upper edge compared to the other studies (Supplementary Figure S3). Consequently, the endogenous CP-I synthesis rate needed to be fitted to describe the baseline plasma concentrations in this study population (n = 14, Indian subjects). The resulting rate was 25% increased compared to the mean rate used for rifampicin. By incorporating this adjustment, the *in vivo* K_i,OATP1B_ for probenecid was estimated at 35.2 µM (95% CI: 23.9–46.5 µM), which aligns closely with the reported *in vitro* value of 39.8 µM (Izumi et al., 2016) and within the confidence interval.

**FIGURE 3:**
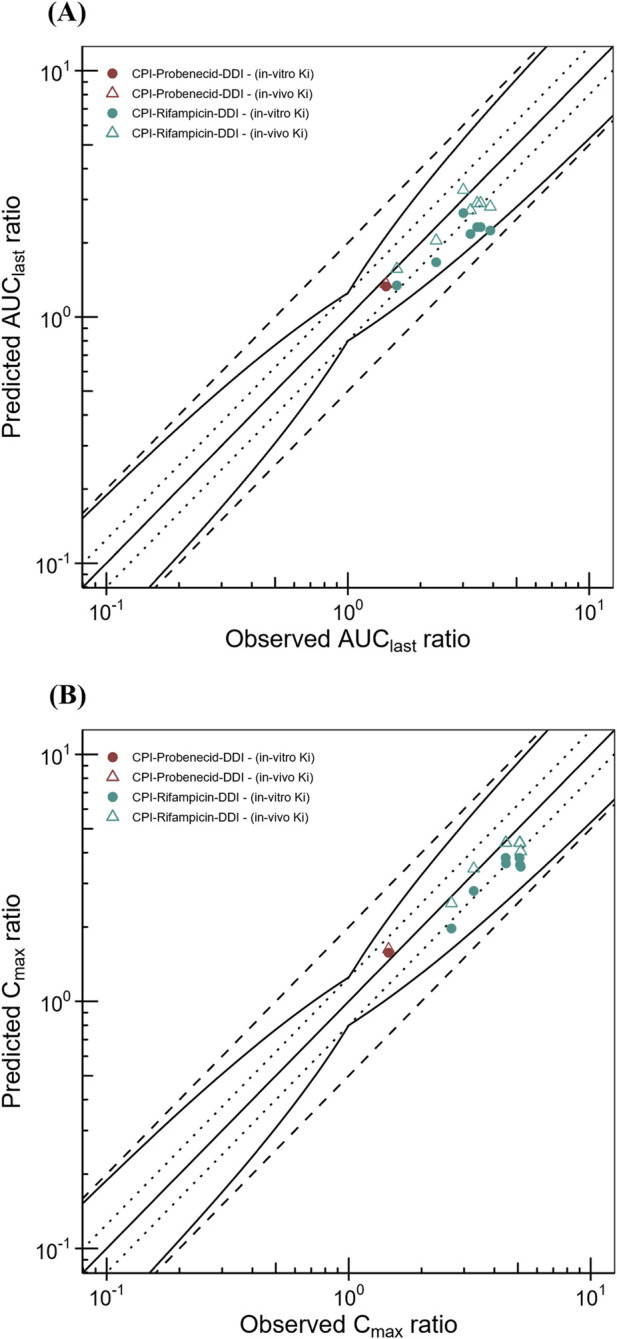
Sensitivity analysis of simulated DDI ratios for CP-I using *in vitro* versus *in vivo* optimized K_i,OATP1B_. Panels show the predicted versus observed DDI ratios of **(A)** AUC_last_ and **(B)** C_max_. Red symbols represent the DDI with probenecid, and green symbols represent the DDI with rifampicin. Circles represent predictions using *in vitro* K_i_ values, while triangles represent predictions using *in vivo* optimized K_i_ values. The solid straight line represents the line of identity, while the dotted and dashed lines indicate the 1.25-fold and 2-fold deviation. Curved lines represent the limits of the predictive measure proposed by Guest et al. (2011) with 1.25-fold variability. AUC_last_, area under the plasma concentration-time curve from time zero to the last measurable concentration; C_max_, maximum plasma concentration; CP-I, Coproporphyrin I; DDI, Drug-drug interaction; K_i,OATP1B_, *in vivo* inhibition constant for Organic Anion Transporting Polypeptides 1B; OATP1B, Organic Anion Transporting Polypeptides 1B.

A Sensitivity analysis comparing DDI predictions using the newly calibrated *in vivo* K_i,OATP1B_ *versus* the published *in vitro* value confirmed that the *in vivo* K_i,OATP1B_ for rifampicin provided a better description of the observed clinical data for C_max_, while for probenecid the results look similar (Figure 3). Accordingly, the GMFE for the OATP1B1/3 DDI ratio using was improved from 1.49 to 1.38 (rifampicin and probenecid) for C_max_, using the *in vivo* K_i,OATP1B_, while it was similar for AUC_last_ (1.36 with *in vivo* K_i,OATP1B_ and 1.38 with *in vitro* K_i,OATP1B_).

**FIGURE 4:**
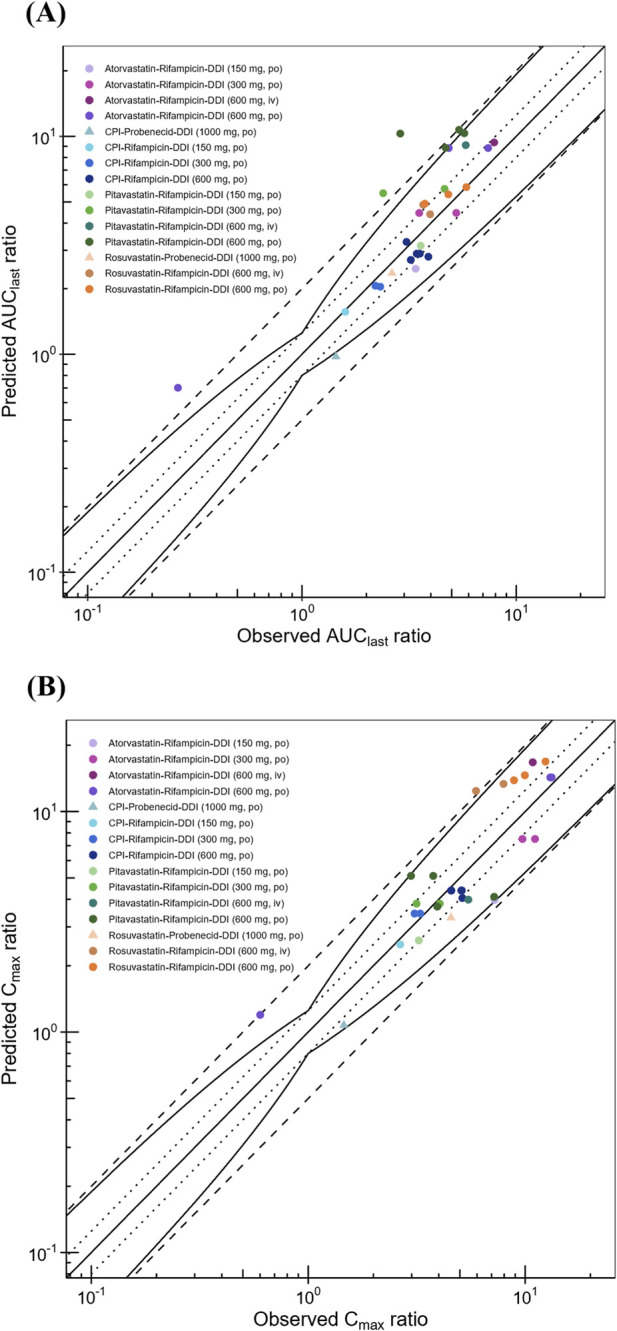
Predicted versus observed DDI **(A)** AUC_last_ and **(B)** C_max_ ratios. The solid black line represents the line of identity, while the dotted and dashed lines indicate the 1.25-fold and two-fold deviation. Curved lines represent the limits of the predictive measure proposed by Guest et al. (2011) with 1.25-fold variability. Each color represents one combination of drugs. Study references and values of predicted and observed DDI AUC_last_ ratios and DDI C_max_ ratios are listed in Table 2. CP-I, Coproporphyrin I; DDI, Drug-drug interaction; iv, intravenous; po, oral administration.

Additionally, using the calibrated *in vivo* K_i,OATP1B_ yielded predictions for the statins that were highly consistent with the observed clinical data (Figures 2A,B; Figures 4A,B).

### 3.3 PBPK models of additional objects and their validation

To construct the complete DDI network, PBPK models for the additional object and precipitant drugs were either newly developed or sourced from the OSP community repository.

PBPK models for atorvastatin and pitavastatin were newly developed for this work. Both models were built using published clinical pharmacokinetic data and mechanistic information on their absorption, distribution, metabolism, and elimination (ADME) pathways, including OATP1B-mediated hepatic uptake. The models were subsequently qualified against a range of clinical studies, demonstrating strong descriptive and predictive performance for plasma concentration-time profiles and key PK parameters. Comprehensive model evaluation reports, detailing the specific input parameters, model assumptions, and goodness-of-fit plots for these newly developed models, are provided in Supplementary Data Sheet S1 (atorvastatin) and Supplementary Data Sheet S2 (pitavastatin).

Previously qualified PBPK models for rifampicin (Hanke et al., 2018), rosuvastatin (Hanke et al., 2021), and probenecid (Britz et al., 2020) were obtained from the OSP model repository (Open Systems Pharmacology, 2025). Full model evaluation reports, which include detailed goodness-of-fit plots and plasma concentration-time profiles, are available in the OSP repository (see Supplementary Table S2).

### 3.4 Predictive performance of the PBPK-DDI model

The predictive performance of all models was verified to ensure suitability for the investigated DDI scenarios. As part of this verification, the predicted versus observed DDI AUC_last_ and C_max_ ratios for the object drugs pitavastatin, rosuvastatin, and atorvastatin are presented in Figures 4A,B.

The PBPK-DDI network was qualified by comparing its predictive performance to clinical data. Overall, the network performed well, with predicted drug exposure ratios AUC_last_ and C_max_ (Table 2) that were generally consistent with observed mean and reported variability. The GMFE for the OATP1B1/3 DDI ratio *in vivo* K_i,OATP1B_ was 1.38 (rifampicin and probenecid) for both AUC_last_ and C_max_, a value well within the predefined acceptance range of one and 2.0.

In addition to the GMFE analysis, the predictive performance was assessed by calculating the proportion of predicted DDI ratios falling within acceptance limits. As shown in Supplementary Table S5, the majority of predictions fell within the standard 2-fold range, with 90.0% of predicted AUC_last_ ratios (27/30) and 96.7% of C_max_ ratios (29/30) within 2-fold of the observed values. Furthermore, when applying the stricter acceptance limits proposed by Guest et al. (2011), which account for 1.25-fold inter-study variability, the model maintained high predictive accuracy: 83.3% of AUC_last_ and 93.3% of C_max_ predictions fell within these boundaries.

For interactions involving rifampicin as the precipitant, the model accurately predicted the DDI effect on CP-I, atorvastatin, and rosuvastatin. Notably, the model successfully captured the substantial increase in CP-I concentrations following rifampicin administration (Supplementary Figure S4), with most AUC_last_ and C_max_ ratios falling within the 2-fold acceptance range of observed mean and reported variability. Some minor discrepancies were observed where predictions exceeded this boundary. This occurred for the AUC_last_ ratio of pitavastatin with 300 mg and 600 mg rifampicin doses (Takehara et al., 2018; Mori et al., 2020), the C_max_ ratio for rosuvastatin in one study (Prueksaritanont et al., 2014), and both the AUC_last_ and C_max_ ratios for atorvastatin in another simulated study (Backman et al., 2005).

For interactions involving probenecid as the precipitant, the model correctly predicted the inhibitory effect on the exposures of both CP-I and rosuvastatin, with all AUC_last_ and C_max_ ratios falling within the 2-fold acceptance range (Table 2). Notably, the predicted increase in baseline CP-I concentrations following probenecid administration aligned well with the clinical observations from Zhang et al. (2020) as shown in Figure 2B.

### 3.5 Application of the PBPK-DDI network to cyclosporine A

The CP-I PBPK model framework was subsequently applied to predict DDIs involving cyclosporine A as the precipitant. As a first step, the *in vivo* K_i,OATP1B_ for cyclosporine A was estimated across different dosing regimens using measured CP-I plasma concentrations from Mochizuki et al. (2022).

**FIGURE 5:**
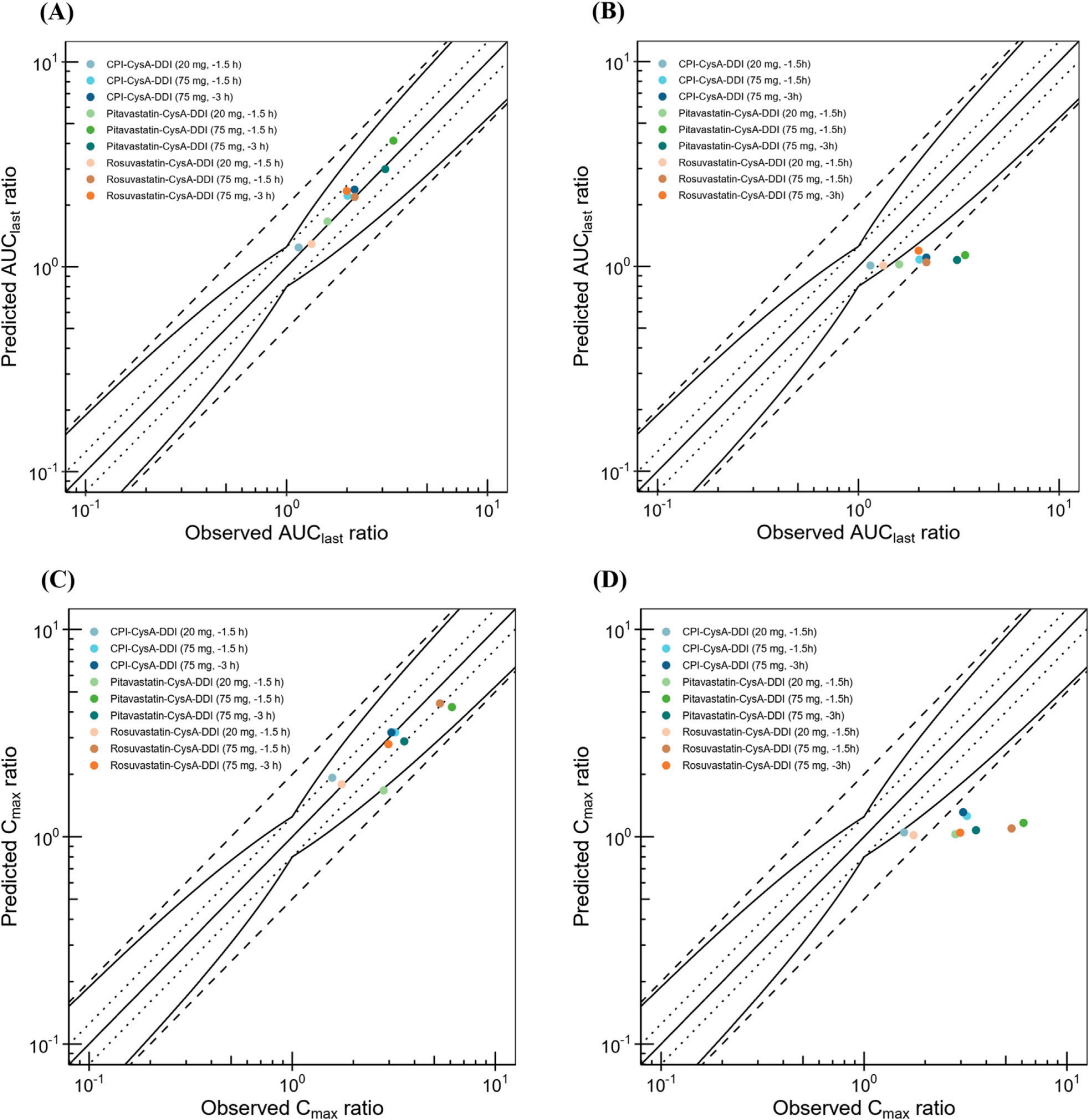
Predicted versus observed DDI using the *in vivo* Ki **(A)** AUC_last_ and **(C)** C_max_ ratios and using the *in vitro* Ki **(B)** AUC_last_ and **(D)** C_max_ ratios for cyclosporine A as precipitant in the application part. The straight black lines represent the lines of identity, while the dotted and dashed lines indicate the 1.25-fold and two-fold deviation. The curved solid black lines represent the limits of the predictive measure proposed by Guest et al. (2011) with 1.25-fold variability. Each color represents one combination of drugs. CP-I, Coproporphyrin I; CysA, Cyclosporine A; DDI, Drug-drug interaction.

Notably, the baseline CP-I concentration without cyclosporine A differed compared to previously used datasets. Consequently, the endogenous CP-I synthesis rate needed to be fitted to describe the baseline plasma concentrations in the study population (n = 10, Japanese subjects). The resulting rate was 0.08 (nmol/h/kg), representing 42% of the original synthesis rate. Incorporating this adjustment, the *in vivo* K_i,OATP1B_ for cyclosporine A as precipitant was estimated to be 4.7 nM. This value represents a 30-fold higher potency than the reported *in vitro* K_i,OATP1B_ for cyclosporine A (Kimoto et al., 2022). Further it is 36- and 7500-fold lower to the *in vivo* K_i,OATP1B_ for rifampicin and probenecid, respectively. Figure 5 compares observed and predicted exposure ratios (AUC_last_ and C_max_), demonstrating the predictive accuracy of the estimated *in vivo* K_i_.

Using the estimated *in vivo* K_i,OATP1B_ for cyclosporine A, the DDI effect of cyclosporine A as precipitant on pitavastatin and rosuvastatin as subjects could be predicted for different doses and regimens of cyclosporine A. The good model predictive performance can be seen in Supplementary Figure S5 (CP-I), Supplementary Figure S6 (pitavastatin), and Supplementary Figure S7 (rosuvastatin).

## 4 Discussion

PBPK modeling, together with mechanistic statistical modeling, has increasingly been recognized as a valuable tool for DDI prediction and the potential waiver of dedicated DDI studies (Gomez_mantilla et al., 2023; Nguyen et al., 2025). However, limitations remain in its application to the prediction of transporter-mediated DDIs (Grimstein et al., 2019). In recent years, endogenous biomarkers have been proposed as tools to assess transporter-mediated DDIs using Phase I data. This strategy allows early identification of transporter involvement without the need for probe drug co-administration. The observed changes in endogenous biomarker levels can be used in conjunction with PBPK modeling to support model-informed predictions of interactions with common substrates. This integrated approach has the potential to reduce the number of dedicated clinical DDI studies, accelerate drug development timelines, and minimize the exposure of healthy volunteers to unapproved investigational drugs (Kimoto et al., 2022; Choi et al., 2024; Rodrigues and Wezalis, 2025).

This study describes the development and qualification of a PBPK model for the endogenous biomarker CP-I. Developed within the open-source OSP Suite, the model is intended as a quantitative tool to de-risk the potential for OATP1B1/3-mediated DDIs (e.g., DDIs with statins) with new chemical entities. The resulting framework successfully reproduced the endogenous baseline concentrations of CP-I and formed the basis for a comprehensive DDI network analysis.

The predictive performance of the model was qualified using a network of clinically reported DDIs. The framework demonstrated strong overall performance, with most simulated DDI AUC_last_ and C_max_ ratios closely aligning with observed clinical data and falling within the predefined 2-fold acceptance boundary. For instance, the inhibitory effects of rifampicin on CP-I, atorvastatin, and rosuvastatin were accurately predicted. Similarly, the model correctly captured the inhibitory effects of probenecid on the disposition of CP-I and rosuvastatin. A notable exception was the overprediction of the DDI between rifampicin and pitavastatin, where simulations slightly exceeded the 2-fold boundary in some scenarios. This deviation, however, is consistent with the high inter-individual and inter-study PK variability reported for this object (Huang et al., 2014).

This high variability in statin disposition is largely driven by genetic polymorphisms in the *SLCO1B1* gene, which encodes the OATP1B1 transporter. A limitation of our developed model is that it does not account for these potential drug-drug-gene interactions. The influence of *SLCO1B1* genotype on both statin pharmacokinetics and basal CP-I levels is an active area of research (Türk et al., 2019; Reig-López et al., 2022; Ujihira et al., 2025) and future work could focus on extending the CP-I PBPK model to account mechanistically for the effects of the most common *SLCO1B1* polymorphisms, for instance by adjusting the k_cat_ of the *SLCO1B1* transporter according to the genotype. In a clinical setting, such a pharmacogenomic model could potentially incorporate an individual’s basal CP-I concentration, which can be influenced by biosynthesis differences, genotype- or potential ethnicity-related system variations, to provide more personalized and accurate DDI predictions for new chemical entities that inhibit OATP1B transporters.

To address this variability in our current model without having details genotyping or ethnicity for all used literature studies at hand, we used a more pragmatic approach: calibrating the baseline CP-I synthesis rate to the specific study population. During model development we adjusted model parameters using a fixed CP-I synthesis rate from literature (Yoshikado et al., 2022) to describe the mean of the three studies (Lai et al., 2016; Takehara et al., 2018; Zhang et al., 2020) used for development with CP-I baseline data. However, applying this fixed synthesis rate to the data of the DDI study with probenecid and cyclosporine A (Zhang et al., 2020; Mochizuki et al., 2022) as precipitants resulted in a poor description of the baseline and biased DDI predictions (not shown). We hypothesized that the mean synthesis rate in the study population of theses DDI studies with probenecid and cyclosporine A might be altered due to different *SLCO1B1* polymorphisms or ethnicity of the included subjects. Fitting the synthesis rate to the mean baseline of the study population resulted in an increased rate of 25% and 42% for probenecid and cyclosporine A, respectively, compared to the one estimated for model development. Notably, the study populations varied significantly; probenecid was investigated in an Indian population (Zhang et al., 2020) and cyclosporine A in a Japanese population (Mochizuki et al., 2022), whereas the models were originally developed using Caucasian or Japanese data. Significant baseline variability was observed even within the Japanese population, likely stemming from genotypic differences. In our simulations, matching biometrics had a negligible impact on the results; as shown in Figure 2A, only the simulations for Shen et al. (2018) and Takehara et al. (2018) are distinguishable, while those with similar biometric profiles are superimposed. Consequently, the visible differences in the observed data likely arise from variations in the synthesis rate. Adjusting this rate to the mean of each specific study population significantly improved the DDI predictions. This approach eliminated previous biases and resulted in close alignment with the measured data (Figures 3, 5; Supplementary Figures S5–S7). These findings suggest that a population-specific baseline fit enhances prediction accuracy, highlighting the necessity of adjusting CP-I baselines when reported levels deviate substantially from the model—especially when the underlying causes, such as genotype or ethnicity effects, remain uncharacterized. This greatly enhances the applicability of the CP-I model for clinical use as it is circumventing the need for detailed genotyping of the study subjects.

A key methodological advancement in this study was the use of precipitant-specific *in vivo* inhibition constants (Kᵢ) derived from clinical DDI data involving the endogenous biomarker CP-I. By anchoring the precipitant’s inhibitory potential to its effect on CP-I, the model could mechanistically and quantitatively predict interactions across multiple OATP1B1/3 objects. This approach highlights the utility of CP-I as a sensitive *in vivo* probe for calibrating precipitant parameters, potentially offering improved predictive accuracy relative to estimates derived from *in vitro* systems. Indeed, the application of calibrated *in vivo* Kᵢ,_OATP1B_ across objects like CP-I, rosuvastatin, pitavastatin, and atorvastatin yielded more accurate DDI predictions (mostly within 2-fold, Table 2; Figure 4) than would be expected with *in vitro* measurements alone.

Finally, the inclusion of probenecid, rifampicin and cyclosporine A as precipitants was a deliberate methodological choice. Although other compounds are known to cause clinical OATP1B inhibition and associated increases in CP-I exposure (e.g., Kikuchi et al., 2024), these three were selected because they offered a coherent clinical dataset, including essential baseline CP-I measurements. Utilizing these baseline values to calibrate the CP-I synthesis rate was a crucial step in estimating precipitant-specific *in vivo Ki* values. Despite being a weaker, non-reference OATP1B inhibitor, probenecid’s relative selectivity for OATP1B1/3 proved valuable for interrogating this specific DDI pathway within the network (Kimoto et al., 2022; Ujihira et al., 2025). Furthermore, our findings suggest that the *in vivo* Kᵢ,_OATP1B_ for probenecid aligns closely with its reported *in vitro* values. In contrast, for rifampicin—a potent but potentially less selective OATP1B1/3 inhibitor—the identified *in vivo* Kᵢ,_OATP1B_ was approximately twofold lower than *in vitro* values, leading to improved DDI predictions for C_max_. For cyclosporine A, the strongest OATP1B1/3 inhibitor with known time-dependent effects *in vitro*, the estimated *in vivo* Kᵢ,_OATP1B_ was approximately sixfold lower than its *in vitro* counterpart. These results strongly underline that the *in vivo* Kᵢ,_OATP1B_ is a compound-specific parameter requiring independent identification for each precipitant. Clearly, more in-depth investigations to investigate, e.g., the impact of fraction unbound or intracellular accumulation with a broader range of precipitants are warranted to shed further light on this highly relevant topic.

Future investigations will benefit significantly from the implementation of this platform within the open-source OSP Suite, which ensures transparency, facilitates reproducibility, and encourages community collaboration. Indeed, the current work serves as a lighthouse example for OSP Suite-enabled collaborations across academia and industry.

The models for probenecid, rifampicin, and rosuvastatin were originally developed through industry-academic partnerships in a broader context (Hanke et al., 2018, Hanke et al., 2020; Wiebe et al., 2020). Additionally, the cyclosporine model was developed by a PBPK consulting CRO and sponsored by industry to investigate BCRP-mediated DDIs (Schaller et al., 2024). We repurposed these models and integrated them with new models for CP-I, atorvastatin, and pitavastatin to investigate OATP1B-mediated DDIs. This demonstrates the synergies leveraged from previous research and further validates the OSP platform’s capability for reliable DDI predictions.

However, certain limitations in the current model should be noted. The complexity of the PBPK model can pose challenges related to parameter identifiability, as has been described previously (Yoshikado et al., 2018; 2022; Kimoto et al., 2022). In addition, the model relies on assumptions regarding system-specific physiological parameters. Furthermore, the optimized parameters showed correlation between the possible intestinal re-absorption of CP-I and it’s synthesis rate and the bilary excretion. To investigate the impact of uncertainty in the key estimated parameter, a sensitivity analysis was performed on the rifampicin *in vivo* K_i,OATP1B_. This analysis demonstrated that reducing the K_i,OATP1B_ value had a similar influence on the simulated CP-I concentration-time profiles as reported in a comparable SIMCYP® model, producing a similar curve shape (Kimoto et al., 2022). Additionally, simulations confirmed that using a more mechanistically complex CP-I synthesis rate in MoBi® yielded results comparable to those from the simpler zero-order infusion approach used in PK-Sim®, validating the robustness of our modeling choice.

This work demonstrates that a qualified PBPK CP-I model offers a powerful approach for evaluating the risk of OATP1B-mediated DDIs. For a new drug exhibiting OATP inhibition potential, an *in vivo* K_i,OATP1B_ can be calibrated from early clinical data, and used to predict DDIs with sensitive objects. From a drug development perspective, this qualified model serves as a valuable quantitative tool with direct strategic applications. In line with the ICH M12 Guideline (FDA, 2026), early-stage clinical assessments of an investigational drug can include monitoring for effects on endogenous CP-I. If a clinically relevant increase in CP-I is detected, the framework presented here allows for the derivation of an *in vivo* Kᵢ,_OATP1B_, which can be applied to prospectively simulate DDIs with sensitive objects, notably statins. Such a model-informed approach may provide sufficient justification for including patients on statin therapy in later-stage trials without the need for a dedicated clinical DDI study and thereby conserving resources while minimizing participant burden.

Overall, this study establishes a qualified, transparent, and mechanistically detailed PBPK model of CP-I for the assessment of OATP1B-mediated DDIs. It provides a validated workflow for leveraging endogenous biomarker data to inform clinical development decisions and address critical drug safety considerations for novel therapeutics. Future efforts may focus on expanding the DDI network or adapting the model to explore the impact of intrinsic factors, such as pharmacogenomics and organ impairment on OATP1B function.

## 5 Conclusion

This study presents the successful development and qualification of a comprehensive PBPK DDI network for OATP1B1/3 transporters, anchored by a mechanistically detailed model of the endogenous biomarker CP-I. As the first CP-I model to be developed and made publicly available within the OSP Suite, it provides a transparent, reproducible, and freely accessible resource to the scientific community.

This qualified framework serves as a valuable asset for MIDD, enabling mitigation of OATP-mediated DDI risks for new therapeutic entities and supporting future DDI investigations. More broadly, this work highlights the strategic advantage of integrating endogenous biomarkers into DDI risk assessment. Using CP-I data from early-stage clinical trials (e.g., single rising or multiple rising studies) may provide a useful tool to inform DDI risk assessment and related development decisions. With further evaluation and broader dialogue among regulatory agencies, academia, and industry scientists, this approach could potentially support the waiver of certain dedicated clinical DDI studies and contribute to more efficient and mechanistically informed drug development strategies.

## Data Availability

The datasets presented in this study can be found in online repositories. The names of the repository/repositories and accession number(s) can be found in the article/Supplementary Material.
