# Supplementary material for: Coproporphyrin I PBPK model in the open systems pharmacology suite enables precise prediction of OATP 1B- mediated drug interactions through calibrated In vivo inhibition constants
Source: Front Pharmacol. 2026 Jul 28;17:1828033. doi: 10.3389/fphar.2026.1828033 (PMC13457544; doi:10.3389/fphar.2026.1828033)
Supplement: Supplementary file 1 [file DataSheet1.pdf]

Building and evaluation of a PBPK model for Coproporphyrin-I in healthy adults

Version	x.x-OSPy.y
based on *Model Snapshot* and *Evaluation Plan*	https://github.com/Open-Systems-Pharmacology/Coproporphyrin-I/releases/tag/vx.x (https://github.com/Open-Systems-Pharmacology/Coproporphyrin-I/releases/tag/vx.x)
OSP Version	y.y
Qualification Framework Version	z.z

This evaluation report and the corresponding PK-Sim project file are filed at:

https://github.com/Open-Systems-Pharmacology/OSP-PBPK-Model-Library/ (https://github.com/Open-Systems-Pharmacology/OSP-PBPK-Model-Library/)

Table of Contents

- 1 Introduction
- 2 Methods
  - 2.1 Modeling strategy
  - 2.2 Data used
  - 2.3 Model parameters and assumptions
- 3 Results and Discussion
  - 3.1 Coproporphyrin-I final input parameters
  - 3.2 Coproporphyrin-I Diagnostics Plots
  - 3.3 Concentration-Time Profiles
    - 3.3.1 Model Building
- 4 Conclusion
- 5 References

1 Introduction

Coproporphyrin-I is an endogenous biomarker for OATP1B1 transport, as this is the main route of excretion of Coproporphyrin-I in combination with elimination via MRP2 to the bile. In addition, a smaller fraction of Coproporphyrin-I is excreted through urine.

This Coproporphyrin-I model is intended to be used as an endogenous marker to estimate the inhibiton constants for OATP1B1 perpetrators.

This whole-body PBPK model of Coproporphyrin-I has been developed using published pharmacokinetic clinical data by Mori 2020 (Mori 2020), Takehara 2018 (Takehara 2018), Lai 2016 (Lai 2016) and Zhang 2020 (Zhang 2020). The model has then been evaluated by simulating clinical studies and comparing with respective observed data. The clinical data included both baseline and drug-drug interaction (DDI) studies.

The presented model includes the following features:

- transport by OATP1B1/B3,
- transport by MRP2

- renal clearance by glomerular filtration,
- intracellular liver synthesis modeled as a zero-order infusion.

2 Methods

2.1 Modeling strategy

The general concept of building a PBPK model has previously been described by Kuepfer et al. (Kuepfer 2016). Relevant information on anthropometric (height, weight) and physiological parameters (e.g. blood flows, organ volumes, binding protein concentrations, hematocrit, cardiac output) in adults was gathered from the literature and has been previously published (Willmann 2007). The information was incorporated into PK-Sim® and was used as default values for the simulations in adults.

The applied activity and variability of plasma proteins and active processes that are integrated into PK-Sim® are described in the publicly available PK-Sim® Ontogeny Database Version 7.3 (PK-Sim Ontogeny Database Version 7.3) or otherwise referenced for the specific process.

A mean model was built based on clinical data from studies of baseline Coproporphyrin-I and after administation of OATP1B perpetrators by Mori 2020 (Mori 2020), Takehara 2018 (Takehara 2018), Lai 2016 (Lai 2016) and Zhang 2020 (Zhang 2020). The studies reported mean plasma concentrations of Coproporphyrin-I. The mean PBPK model was developed using a mean individual based on the demographic data for each study. If no demographic data were provided, the following values were used: male, European, 30 years of age, 73 kg body weight and 176 cm body height. The relative tissue-specific expressions of the enzyme and transporter predominantly being involved in the metabolism/transport of Coproporphyrin-I (OATP1B1 and MRP2) were considered (Meyer 2012). A zero-order infusion of 1.09 mg/kg over one year were applied to model the synthesis rate of Coproporphyrin-I (Mochizuki 2022).

A specific set of parameters (see below) was optimized to describe the disposition of Coproporphyrin-I using the Parameter Identification module provided in PK-Sim®. Structural model selection was mainly guided by visual inspection and total error of the resulting description of data, 95% confidence interval of the identified parameter values and biological plausibility.

Details about input data (physicochemical, *in vitro* and clinical) can be found in Section 2.2.

Details about the structural model and its parameters can be found in Section 2.3.

2.2 Data used

2.2.1 In vitro and physicochemical data

A literature search was performed to collect available information on physicochemical properties of Coproporphyrin-I. The obtained information from literature is summarized in the table below, and is used for model building.

Parameter	Unit	Value	Source	Description
MW	g/mol	654.71	Yoshikado 2018	Molecular weight
pK_a (acid)		3.56	Yoshikado 2018	acid dissociation constant of conjugate acid
pK_a (base)		5.18	Yoshikado 2018	acid dissociation constant of conjugate base
Solubility (pH7.4)	mg/mL	0.03	DrugBank	Aqueous Solubility in pH 7.4

Parameter	Unit	Value	Source	Description
logP		2.53	Yoshikado 2018	Partition coefficient between octanol and water
fu	%	0.66	Yoshikado 2018	Fraction unbound in plasma
K_m OATP1B1	µmol/L	0.13	Bednarczy 2016	OATP1B1 Michaelis-Menten constant
K_m MRP2	µmol/L	7.7	Gilibili 2017	MRP2 Michaelis-Menten
Synthesis rate	nmol/h/kg	0.19	Mochizuki 2022	Endogenous synthesis rate
Renal clearance	L/h/kg	0.04	Mochizuki 2022	elimination rate via kidneys

2.2.2 Clinical data

A literature search was performed to collect available clinical data on Coproporphyrin-I in adults.

The following publications were found in adults for model building:

Publication	Arm / Treatment / Information used for model building
Mori 2020	Baseline plasma PK profiles in healthy subjects of Coproporphyrin-I
Takehara 2018	Baseline plasma PK profiles in healthy subjects of Coproporphyrin-I
Lai 2016	Baseline plasma PK profiles in healthy subjects of Coproporphyrin-I
Zhang 2020	Baseline plasma PK profiles in healthy subjects of Coproporphyrin-I

2.3 Model parameters and assumptions

2.3.1 Absorption

The parameter value for `Specific intestinal permeability` was set to 0 to not include intestinal reabsorption. This was informed by non-successful parameter identification's going to very low values and fixing the parameter to the calculated one resulted in unreasonable fits and the fraction excreted to feces was not consistent with literature (Section 2.2.1). The solubility was obtained from DrugBank (see Section 2.2.1).

The synthesis rate was modelled as a zero-order infusion over one year into intracellularly in the liver. This was informed by first modelling a synthesis rate in MoBi according to the value stated in literature and then translating it into a zero-order infusion in PK-Sim (Mochizuki 2022).

2.3.2 Distribution

Coproporphyrin-I is highly bound to plasma proteins (>99 %) (see Section 2.2.1). A value of 0.66% was used in this PBPK model for `Fraction unbound (plasma, reference value)`. The major binding partner was set to albumin (see Section 2.2.1).

An important parameter influencing the resulting volume of distribution is lipophilicity. The reported logP value of 2.53 (see Section 2.2.1) is used in this model.

The partition coefficient calculation by `Rodgers and Rowland` and cellular permeability calculation by `PK-Sim standard` was selected as distribution calculation methods.

2.3.3 Metabolism and Elimination

Two transport proteins were implemented into the model via Michaelis-Menten kinetics - OATP1B1 and MRP2.

- OATP1B1

The OATP1B1 expression profile is based on high-sensitive real-time RT-PCR (Nishimura 2003). The reference concentration for OATP1B1 was measured by liquid chromatography tandem mass spectroscopy (Prasad 2013), i.e not according to the default implementation in PK-Sim. Transporter activity was described as saturable process following Michaelis-Menten kinetics, were the `Km` was taken from literature and the `kcat` was optimized based on clinical data (see Section 2.3.4).

- MRP2

The MRP2 expression profiles is based on high-sensitive real-time RT-PCR (Nishimura 2003). The reference concentration for MRP2 was measured by liquid chromatography tandem mass spectroscopy (Deo 2012), i.e not according to the default implementation in PK-Sim. Transporter activity was described as saturable process following Michaelis-Menten kinetics, were the `Km` was taken from literature and the `kcat` was optimized based on clinical data (see Section 2.3.4).

Additionally, glomerular filtration rate fraction was set to 1 as this describes the observed renal clearance rate in accordance to literature (see Section 2.2.1).

2.3.4 Automated Parameter Identification

The following parameters were optimized by fitting the model to the data:

Model Parameter
`kcat` (OATP1B1)
`kcat` (MRP2)

3 Results and Discussion

The PBPK model for Coproporphyrin-I was developed and verified with clinical pharmacokinetic data.

The model was built and evaluated covering data from studies including in particular

- baseline endogenous Coproporphyrin-I data.
- DDIs with OATP1B1 perpetrators rifampicin and probenecid (in OATP1B1/3-DDI qualification report).

The model quantifies syntethis of Coproporphyrin-I in the liver, excretion via urine through glomerular filtration, influx to liver via OATP1B1/1B3 and excretion to bile via MRP2.

Of note, the suggested workflow when assessing DDIs using Coproporphyrin-I is to re-estimate the baseline Coproporphyrin-I for the specific study as the baseline will vary across studies.

The next sections show:

1. the final model input parameters for the building blocks: Section 3.1.
2. the overall goodness of fit: Section 3.2.
3. simulated vs. observed concentration-time profiles for the clinical studies used for model building: Section 3.3, model verification plots will be available in the OATP1B1/3-DDI qualification report.

3.1 Coproporphyrin-I final input parameters

The compound parameter values of the final PBPK model are illustrated below.

Compound: Coproporphyrin I

Parameters

Name	Value	Value Origin	Alternative	Default
Solubility at reference pH	0.0309 mg/ml	Database-DrugBank	Measurement	True
Reference pH	7	Database-DrugBank	Measurement	True
Lipophilicity	2.53 Log Units	Publication-Yoshikado 2018	Measurement	True
Fraction unbound (plasma, reference value)	0.659 %	Publication-Yoshikado 2018	Measurement	True
Specific intestinal permeability (transcellular)	0 cm/min	Parameter Identification-Parameter Identification-Value updated from ‘Parameter Identification 3 - Pint=0, GFR’ on 2024-03-06 09:36	PI	True
Is small molecule	Yes			
Molecular weight	654.71 g/mol	Publication-Yoshikado 2018		
Plasma protein binding partner	Albumin			

Calculation methods

Name	Value
Partition coefficients	Rodgers and Rowland
Cellular permeabilities	PK-Sim Standard

Processes

Transport Protein: MRP2-PI

Molecule: MRP2

Parameters

Name	Value	Value Origin
Transporter concentration	1 µmol/l	
Vmax	0 µmol/l/min	
Km	7.7 µM	Publication-In Vitro-Gilibili et al. 2017 - Coproporphyrin-I: A Fluorescent, Endogenous Optimal Probe Substrate for ABCC2 (MRP2) Suitable for Vesicle-Based MRP2 Inhibition Assay

Name	Value	Value Origin
kcat	105.171 1/min	Parameter Identification-Parameter Identification-Value updated from ‘Parameter Identification 3 -Pint=0, GFR in vivo Ki final-SA’ on 2024-03-08 15:12

Transport Protein: OATP1B1-PI

Molecule: OATP1B1

Parameters

Name	Value	Value Origin
Transporter concentration	1 µmol/l	
Vmax	0 µmol/l/min	
Km	0.13 µM	
kcat	88.588 1/min	Parameter Identification-Parameter Identification-Value updated from ‘Parameter Identification 3 -Pint=0, GFR in vivo Ki final-SA’ on 2024-03-08 15:12

Systemic Process: Glomerular Filtration-GFR

Species: Human

Parameters

Name	Value	Value Origin
GFR fraction	1	Publication-Mochizuki 2022

Advanced Protocol: Liver periportal intracellular

Schema Item 1 User Defined Placeholder for formulation: Formulation Start time: 0 h Dose: 1.09 mg/kg #### Parameters

Name	Value	Value Origin
Start time	0 h	
NumberOfRepetitions	1	
TimeBetweenRepetitions	0 h	
Start time	0 h	
InputDose	1.089699324 mg/kg	

Formulation: 1 yr zero order

Type: Zero Order

Parameters

Name	Value	Value Origin
End time	8760 h	

3.2 Coproporphyrin-I Diagnostics Plots

Below you find the goodness-of-fit visual diagnostic plot (observed versus simulated plasma concentration) for the PBPK model performance of all data used presented in Section 2.2.2. Note that the model represents one mean state with constant endogenous concentrations, and this explains the horizontal spread of the residuals in the plot.

3.3 Concentration-Time Profiles

Simulated versus observed concentration-time profiles of all data listed in Section 2.2.2 are presented below.

3.3.1 Model Building

Figure 3-1: Lai 2016 - baseline

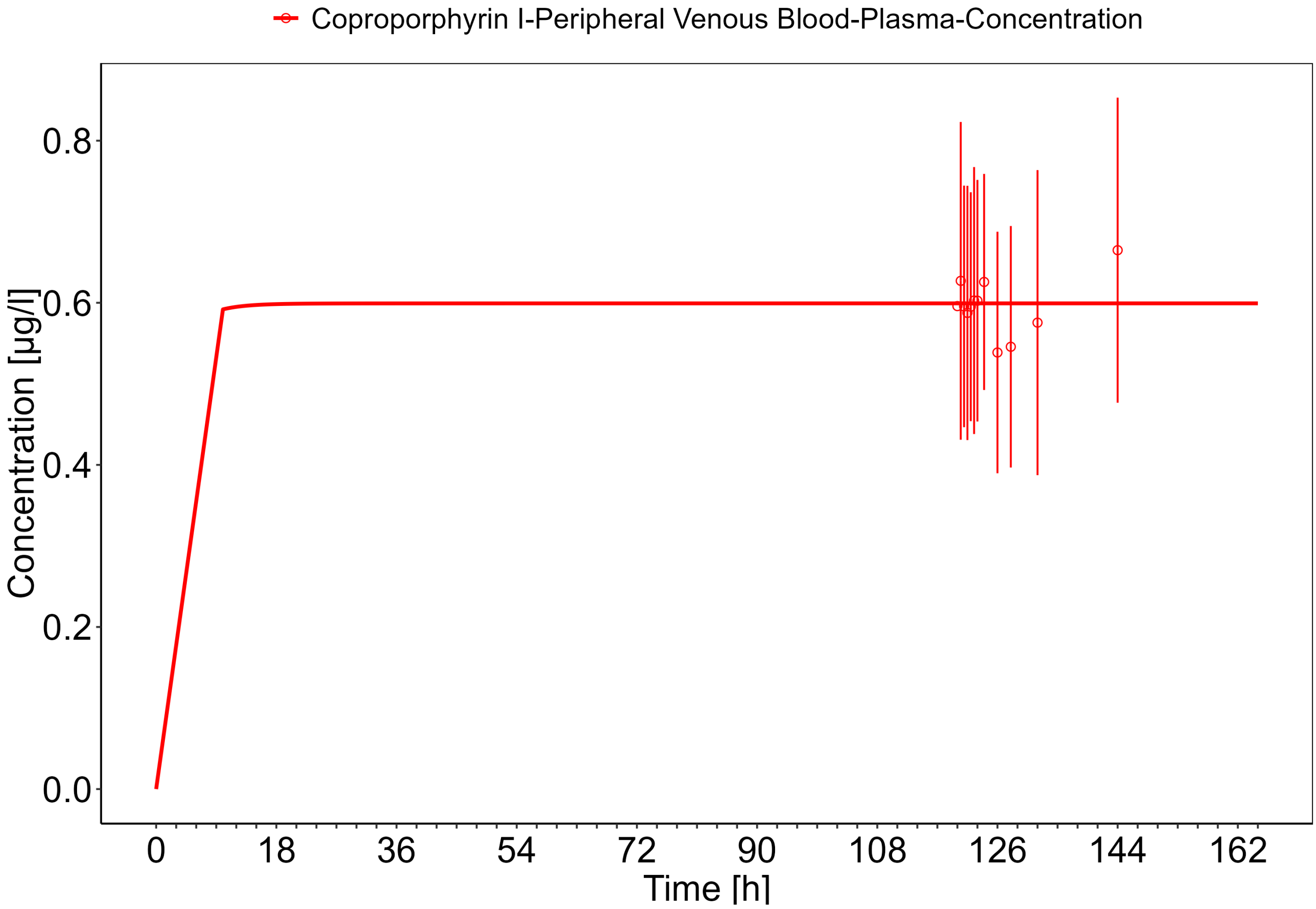

Figure 3-2: Takehara 2018 - baseline

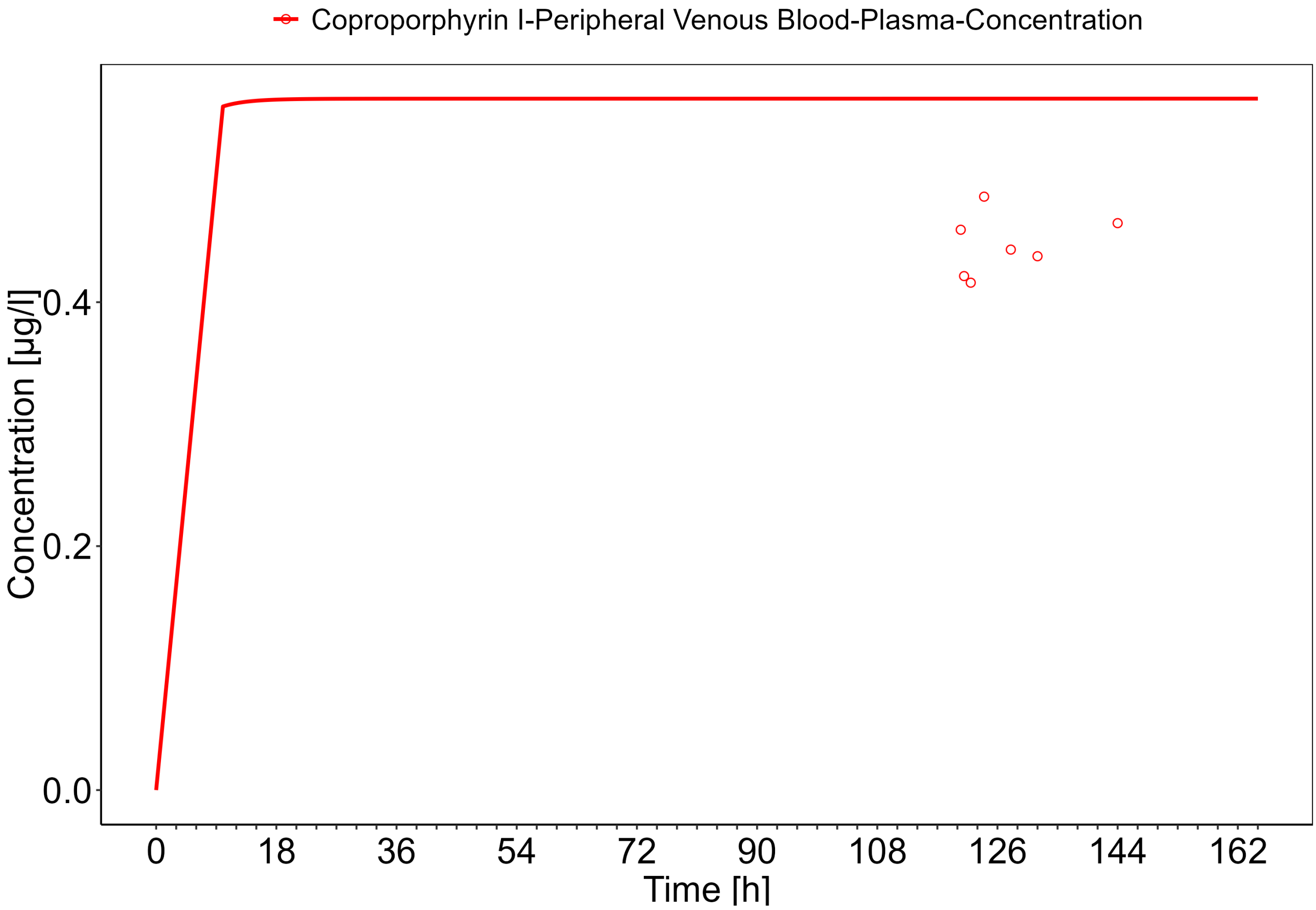

Figure 3-3: Zhang 2020 - baseline

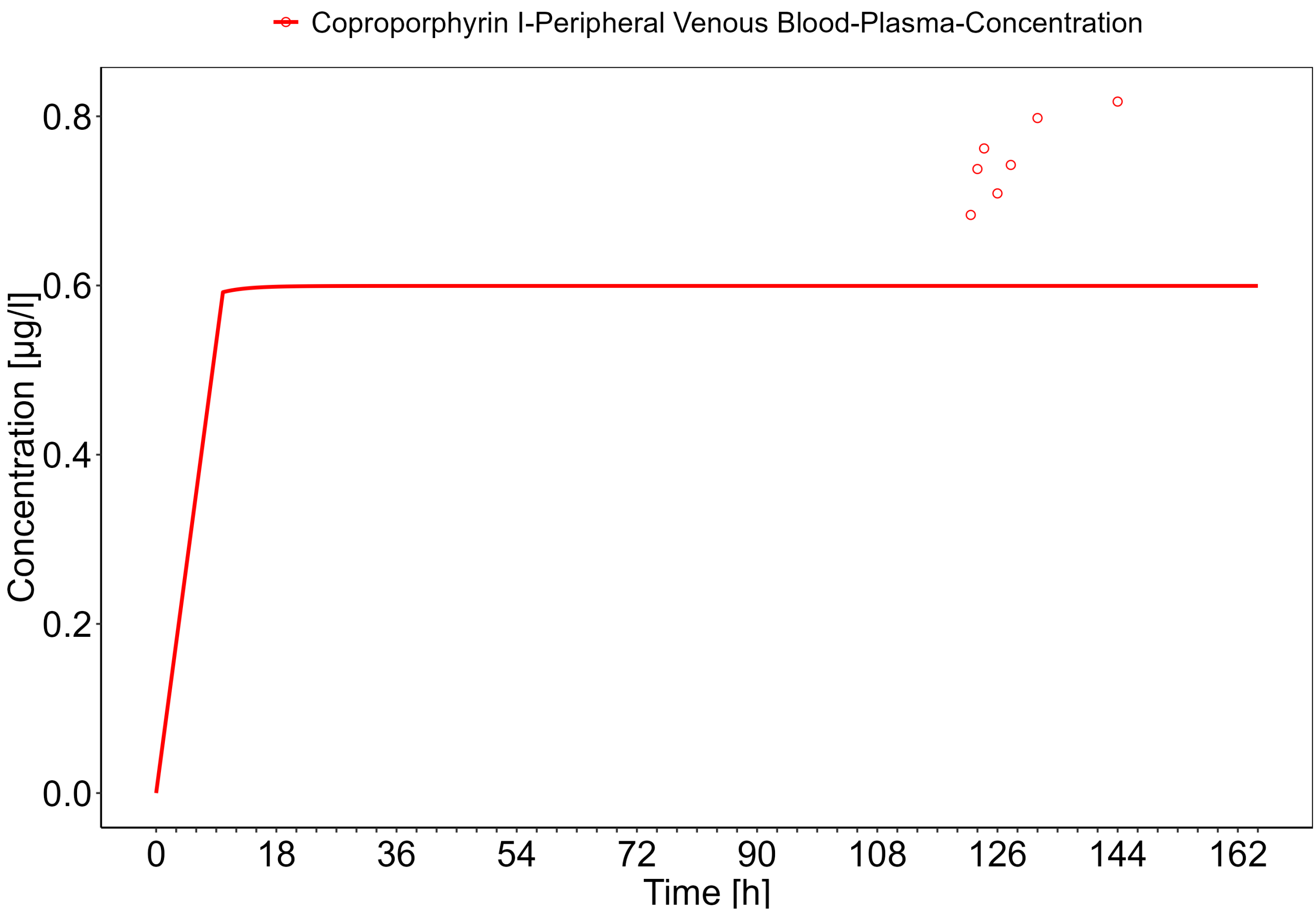

Figure 3-4: Mori 2020 - baseline

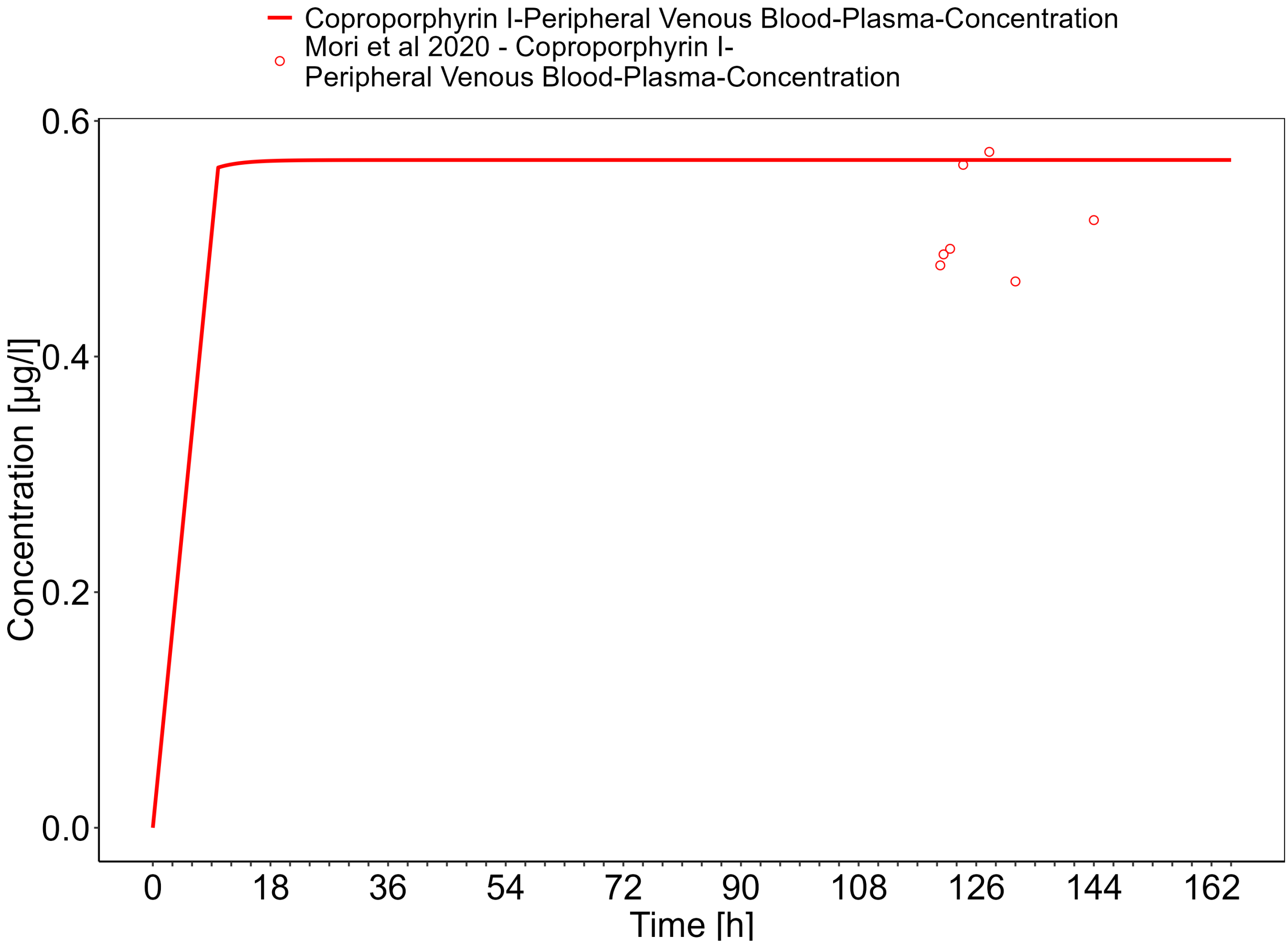

4 Conclusion

The presented PBPK model adequately describes the endogenous baseline of Coproporphyrin-I in adults.

5 References

Bednarczy 2016 Bednarczyk D, Boiselle C. Organic anion transporting polypeptide (OATP)-mediated transport of coproporphyrins I and III. Xenobiotica. 2016 May 3;46(5):457-66.

Deo 2012 Deo AK, Prasad B, Balogh L, Lai Y, Unadkat JD. Interindividual variability in hepatic expression of the multidrug resistance-associated protein 2 (MRP2/ABCC2): quantification by liquid chromatography/tandem mass spectrometry. Drug metabolism and disposition: the biological fate of chemicals. 2012. 40(5):852–5

Drugbank Knox C, Wilson M, Klinger CM, et al. DrugBank 6.0: the DrugBank Knowledgebase for 2024. Nucleic Acids Res. 2024 Jan 5;52(D1):D1265-D1275. doi: 10.1093/nar/gkad976

Gilibili 2017 Gilibili RR, Chatterjee S, Bagul P, Mosure KW, Murali BV, Mariappan TT, Mandlekar S, Lai Y. Coproporphyrin-I: a fluorescent, endogenous optimal probe substrate for ABCC2 (MRP2) suitable for vesicle-based MRP2 inhibition assay. Drug Metabolism and Disposition. 2017 Jun 1;45(6):604-11.

Kimoto 2022 E Kimoto, C Costales, MA West, YA Bi, M Vourvahis, A David Rodrigues, MVS Varma (2022). Biomarker-Informed Model-Based Risk Assessment of Organic Anion Transporting Polypeptide 1B Mediated Drug-Drug Interactions. *Clin Pharmacol Ther*, Feb;111(2):404-415.

Lai 2016 Lai Y, Mandlekar S, Shen H, Holenarsipur VK, Langish R, Rajanna P, Murugesan S, Gaud N, Selvam S, Date O, Cheng Y. Coproporphyrins in plasma and urine can be appropriate clinical biomarkers to recapitulate drug-drug interactions mediated by organic anion transporting polypeptide inhibition. Journal of Pharmacology and Experimental Therapeutics. 2016 Sep 1;358(3):397-404.

Lou 2015 Z Luo, Y Zhang, J Gu, P Feng, Y Wang (2015). Pharmacokinetic Properties of Single- and Multiple-Dose Pitavastatin Calcium Tablets in Healthy Chinese Volunteers. *Curr Ther Res Clin Exp*, Mar 3;77:52-7.

Meyer 2012 Meyer M, Schneckener S, Ludewig B, Kuepfer L, Lippert J. (2012). Using expression data for quantification of active processes in physiologically based pharmacokinetic modeling. *Drug Metab Dispos*. May;40(5), 892-901.

Mochizuki 2022 T Mochizuki, Y Aoki, T Yoshikado, K Yoshida, Y Lai, H Hirabayashi, Y Yamaura, K Rockich, K Taskar, T Takashima, X Chu, MJ Zamek-Gliszczynski, J Mao, K Maeda, K Furihata, Y Sugiyama, H Kusuhara (2022). Physiologically-based pharmacokinetic model-based translation of OATP1B-mediated drug-drug interactions from coproporphyrin I to probe drugs. *Clin Transl Sci.* Jun;15(6):1519-1531.

Mori 2020 D Mori, E Kimoto, B Rago, Y Kondo, A King-Ahmad, R Ramanathan, LS Wood, JG Johnson, VH Le, M Vourvahis, A David Rodrigues (2020). Dose-dependent inhibition of OATP1B by rifampicin in healthy volunteers: comprehensive evaluation of candidate biomarkers and OATP1B probe drugs. *Clinical Pharmacology & Therapeutics*, Apr;107(4):1004-13.

Nishimura 2003 Nishimura M, Yaguti H, Yoshitsugu H, Naito S, Satoh T. (2003). Tissue distribution of mRNA expression of human cytochrome P450 isoforms assessed by high-sensitivity real-time reverse transcription PCR. *Yakugaku Zasshi.* May;123(5), 369-75.

Prasad 2013 Prasad B, Lai Y, Lin Y, Unadkat JD (2013) Interindividual variability in the hepatic expression of the human breast cancer resistance protein (BCRP/ABCG2): effect of age, sex, and genotype. Journal of pharmaceutical sciences 102(3):787–93

Prasad 2014 Prasad B, Evers R, Gupta A, Hop CECA, Salphati L, Shukla S, Ambudkar SV, Unadkat JD (2014) Interindividual variability in hepatic organic anion-transporting polypeptides and PGlycoprotein (ABCB1) protein expression: Quantification by liquid chromatography tandem mass spectroscopy and influence of genotype, age, and sex. Drug Metabolism and Disposition 42(1):78–88

Takehara 2018 I Takehara, T Yoshikado, K Ishigame, D Mori, KI Furihata, N Watanabe, O Ando, K Maeda, Y Sugiyama, H Kusuhara (2018). Comparative study of the dose-dependence of OATP1B inhibition by rifampicin using probe drugs and endogenous substrates in healthy volunteers. *Pharmaceutical research*, Jul;35:1-3.

Yoshikado 2018 Yoshikado T, Toshimoto K, Maeda K, Kusuhara H, Kimoto E, Rodrigues AD, Chiba K, Sugiyama Y. PBPK modeling of coproporphyrin I as an endogenous biomarker for drug interactions involving inhibition of hepatic OATP1B1 and OATP1B3. CPT: pharmacometrics & systems pharmacology. 2018 Nov;7(11):739-47.

Zhang 2020 Zhang Y, Holenarsipur VK, Kandoussi H, Zeng J, Mariappan TT, Sinz M, Shen H. Detection of Weak Organic Anion–Transporting Polypeptide 1B Inhibition by Probenecid with Plasma-Based Coproporphyrin in Humans. Drug Metabolism and Disposition. 2020 Oct 1;48(10):841-8.
