# Supplementary material for: Coproporphyrin I PBPK model in the open systems pharmacology suite enables precise prediction of OATP 1B- mediated drug interactions through calibrated In vivo inhibition constants
Source: Front Pharmacol. 2026 Jul 28;17:1828033. doi: 10.3389/fphar.2026.1828033 (PMC13457544; doi:10.3389/fphar.2026.1828033)
Supplement: Supplementary file 2 [file DataSheet2.pdf]

Building and evaluation of a PBPK model for Pitavastatin in healthy adults

Version	x.x-OSPy.y
based on *Model Snapshot* and *Evaluation Plan*	https://github.com/Open-Systems-Pharmacology/Pitavastatin-Model/releases/tag/vx.x
OSP Version	y.y
Qualification Framework Version	z.z

This evaluation report and the corresponding PK-Sim project file are filed at:

https://github.com/Open-Systems-Pharmacology/OSP-PBPK-Model-Library/

Table of Contents

- 1 Introduction
- 2 Methods
  - 2.1 Modeling strategy
  - 2.2 Data used
  - 2.3 Model parameters and assumptions
- 3 Results and Discussion
  - 3.1 Pitavastatin final input parameters
  - 3.2 Pitavastatin Diagnostics Plots
  - 3.3 Concentration-Time Profiles
    - 3.3.1 Model Building
    - 3.3.2 Model Verification
- 4 Conclusion
- 5 References

1 Introduction

Pitavastatin is an inhibitor of HMG-CoA reductase and indicated for hypercholesterolaemia and for the prevention of cardiovascular disease. Pitavastatin is an orally delivered drug with a bioavailability of about 80%. Pitavastatin is mainly excreted via the bile via uptake into the liver by OATP1Bs and efflux to bile via BCRP, renal excretion is negligible.

This pitavastatin model is intended to be used as a victim drug in OATP1B1-mediated drug-drug interactions (DDI).

This whole-body PBPK model of pitavastatin has been developed using published pharmacokinetic clinical data by Mori 2020 (Mori 2020), Takehara 2018 (Takehara 2018), Lou 2015 (Lou 2015), Kimoto 2022 (Kimoto 2022), Chen 2013 (Chen 2013) and Prueksaritanont 2014 (Prueksaritanont 2014). The clinical data includedboth intravenous and orally administration and a dose range of 0.2mg to 4 mg pitavastatin. The model has then been evaluated by simulating clinical studies with individuals of different SLO1B1 genotypes and comparing with respective observed data.

The presented model includes the following features:

- metabolism by unspecified UGT enzym present in the liver,
- transport by OATP1B1/B3,
- transport by BCRP
- no renal clearance,
- oral absorption with dissolution rate assigned to the Lint80 function..

2 Methods

2.1 Modeling strategy

The general concept of building a PBPK model has previously been described by Kuepfer et al. (Kuepfer 2016). Relevant information on anthropometric (height, weight) and physiological parameters (e.g. blood flows, organ volumes, binding protein concentrations, hematocrit, cardiac output) in adults was gathered from the literature and has been previously published (Willmann 2007). The information was incorporated into PK-Sim® and was used as default values for the simulations in adults.

The applied activity and variability of plasma proteins and active processes that are integrated into PK-Sim® are described in the publicly available PK-Sim® Ontogeny Database Version 7.3 (PK-Sim Ontogeny Database Version 7.3) or otherwise referenced for the specific process.

A mean model was built based on clinical data from studies with intravenous and oral administration of pitavastatin by Mori 2020 (Mori 2020), Takehara 2018 (Takehara 2018), Lou 2015 (Lou 2015), Kimoto 2022 (Kimoto 2022), Chen 2013 (Chen 2013) and Prueksaritanont 2014 (Prueksaritanont 2014). The studies reported mean plasma concentrations of pitavastatin. The mean PBPK model was developed using a mean individual based on the demographic data for each study and if no demographic data were provided the following values were used; male, European, 30 years of age, 73 kg body weight and 176 cm body height. The relative tissue-specific expressions of the enzyme and transporter predominantly being involved in the metabolism/transport of pitavastatin (OATP1B1 and BCRP) were considered (Meyer 2012). A Lint80 function with 80% of tablet dossilved in 30 minutes were applied according to Cho 2023 (Cho 2024).

Due to the large inter-study variability of pitavastatin plasma concentration, a large part of the available clinical data was used for model building.

A specific set of parameters (see below) was optimized to describe the disposition of pitavastatin using the Parameter Identification module provided in PK-Sim®. Structural model selection was mainly guided by visual inspection and total error of the resulting description of data, 95% confidence interval of the identified parameter values and biological plausibility.

The model was then verified by simulating further clinical studies reporting pharmacokinetic concentration-time profiles after oral administration of pitavastatin in individuals with different SLCO1B1 genotypes.

Details about input data (physicochemical, *in vitro* and clinical) can be found in Section 2.2.

Details about the structural model and its parameters can be found in Section 2.3.

2.2 Data used

2.2.1 In vitro and physicochemical data

A literature search was performed to collect available information on physicochemical properties of pitavastatin. The obtained information from literature is summarized in the table below, and is used for model building.

**Parameter**	**Unit**	**Value**	Source	**Description**
MW	g/mol	421.46	Yoshikado 2018	Molecular weight
pKa (acid)		4.24	Yoshikado 2018	acid dissociation constant of conjugate acid
pKa (base)		4.68	Yoshikado 2018	acid dissociation constant of conjugate base
Solubility (pH7.4)	mg/mL	0.004	DrugBank	Aqueous Solubility in pH 7.4
logP		1.92	Yoshikado 2018	Partition coefficient between octanol and water
		2.91	Kimoto 2022	Partition coefficient between octanol and water
fu	%	0.52	Yoshikado 2018	Fraction unbound in plasma
Km OATP1B1	µmol/L	0.77	Duan 2017	OATP1B1 Michaelis-Menten constant

**Parameter**	**Unit**	**Value**	Source	**Description**
Km BCRP	µmol/L	2.02	Assumed same as for Rosuvastatin	BCRP Michaelis-Menten
Lint80 Dissolution time	min	30	Cho 2024	Dissolution time until 80% of drug is dissolved
GFR fraction	fraction	0	Mochizuki 2022	glomerular filtration rate

2.2.2 Clinical data

A literature search was performed to collect available clinical data on pitavastatin in adults.

The following publications were found in adults for model building:

Publication	Arm / Treatment / Information used for model building
Kimoto 2022	Plasma PK profiles in healthy subjects after single iv infusion (1h) of 2 mg pitavastatin and oral administration of 2 and 4 mg pitavastatin
Mori 2020	Plasma PK profiles in healthy subjects after single oral administration of 0.2 mg pitavastatin
Takehara 2018	Plasma PK profiles in healthy subjects after single oral administration of 0.2 mg pitavastatin
Lou 2015	Plasma PK profiles in healthy subjects after single oral administration of 1, 2 and 4 mg pitavastatin
Chen 2013	Plasma PK profiles in healthy subjects after single oral administration of 4 mg pitavastatin
Prueksaritanont 2014	Plasma PK profiles in healthy subjects after single oral administration of 1 mg pitavastatin

The following dosing scenarios were simulated and compared to respective data for model verification:

Scenario	Data reference
po 2 mg in 3 different SLCO1B1 genotypes	Ieiri 2007

2.3 Model parameters and assumptions

2.3.1 Absorption

The parameter value for `Specific intestinal permeability` was optimized based on clinical oral data, see results of optimization in Section 2.3.4. The solubility was obtianed from DrugBank (see Section 2.2.1)

The dissolution of tablets was implemented via empirical Lint80 dissolution, according to Cho 2023 (Cho 2024).

2.3.2 Distribution

Pitavastatin is highly bound to plasma proteins (>99 %) (see Section 2.2.1). A value of 0.52% was used in this PBPK model for `Fraction unbound (plasma, reference value)`. The major binding partner was set to albumin (see Section 2.2.1).

An important parameter influencing the resulting volume of distribution is lipophilicity. The reported experimental logP values are in the range of 1.92-2.91 (see Section 2.2.1) which served as a starting value. Finally, the model parameters `Lipophilicity` was optimized to match clinical data (see also Section 2.3.4).

After testing the available organ-plasma partition coefficient and cell permeability calculation methods built in PK-Sim, observed clinical data was best described by choosing the partition coefficient calculation by `Rodgers and Rowland` and cellular permeability calculation by `Charge dependent Schmitt`.

2.3.3 Metabolism and Elimination

One metabolic pathway was implement into the model a first order process

- Unspecific UGT Metabolic enzyme activity is described as first order process

The unspecific UGT was set to only be expressed in the liver. Metabolic enzyme activity was set to be described as first order process, were the `CLspec/[Enzyme]` was optimized based on clinical data (see Section 2.3.4).

And two transport protein was implemented into the model via Michaelis-Menten kinetics

- OATP1B1

The OATP1B1 expression profiles is based on high-sensitive real-time RT-PCR (Nishimura 2003). The reference concentration for OATP1B1 was measured by liquid chromatography tandem mass spectroscopy (Prasad 2014). Transporter activity was described as saturable process following Michaelis-Menten kinetics, were the `Km` was taken from literature and the `kcat` was optimized based on clinical data (see Section 2.3.4).

- BCRP

The BCRP expression profiles is based on Microarray expression data from ArrayExpress. The reference concentration for OATP1B1 was measured by liquid chromatography tandem mass spectroscopy (Prasad 2013). Transporter activity was described as saturable process following Michaelis-Menten kinetics, were the `Km` was taken from literature and the `kcat` was optimized based on clinical data (see Section 2.3.4).

Additionally, renal clearance was set to 0 according to literature (see Section 2.2.1).

2.3.4 Automated Parameter Identification

The following parameters were optimized by fitting the model to the data::

Model Parameter
`Lipophilicity`
`kcat` (OATP1B1)
`CLspec/[Enzyme]` (OAT3)
`kcat` (BCRP)
`Specific intestinal permeability`

3 Results and Discussion

The PBPK model for pitavastatin was developed and verified with clinical pharmacokinetic data.

The model was built and evaluated covering data from studies including in particular

- single intravenous administration and single oral administrations (tablets).
- a dose range of 0.2 to 4 mg.

The model quantifies excretion via bile through BCRP transport, influx to liver via OATP1B1/1B3 and metabolism via unspecified UGT enzyme present in the liver.

The next sections show:

1. the final model input parameters for the building blocks: Section 3.1.
2. the overall goodness of fit: Section 3.2.

3. simulated vs. observed concentration-time profiles for the clinical studies used for model building and for model verification: Section 3.3.

3.1 Pitavastatin final input parameters

The compound parameter values of the final PBPK model are illustrated below.

Compound: Pitavastatin

Parameters

Name	Value	Value Origin	Alternative	Default
Solubility at reference pH	0.004 mg/ml	Database-DrugBank	Measurement	True
Reference pH	7	Database-DrugBank	Measurement	True
Lipophilicity	0.9683645282 Log Units	Parameter Identification-Parameter Identification-Value updated from 'PI-final' on 2024-03-18 16:36	fit	True
Fraction unbound (plasma, reference value)	0.52 %	Parameter Identification-Parameter Identification-Value updated from 'PI#3c_MC_JE_5' on 2024-03-07 12:12	Measurement	True
Specific intestinal permeability (transcellular)	0.0140921913 cm/min	Parameter Identification-Parameter Identification-Value updated from 'PI-final' on 2024-03-18 16:37	fit	True
F	1	Publication-Yoshikado 2018		
Is small molecule	Yes			
Molecular weight	421.46 g/mol	Publication-Yoshikado 2018		
Plasma protein binding partner	Albumin			

Calculation methods

Name	Value
Partition coefficients	Rodgers and Rowland
Cellular permeabilities	Charge dependent Schmitt

Processes

Transport Protein: BCRP-PI Molecule: BCRP

Parameters

Name	Value	Value Origin
Transporter concentration	1 µmol/l	
Vmax	0 µmol/l/min	
Km	2.02 µmol/l	Other-In Vitro-Hanke et al Assuming similar Km as for Rosuvastatin
kcat	0.3826135266 1/min	Parameter Identification-Parameter Identification-Value updated from 'PI-final' on 2024-03-18 16:36

Transport Protein: OATP1B1/1B3-PI Molecule: OATP1B1/1B3

Parameters

Name	Value	Value Origin
Transporter concentration	1 µmol/l	
Vmax	0 µmol/l/min	
Km	0.77 µmol/l	Publication-In Vitro-Duan et al 2017
kcat	739.176 1/min	Parameter Identification-Parameter Identification-Value updated from 'PI-final' on 2024-03-18 16:36 and correction OSP to Hanke

Systemic Process: Glomerular Filtration-GFR Species: Human

Parameters

Name	Value	Value Origin
GFR fraction	0	Other-Assumption

Metabolizing Enzyme: UGT-fit Molecule: UGT

Parameters

Name	Value	Value Origin
In vitro CL/recombinant enzyme	0 µl/min/pmol rec. enzyme	
CLspec/[Enzyme]	0.0220585031 l/µmol/min	Parameter Identification-Parameter Identification-Value updated from 'PI-final' on 2024-03-18 16:37

Transport Protein: OATP1B1/1B3-genotype SLCO1B1__1b__15 Molecule: OATP1B1/1B3

Parameters

Name	Value	Value Origin
Transporter concentration	1 µmol/l	
Vmax	0 µmol/l/min	
Km	0.77 µmol/l	Publication-In Vitro-Duan et al 2017
kcat	369.588 1/min	Parameter Identification-Assumption-half if PI fitted activity

Transport Protein: OATP1B1/1B3-genotype SLCO1B1__15__15 Molecule: OATP1B1/1B3

Parameters

Name	Value	Value Origin
Transporter concentration	1 µmol/l	

Name	Value	Value Origin
Vmax	0 µmol/l/min	
Km	0.77 µmol/l	Publication-In Vitro-Duan et al 2017
kcat	184.794 1/min	Parameter Identification-Assumption-1/4 of PI actitivity

3.2 Pitavastatin Diagnostics Plots

Below you find the goodness-of-fit visual diagnostic plots for the PBPK model performance of all data used presented in Section 2.2.2.

The first plot shows observed versus simulated plasma concentration, the second weighted residuals versus time.

Table 3-1: GMFE for Goodness of fit plot for concentration in plasma.

Group	GMFE
Pitavastatin iv (model building)	1.31
Pitavastatin oral administration (model building)	2.04
Pitavastatin oral administration (model verification)	1.42
All	1.76

Figure 3-1: Goodness of fit plot for concentration in plasma.

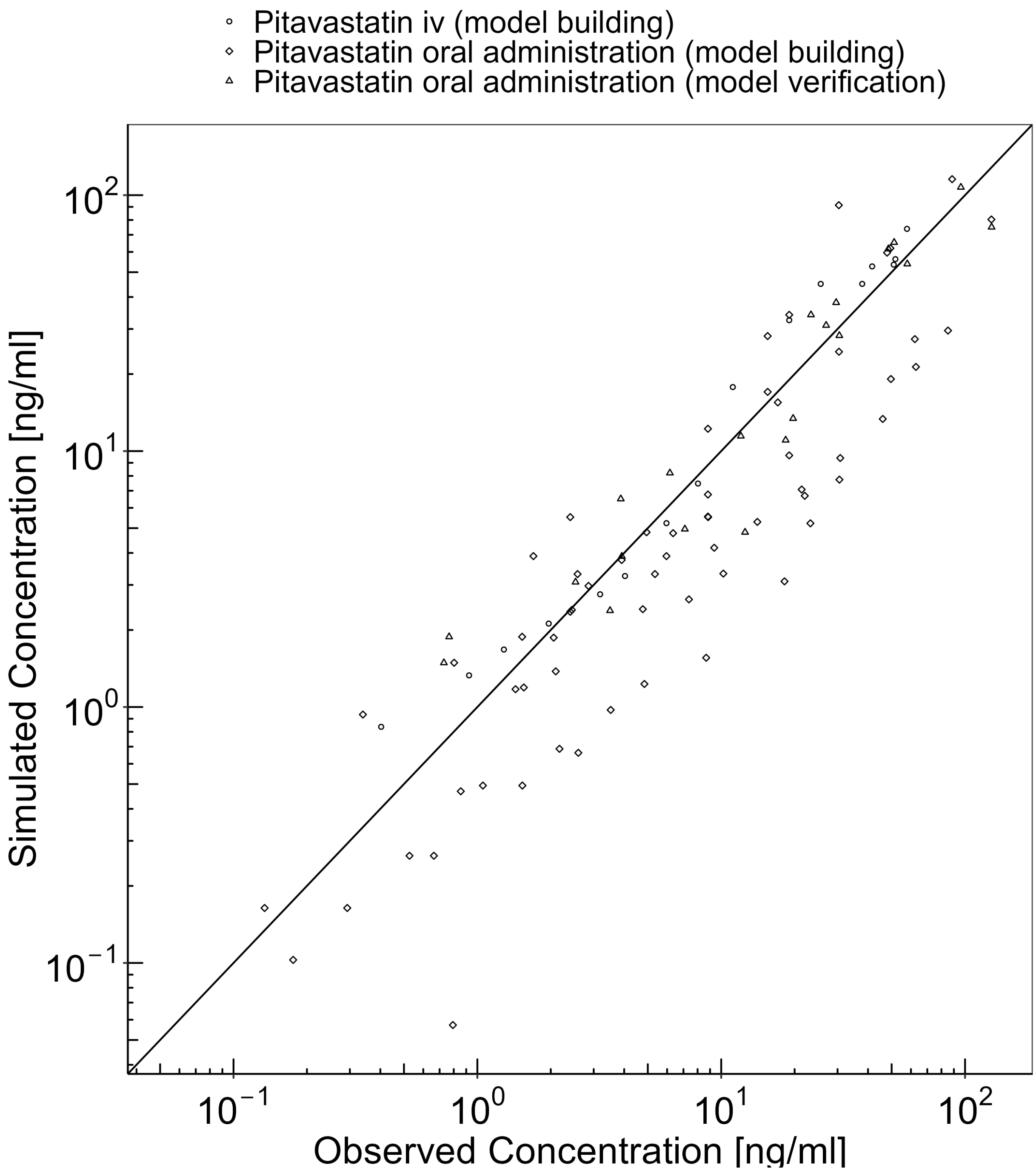

Figure 3-2: Goodness of fit plot for concentration in plasma.

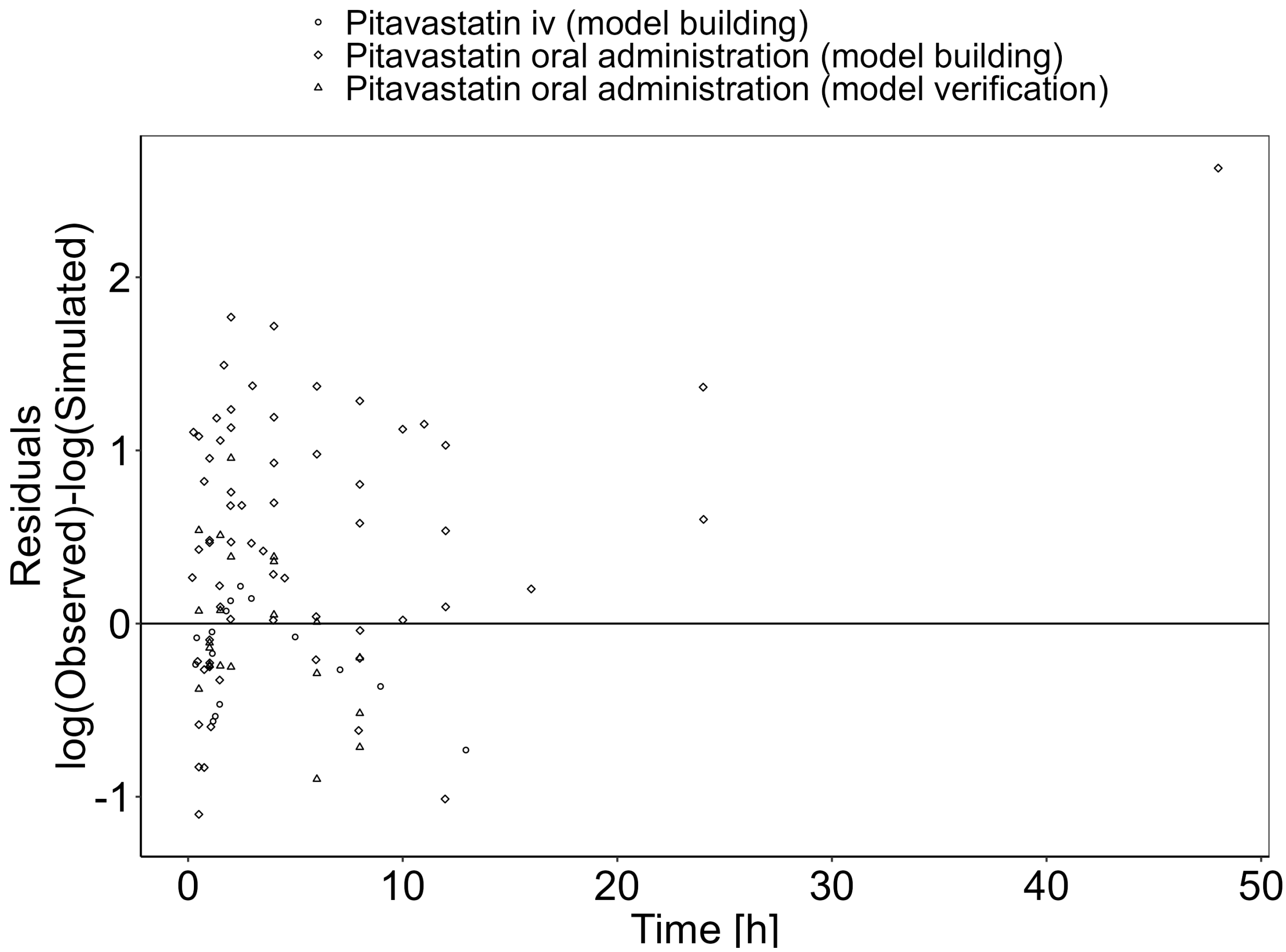

3.3 Concentration-Time Profiles

Simulated versus observed concentration-time profiles of all data listed in Section 2.2.2 are presented below.

3.3.1 Model Building

Figure 3-3: Chen et al. 2013 po 4 mg

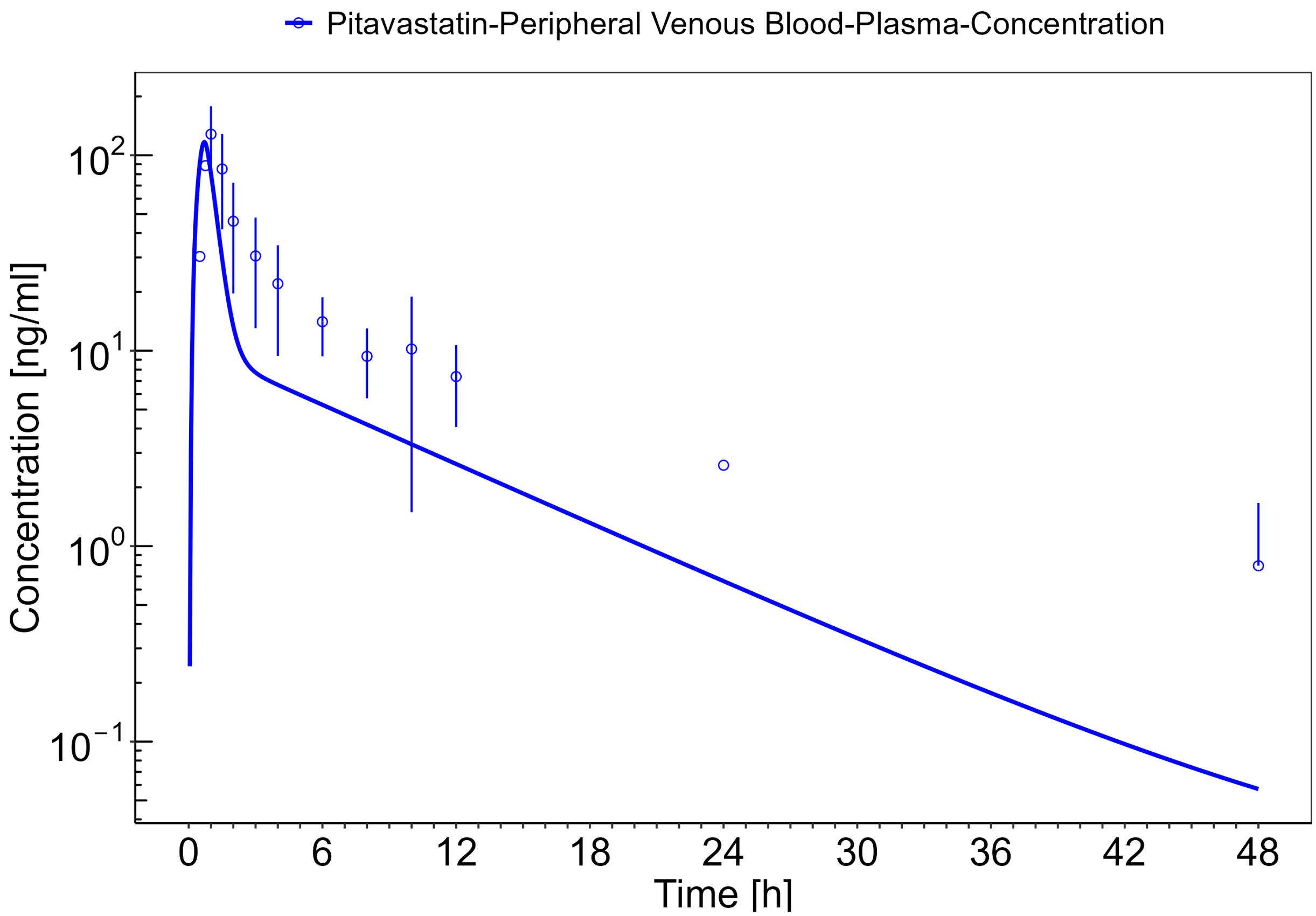

Figure 3-4: Kimoto et al 2022 - Pitavastatin 2 mg iv infusion

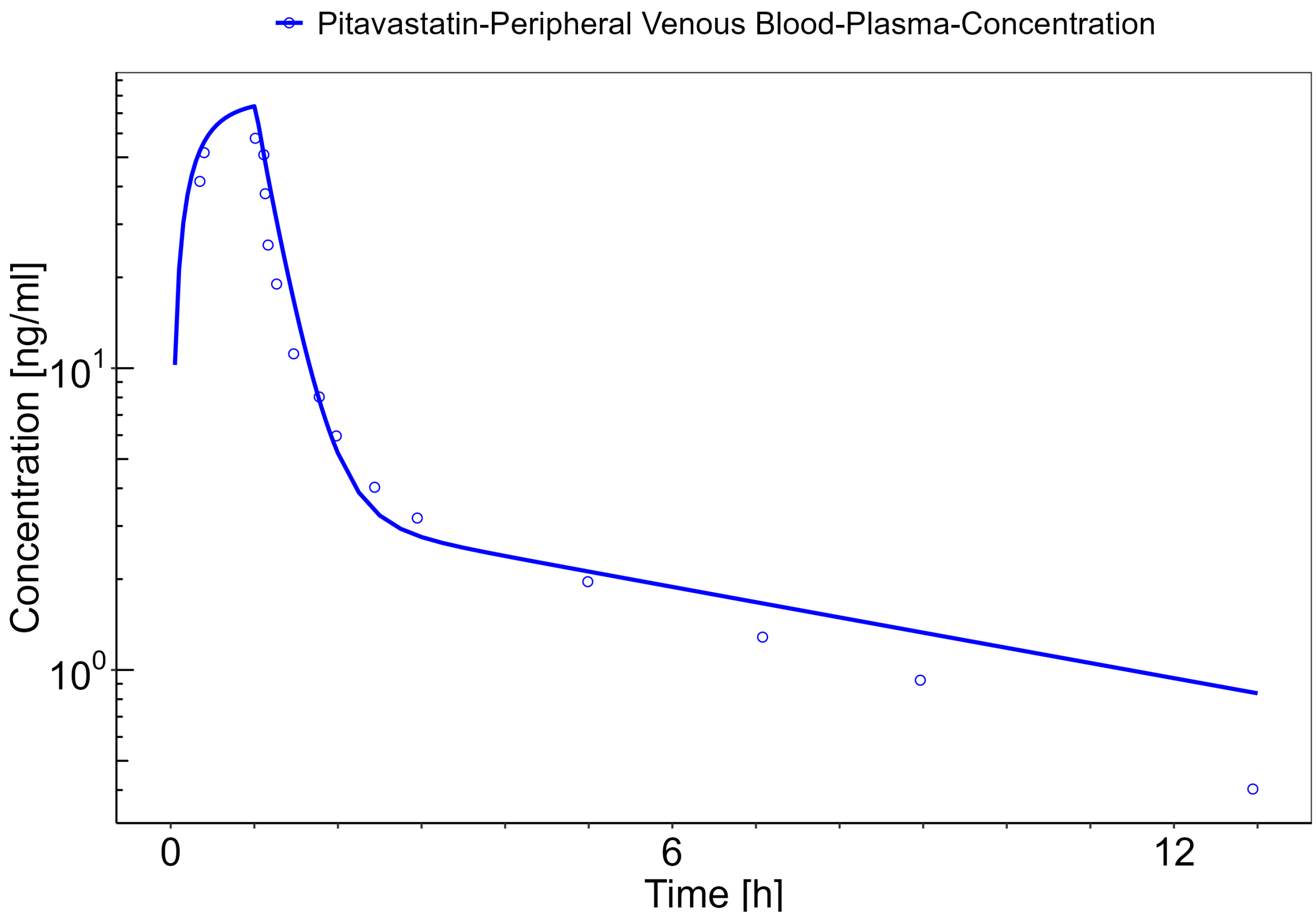

Figure 3-5: Kimoto et al 2022 - Pitavastatin po 2mg

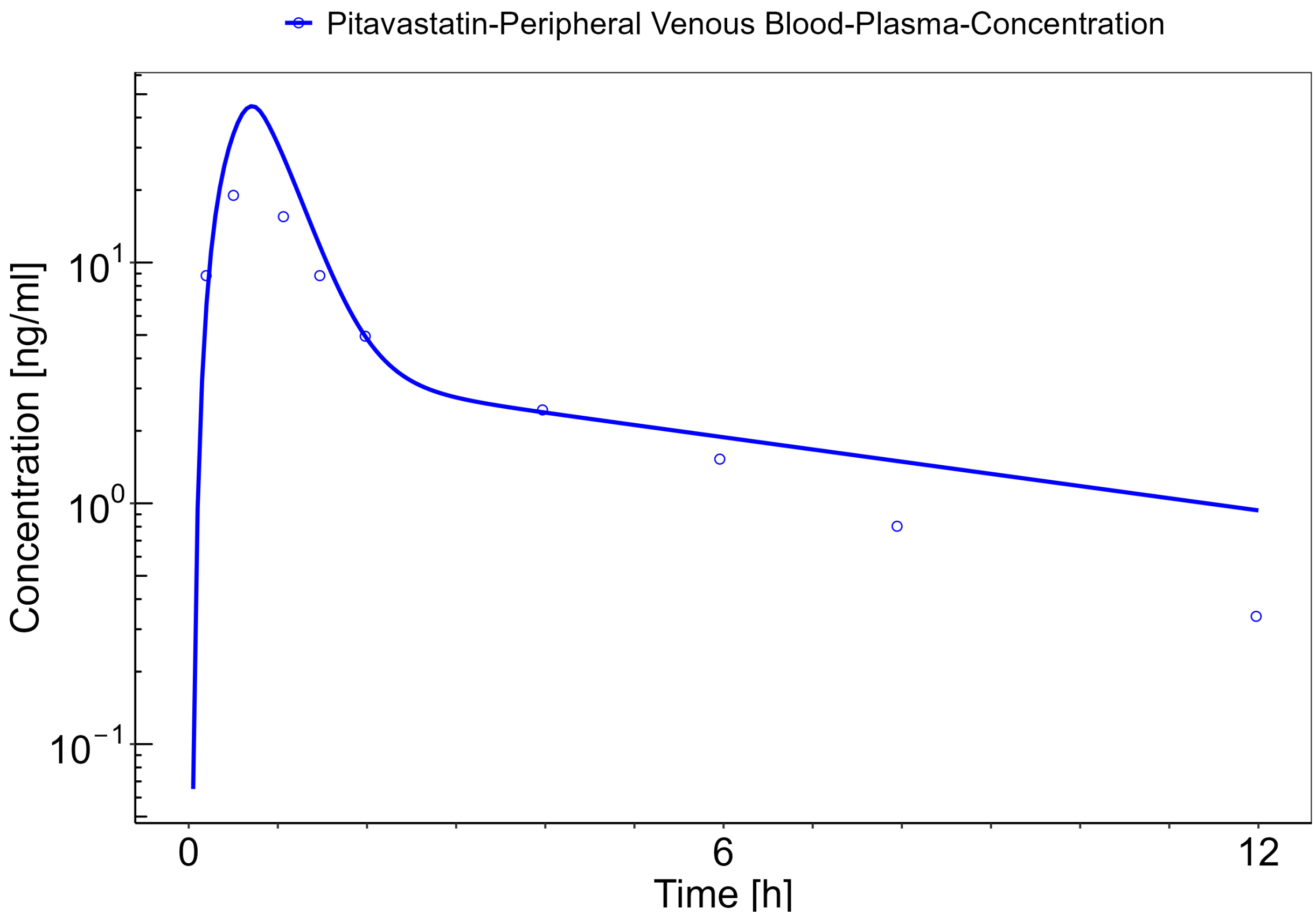

Figure 3-6: Kimoto et al 2022 - Pitavastatin po 4mg

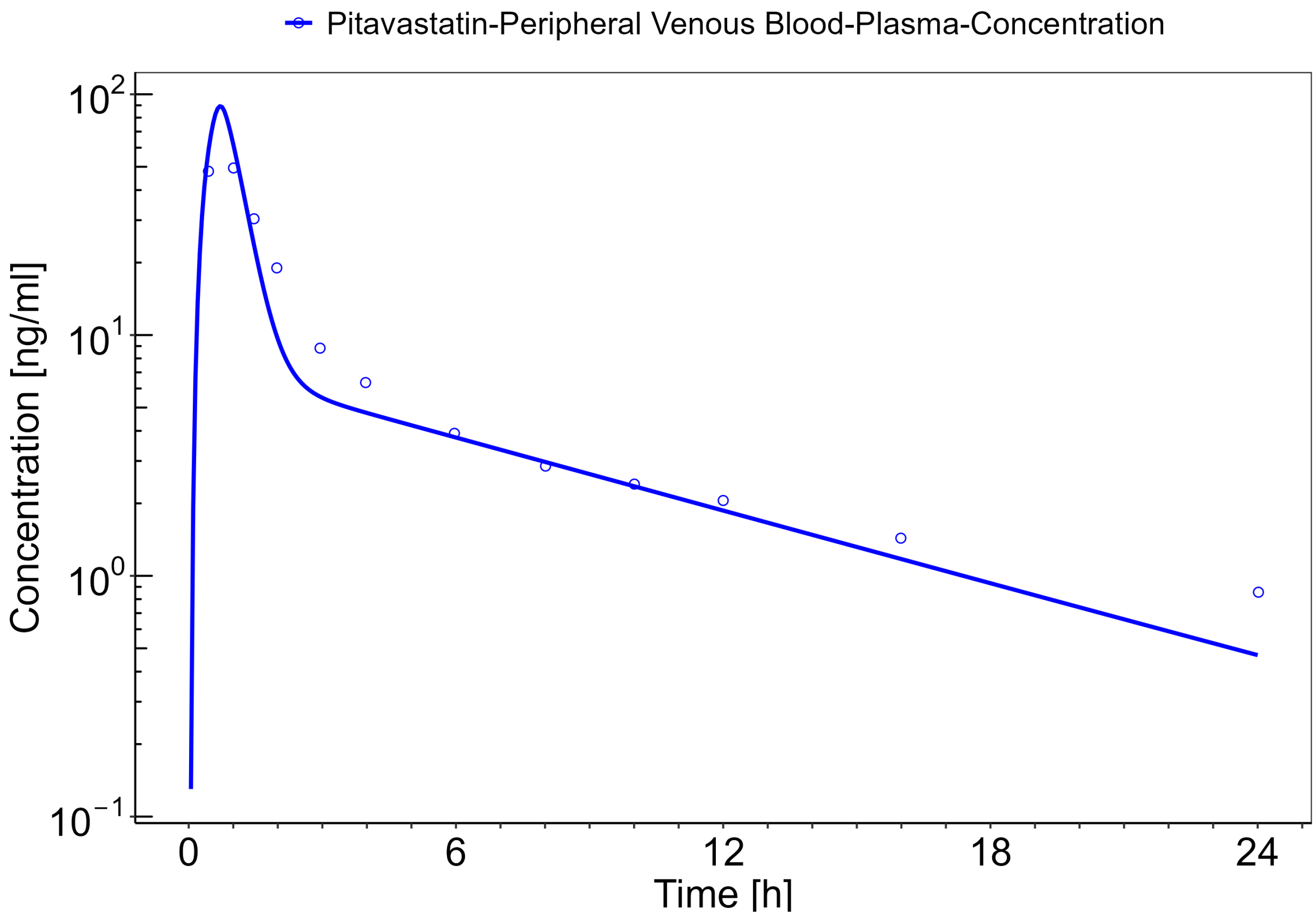

Figure 3-7: Lou et al. 2015 - Pitavastatin po 1 mg

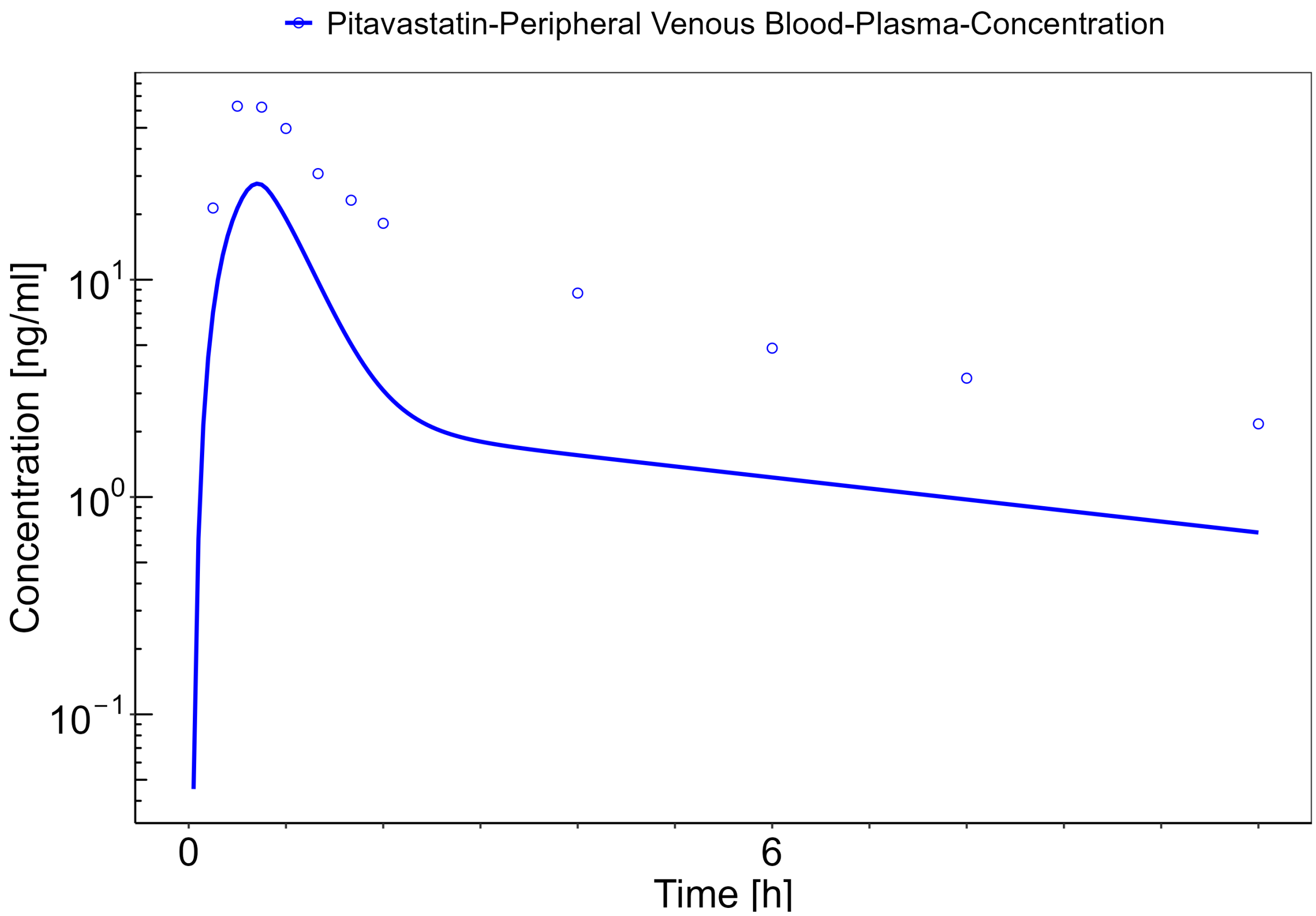

Figure 3-8: Mori et al 2020 - Pitavastatin po 0.2 mg

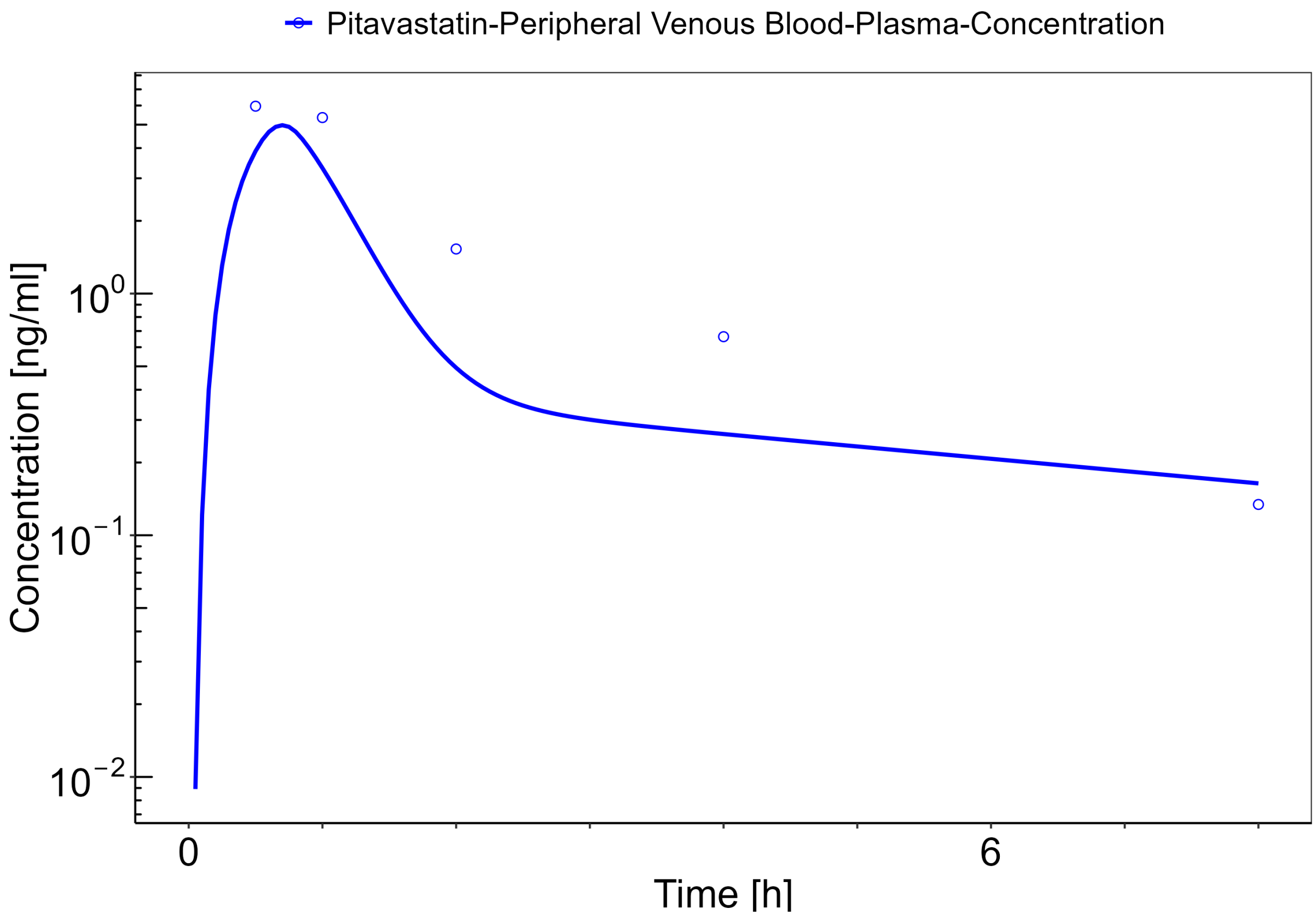

Figure 3-9: Prueksaritanont et al. 2014 - Pitavastatin po 1 mg

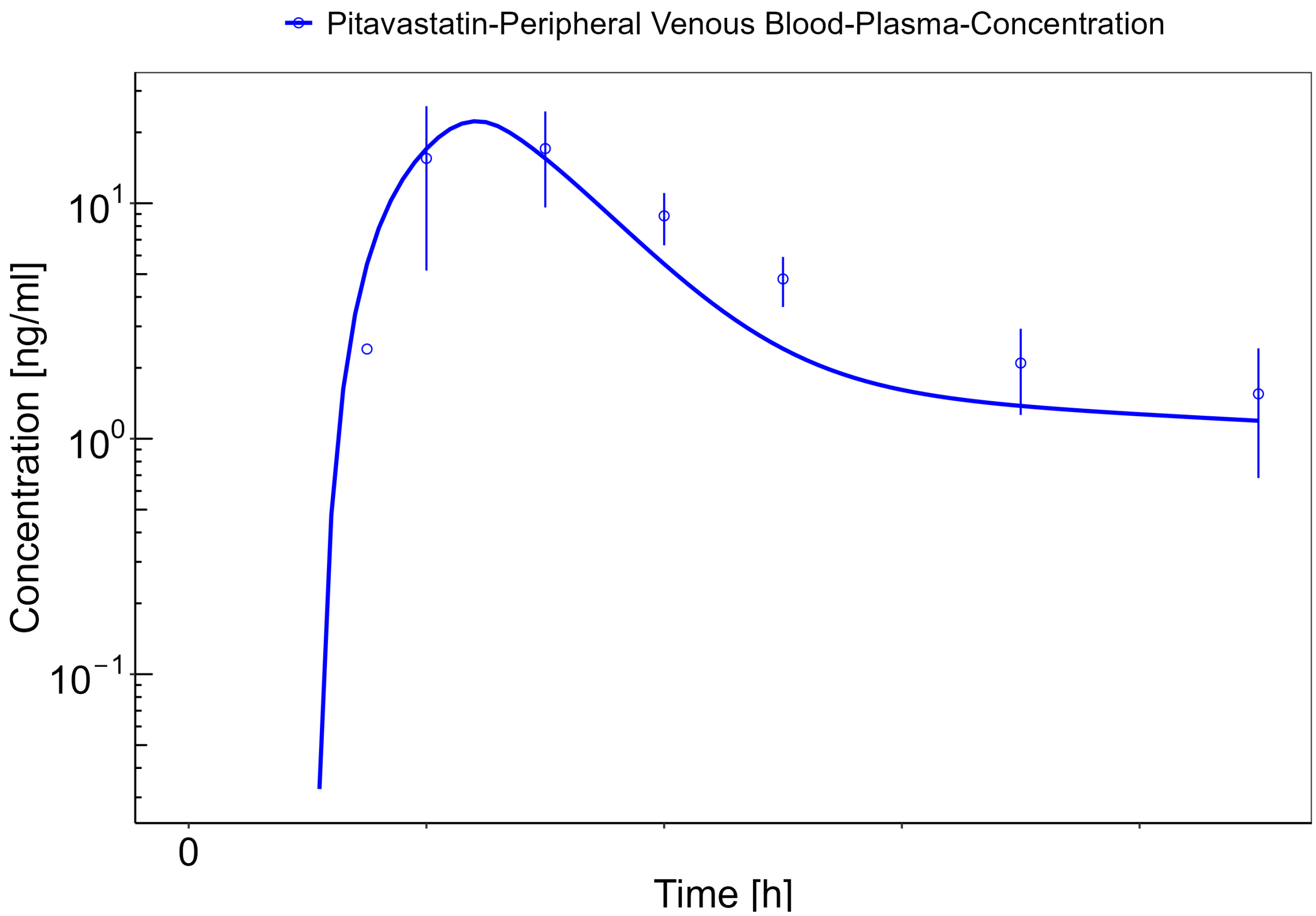

Figure 3-10: Takehara et al. 2018 - Pitavastatin po 0.2 mg

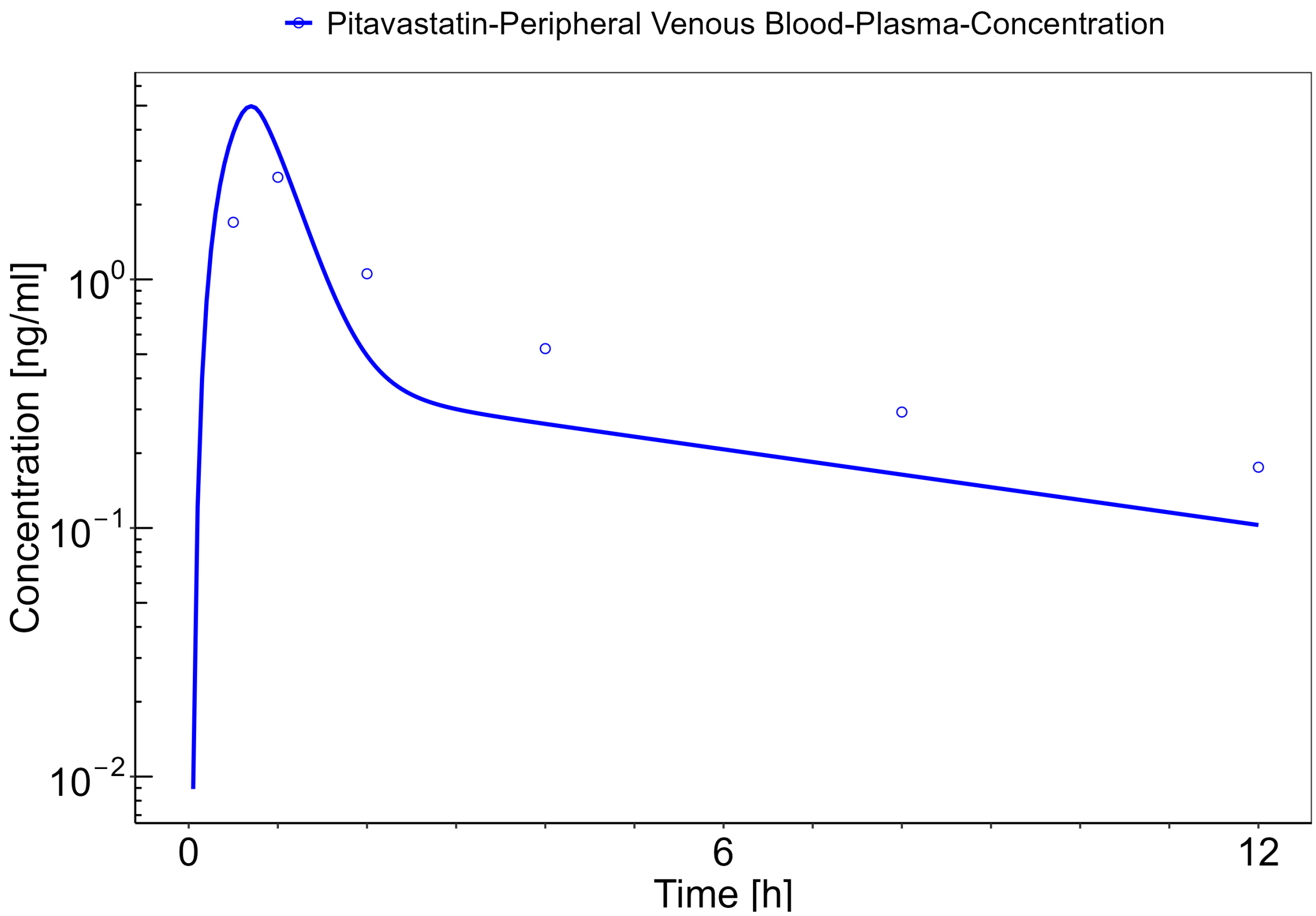

3.3.2 Model Verification

Figure 3-11: Ieiri et al. 2007 - Pitavastatin 2mg in SLCO1B1*15*15 subjects

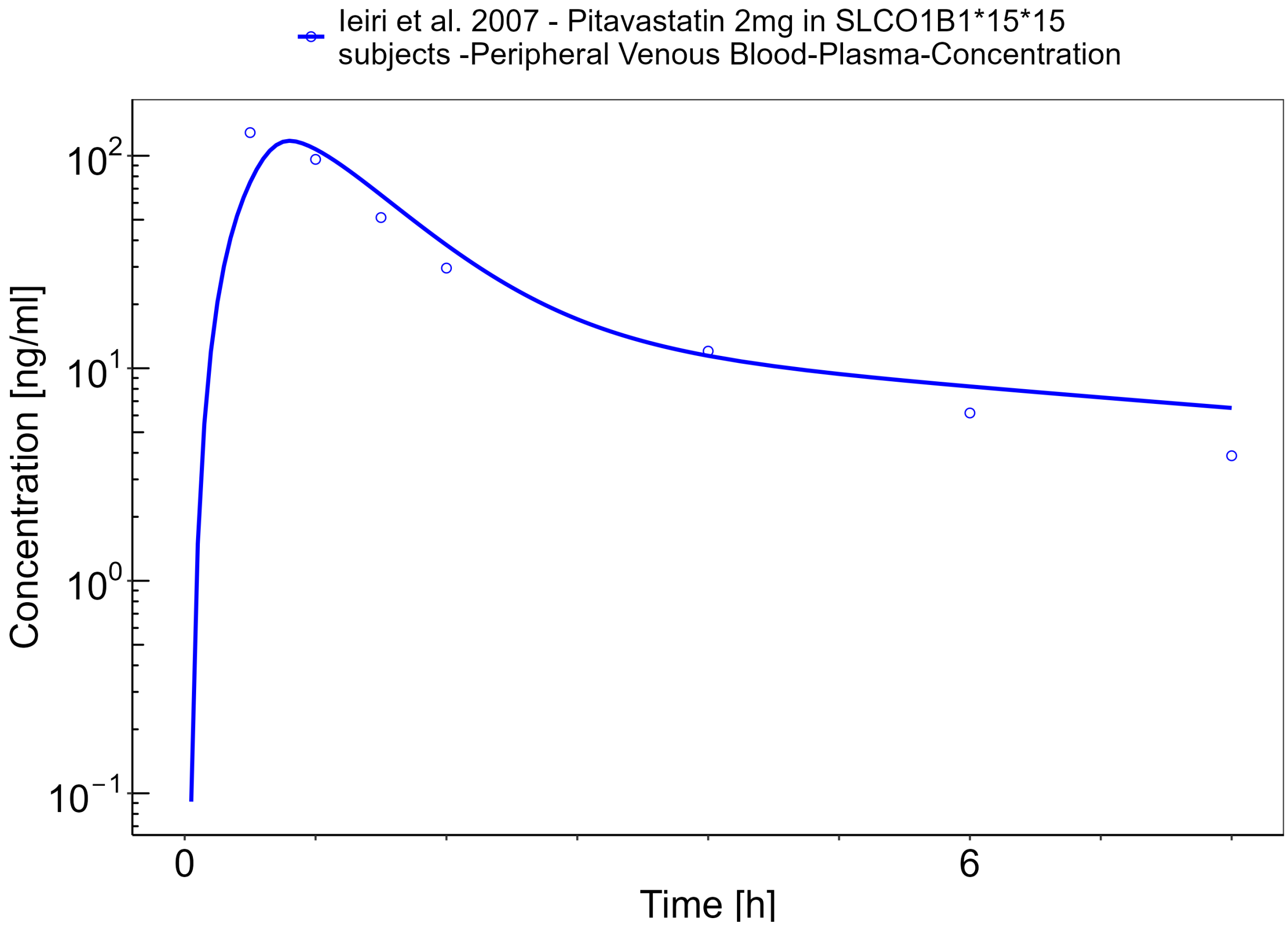

Figure 3-12: Ieiri et al. 2007 - Pitavastatin 2mg in SLCO1B1*1b*15 subjects

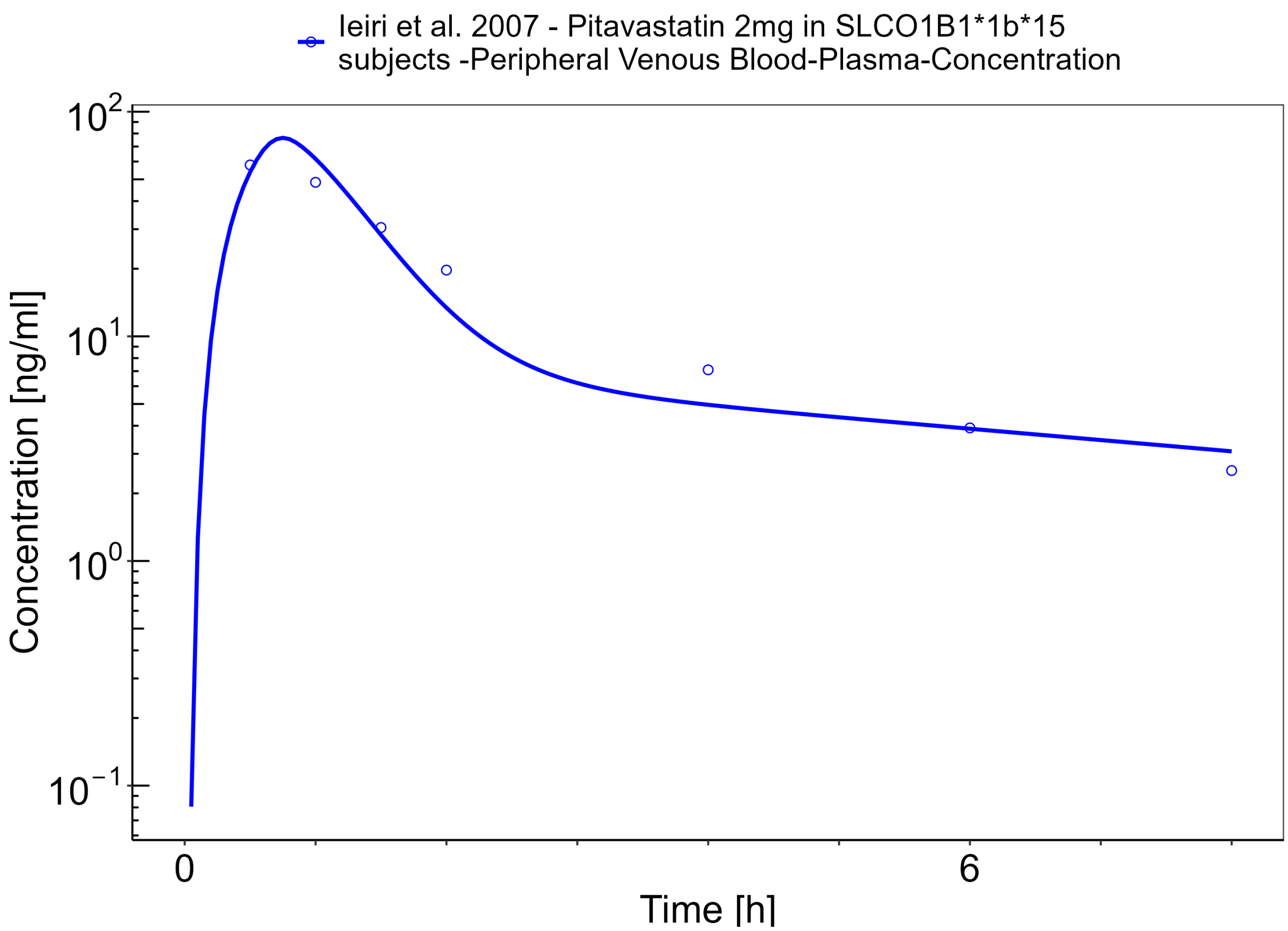

Figure 3-13: Ieiri et al. 2007 - Pitavastatin 2mg in SLCO1B1*1b*1b subjects

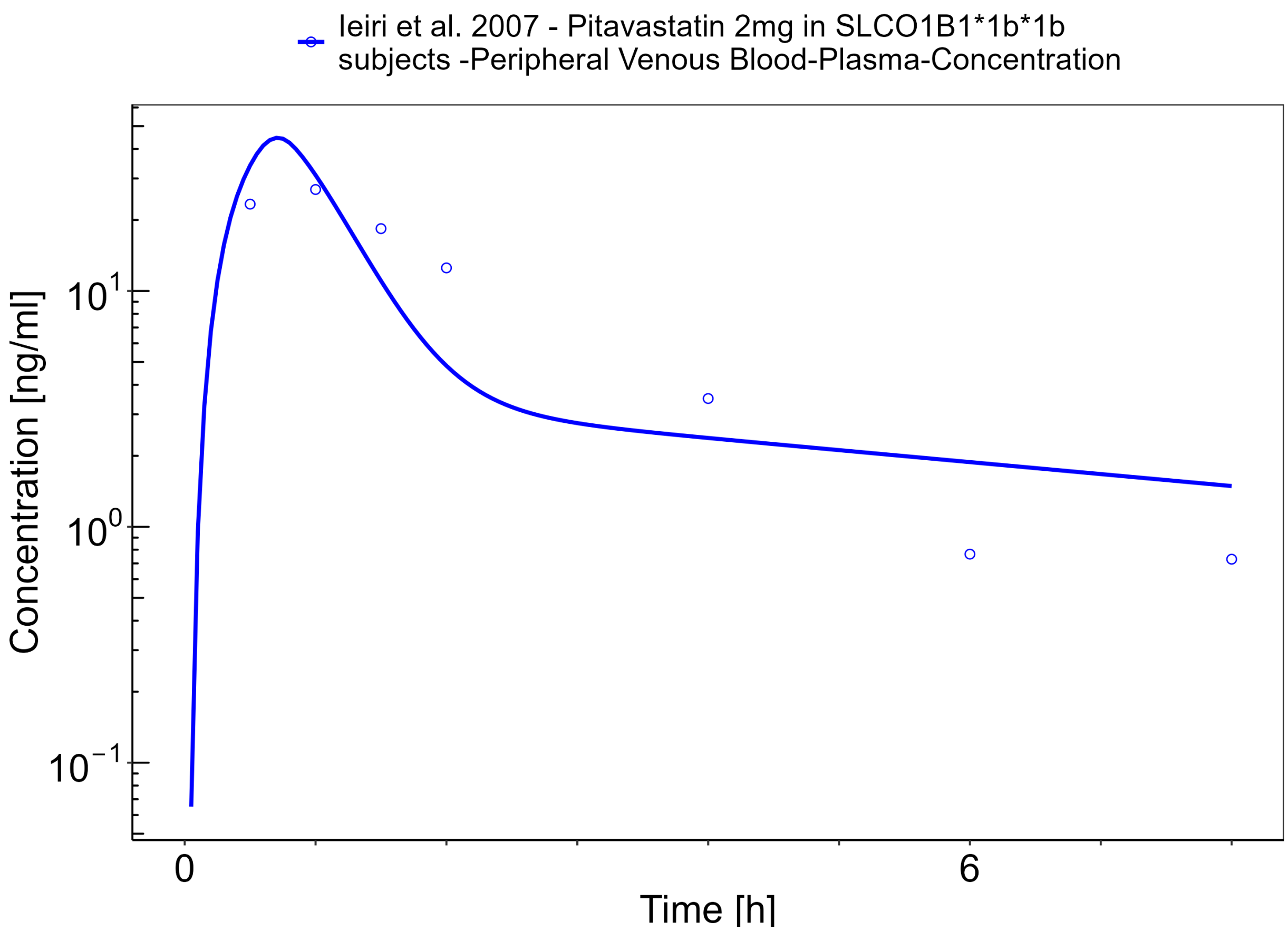

4 Conclusion

The presented PBPK model adequately describes the intravenous and oral pharmacokinetics of pitavastatin in adults.

5 References

Chen 2013 Y Chen, W Zhang, WH Huang, ZR Tan, YC Wang, X Huang, HH Zhou (2013). Effect of a single-dose rifampin on the pharmacokinetics of pitavastatin in healthy volunteers. *Eur J Clin Pharmacol.*, Nov;69(11):1933-8.

Cho 2024 CK Cho, JY Mo, E Ko, P Kang, CG Jang, SY Lee, YJ Lee, JW Bae, CI Choi (2024). Physiologically based pharmacokinetic (PBPK) modeling of pitavastatin in relation to SLCO1B1 genetic polymorphism. *Arch Pharm Res.*, Feb;47(2):95-110.

Duan 2017 P Duan, P Zhao, L Zhang (2017). Physiologically Based Pharmacokinetic (PBPK) Modeling of Pitavastatin and Atorvastatin to Predict Drug-Drug Interactions (DDIs). *Eur J Drug Metab Pharmacokinet.* Aug;42(4):689-705.

Ieiri 2007 Ieiri I, Suwannakul S, Maeda K, Uchimaru H, Hashimoto K, Kimura M, Fujino H, Hirano M, Kusuhara H, Irie S, Higuchi S, Sugiyama Y. SLCO1B1 (OATP1B1, an uptake transporter) and ABCG2 (BCRP, an efflux transporter) variant alleles and pharmacokinetics of pitavastatin in healthy volunteers. Clin *Pharmacol Ther.* 2007 Nov;82(5):541-7

Kimoto 2022 E Kimoto, C Costales, MA West, YA Bi, M Vourvahis, A David Rodrigues, MVS Varma (2022). Biomarker-Informed Model-Based Risk Assessment of Organic Anion Transporting Polypeptide 1B

Mediated Drug-Drug Interactions. *Clin Pharmacol Ther*, Feb;111(2):404-415.

Lou 2015 Z Luo, Y Zhang, J Gu, P Feng, Y Wang (2015). Pharmacokinetic Properties of Single- and Multiple-Dose Pitavastatin Calcium Tablets in Healthy Chinese Volunteers. *Curr Ther Res Clin Exp*, Mar 3;77:52-7.

Meyer 2012 Meyer M, Schneckener S, Ludewig B, Kuepfer L, Lippert J. (2012). Using expression data for quantification of active processes in physiologically based pharmacokinetic modeling. *Drug Metab Dispos.* May;40(5), 892-901.

Mochizuki 2022 T Mochizuki, Y Aoki, T Yoshikado, K Yoshida, Y Lai, H Hirabayashi, Y Yamaura, K Rockich, K Taskar, T Takashima, X Chu, MJ Zamek-Gliszczynski, J Mao, K Maeda, K Furihata, Y Sugiyama, H Kusuhara (2022). Physiologically-based pharmacokinetic model-based translation of OATP1B-mediated drug-drug interactions from coproporphyrin I to probe drugs. *Clin Transl Sci.* Jun;15(6):1519-1531.

Mori 2020 D Mori, E Kimoto, B Rago, Y Kondo, A King-Ahmad, R Ramanathan, LS Wood, JG Johnson, VH Le, M Vourvahis, A David Rodrigues (2020). Dose-dependent inhibition of OATP1B by rifampicin in healthy volunteers: comprehensive evaluation of candidate biomarkers and OATP1B probe drugs. *Clinical Pharmacology & Therapeutics*, Apr;107(4):1004-13.

Nishimura 2003 Nishimura M, Yaguti H, Yoshitsugu H, Naito S, Satoh T. (2003). Tissue distribution of mRNA expression of human cytochrome P450 isoforms assessed by high-sensitivity real-time reverse transcription PCR. *Yakugaku Zasshi.* May;123(5), 369-75.

Prasad 2013 Prasad B, Lai Y, Lin Y, Unadkat JD (2013) Interindividual variability in the hepatic expression of the human breast cancer resistance protein (BCRP/ABCG2): effect of age, sex, and genotype. Journal of pharmaceutical sciences 102(3):787–93

Prasad 2014 Prasad B, Evers R, Gupta A, Hop CECA, Salphati L, Shukla S, Ambudkar SV, Unadkat JD (2014) Interindividual variability in hepatic organic anion-transporting polypeptides and PGlycoprotein (ABCB1) protein expression: Quantification by liquid chromatography tandem mass spectroscopy and influence of genotype, age, and sex. Drug Metabolism and Disposition 42(1):78–88

Prueksaritanont 2014T Prueksaritanont, X Chu, R Evers, SO Klopfer, L Caro, PA Kothare, C Dempsey, S Rasmussen, R Houle, G Chan, X Cai, R Valesky, IP Fraser, SA Stoch (2014). Pitavastatin is a more sensitive and selective organic anion-transporting polypeptide 1B clinical probe than rosuvastatin. Br J Clin Pharmacol., Sep;78(3):587-98.

Takehara 2018 I Takehara, T Yoshikado, K Ishigame, D Mori, KI Furihata, N Watanabe, O Ando, K Maeda, Y Sugiyama, H Kusuhara (2018). Comparative study of the dose-dependence of OATP1B inhibition by rifampicin using probe drugs and endogenous substrates in healthy volunteers. *Pharmaceutical research*, Jul;35:1-3.
