# Supplementary material for: Coproporphyrin I PBPK model in the open systems pharmacology suite enables precise prediction of OATP 1B- mediated drug interactions through calibrated In vivo inhibition constants
Source: Front Pharmacol. 2026 Jul 28;17:1828033. doi: 10.3389/fphar.2026.1828033 (PMC13457544; doi:10.3389/fphar.2026.1828033)
Supplement: Supplementary file 3 [file DataSheet3.pdf]

Building and Evaluation of a PBPK Model for Atorvastatin Acid in Healthy Adults

**Version**	**x.x-OSPy.y**
based on *Model Snapshot* and *Evaluation Plan*	https://github.com/Open-Systems-Pharmacology/Atorvastatin-Model/releases/tag/vx.x
OSP Version	y.y
Qualification Framework Version	z.z

This evaluation report and the corresponding PK-Sim® project file are filed at:

https://github.com/Open-Systems-Pharmacology/OSP-PBPK-Model-Library/

Table of Contents

1 Introduction

Atorvastatin, a 3-hydroxy-3-methylglutaryl-coenzyme A (HMG-CoA) reductase inhibitor, is a widely prescribed statin to treat hypercholesterolemia (Morse 2019). The recommended dosage for adults ranges from 10 to 80 mg once daily (Pfizer 2019). Following oral administration, atorvastatin acid is rapidly absorbed from the gastrointestinal tract, reaching maximum plasma concentrations within one to two hours (Pfizer 2019). However, its bioavailability is notably low (~14%) due to extensive first pass metabolism by cytochrome P450 (CYP) 3A4 (Pfizer 2019). Atorvastatin acid is also a substrate of multiple transporters, including the influx transporters organic anion transporting polypeptides (OATP) 1B1 and OATP1B3 (Vildhede 2014), as well as the efflux transporters P-glycoprotein (P-gp) and breast cancer resistant protein (BCRP) (Deng 2021), which makes atorvastatin acid highly susceptible to drug–drug interactions (DDIs) mediated by both enzymes and transporters.

This whole-body atorvastatin acid PBPK model is intended to be used as a victim drug in CYP3A4- and OATP1B1/1B3-mediated DDIs. It has been developed using published pharmacokinetic clinical data by Backman 2005 (Backman 2005), Bullman 2011 (Bullman 2011), Di Spirito 2008 (Di Spirito 2008), Gandelman 2011 (Gandelman 2011), Kantola 1998 (Kantola 1998), Lau 2007 (Lau 2007), Mazzu 2000 (Mazzu 2000), Mori 2020 (Mori 2020), Park 2022 Park 2022, Patiño-Rodríguez 2015 (Patiño-Rodríguez 2015), Shin 2011 (Shin 2011), Siedlik 1999 (Siedlik 1999), Takehara 2018 (Takehara 2018), Teng 2013 (Teng 2013) and Whitfield 2010 (Whitfield 2010) after oral administration. The selected clinical study data captures a broad dosing range of 1.0 to 80.0 mg atorvastatin acid.

The model has then been evaluated by simulating clinical studies and comparing with respective observed data.

The presented model includes the following features:

- Metabolism by CYP3A4,
- Metabolism by CYP3A5 (only implemented in Park 2022 for CYP3A5 normal metabolizers (Park 2022); otherwise, the kcat has been set to 0),
- Transport by BCRP,
- Transport by P-gp, and
- Transport by OATP1B1/3.

2 Methods

2.1 Modeling strategy

The general concept of building a PBPK model has previously been described by Kuepfer et al. (Kuepfer 2016). Relevant information on anthropometric (height, weight) and physiological parameters (e.g. blood flows, organ volumes, binding protein concentrations, hematocrit, cardiac output) in adults was gathered from the literature and has been previously published (Willmann 2007). The information was incorporated into PK-Sim® and was used as default values for the simulations in adults.

The applied activity and variability of plasma proteins and active processes that are integrated into PK-Sim® are described in the publicly available PK-Sim® Ontogeny Database Version (PK-Sim Ontogeny Database Version 7.3) or otherwise referenced for the specific process.

A PBPK model was built based on seven mean plasma concentration-time profiles after oral administration of atorvastatin acid obtained from the clinical studies by Backman 2005 (Backman 2005), Bullman 2011 (Bullman 2011), Gandelman 2011 (Gandelman 2011), Kantola 1998 (Kantola 1998) and Teng 2013 (Teng 2013). To inform CYP3A4- and OATP1B1/1B3-mediated pathways, the DDI studies with itraconazole (Kantola 1998) and rifampicin (Lau 2007) were included in the model building. Virtual mean individuals were generated for each study based on the reported mean and mode demographic information (including sex, ethnicity, body weight, height, body mass index, and age). If demographic information was missing, provided mean values in PK-Sim®, that were computed from the respective population database, were applied. The relative tissue-specific expressions of the enzyme and transporter predominantly being involved in the metabolism/transport of atorvastatin acid (CYP3A4, CYP3A5, BCRP, OATP1B1/1B3, and P-gp) were considered (Nishimura 2003, Nishimura 2005, Kolesnikov 2015).

A specific set of parameters (see below) was optimized to describe the disposition of atorvastatin acid using the Parameter Identification module provided in PK-Sim®.

The model was then verified by simulating further clinical studies reporting pharmacokinetic concentration-time profiles after oral administration of atorvastatin acid.

Details about input data (physicochemical, *in vitro* and clinical) can be found in Section 2.2.

Details about the structural model and its parameters can be found in Section 2.3.

2.2 Data used

2.2.1 2.2.1 In vitro and physicochemical data

A literature search was performed to collect available information on physicochemical properties of atorvastatin acid. The obtained information from the literature is summarized in the table below, and is used for model building.

Parameter	Unit	Value	Source	Description
MW	g/mol	558.66	Zhang 2015	Molecular weight
pK_a (acid)		4.46	Zhang 2015	Acid dissociation constant of conjugate acid
Solubility (pH=6)	g/L	1.22	Morse 2019	Solubility
logP		4.07	Duan 2017	Partition coefficient between octanol and water
fu	%	5.10	Zhang 2015	Fraction unbound in plasma
K_m CYP3A4	µmol/L	25.60	Jacobsen 2000	CYP3A4 Michaelis-Menten constant
K_m CYP3A5	µmol/L	42.60	Park 2008	CYP3A5 Michaelis-Menten constant
K_m BCRP	µmol/L	82.41	Deng 2021	BCRP Michaelis-Menten constant
K_m OATP1B1/3	µmol/L	0.77	Vildhede 2014	OATP1B1/3 Michaelis-Menten constant
K_m P-gp	µmol/L	10.7	Deng 2021	P-gp Michaelis-Menten constant
Formulation		Solution		Formulation used in simulations

2.2.2 2.2.2 Clinical data

A literature search was performed to collect available clinical data on atorvastatin acid in adults.

The following publications were found in adults for model building:

Publication	Arm / Treatment / Information used for model building
Gandelman 2011	Plasma PK profile in healthy subjects after single oral administration of 10 mg atorvastatin acid (study A[a])
Bullman 2011	Plasma PK profile in healthy subjects after multiple oral administrations of 40 mg atorvastatin acid (once daily for 7 days, control of DDI with phenytoin)
Backman 2005	Plasma PK profile in healthy subjects after single oral administration of 20 mg atorvastatin acid (control of gemfibrozil DDI)
Kantola 1998	Plasma PK profile in healthy subjects after single oral administration of 40 mg atorvastatin acid (control of itraconazole DDI)
Kantola 1998	Plasma PK profile in healthy subjects after multiple oral administrations of 200 mg

Publication	Arm / Treatment / Information used for model building
	itraconazole (once daily for 4 days) and on day 4 together with a single oral administration of 40 mg atorvastatin acid (itraconazole DDI)
Lau 2007	Plasma PK profile in healthy subjects after single oral administration of 40 mg atorvastatin acid, preceded by 30 minutes infusion of 600 mg rifampicin (rifampicin DDI)
Teng 2013	Plasma PK profile in healthy subjects after single oral administration of 80 mg atorvastatin acid

The following dosing scenarios were simulated and compared to respective data for model verification:

Scenario	Data reference
single oral administration of 1 mg atorvastatin acid (control of rifampicin DDI)	Mori 2020
single oral administration of 1 mg atorvastatin acid (control of rifampicin DDI)	Takehara 2018
single oral administration of 10 mg atorvastatin acid (study C[a])	Gandelman 2011
single oral administration of 10 mg atorvastatin acid (control of erythromycin DDI)	Siedlik 1999
single oral administration of 20 mg atorvastatin acid (control of itraconazole DDI)	Mazzu 2020
single oral administration of 20 mg atorvastatin acid	Patiño-Rodríguez 2015
single oral administration of 20 mg atorvastatin acid (control of clarithromycin DDI in the CYP3A5 expressor group)	Shin 2011
single oral administration of 20 mg atorvastatin acid (control of clarithromycin DDI in the CYP3A5 non-expressor group)	Shin 2011
single oral administration of 40 mg atorvastatin acid (control of rifampicin DDI)	Backman 2005
single oral administration of 40 mg atorvastatin acid (control of gemfibrozil DDI)	Whitfield 2011
multiple oral administrations of 40 mg atorvastatin acid (once daily for 7 days)	Bullman 2011
single oral administration of 40 mg atorvastatin acid (control of rifampicin DDI)	Lau 2007
multiple oral administrations of 80 mg atorvastatin acid (once daily for 14 days)	Di Spirito 2008
single oral administration of 80 mg atorvastatin acid (study B[a])	Gandelman 2011
single oral administration of 80 mg atorvastatin acid (study D[a])	Gandelman 2011
single oral administration of 80 mg atorvastatin acid (CYP3A5 normal metabolizer)	Park 2022
single oral administration of 80 mg atorvastatin acid (CYP3A5 intermediate metabolizer)	Park 2022
single oral administration of 80 mg atorvastatin acid (CYP3A5 poor metabolizer)	Park 2022

[a] In the study by Gandelman 2011, a small tablet formulation (1x10 mg in study A and 1x80 mg in study B) and a chewable tablet formulation (1x10 mg in study C and 1x80 mg in study D) was tested in two single-dose bioequivalent studies (10 mg and 80 mg). Only the plasma profiles of the reference product in each study were digitized.

2.3 Model parameters and assumptions

2.3.1 2.3.1 Absorption

A parameter value for `Specific intestinal permeability` and a measured solubility at pH=6 sourced from the literature were incorporated into the model (see Section 2.2.1). Additionally, a solution formulation was selected (see Section 2.2.1).

2.3.2 2.3.2 Distribution

Atorvastatin acid is highly bound to plasma proteins (approx. 94.9%) (see Section 2.2.1). A value of 5.10% was used in the PBPK model for `Fraction unbound (plasma, reference value)`. The major binding partner was set to albumin (see Section 2.2.1).

An important parameter influencing the resulting volume of distribution is lipophilicity. The reported literature logP value of 4.07 was used in the model (see Section 2.2.1).

After testing the available organ-plasma partition coefficient and cell permeability calculation methods built in PK-Sim®, observed clinical data was best described by choosing the partition coefficient calculation by `Schmitt` and cellular permeability calculation by `Charge dependent Schmitt`.

2.3.3 2.3.3 Metabolism and Elimination

Two metabolic pathways were implemented into the model via Michaelis-Menten kinetics:

- CYP3A4

The CYP3A4 expression profile is based on high-sensitive real-time RT-PCR (Nishimura 2003). Metabolic enzyme activity was described as saturable process following Michaelis-Menten kinetics, where the `Km` was taken from the literature and the `kcat` was optimized based on clinical data (see Section 2.3.4).

- CYP3A5

The CYP3A5 metabolic pathway was implemented only for normal metabolizers in the study by Park 2022; for all other studies, CYP3A5 `kcat` value was set to 0. The CYP3A5 expression profile is based on high-sensitive real-time RT-PCR (Nishimura 2003). Metabolic enzyme activity was described as saturable

process following Michaelis-Menten kinetics, where the `Km` was taken from the literature and the `kcat` was optimized based on clinical data (see Section 2.3.4).

In addition, several transport proteins were implemented into the model via Michaelis-Menten kinetics:

- BCRP

The BCRP expression profile is based on whole genome expression arrays from ArrayExpress (Kolesnikov 2015). Transporter activity was described as saturable process following Michaelis-Menten kinetics, where the `Km` was taken from the literature and `kcat` was optimized based on clinical data (see Section 2.3.4).

- OATP1B1/3

For OATP1B1/3 the expression profile was considered only for OATP1B1 and is based on high-sensitive real-time RT-PCR (Nishimura 2005). Transporter activity was described as saturable process following Michaelis-Menten kinetics, where the `Km` was taken from the literature and `kcat` was optimized based on clinical data (see Section 2.3.4).

- P-gp

The P-gp expression profiles is based on high-sensitive real-time RT-PCR (Nishimura 2005) with an intestinal mucosa of factor 3.57 (Hanke 2018). Transporter activity was described as saturable process following Michaelis-Menten kinetics, where the Km was taken from the literature and kcat was optimized based on clinical data (see Section 2.3.4).

Additionally, fraction of bile that was continously released was set to 1 (`EHC continuous fraction`).

2.3.4 2.3.4 Automated Parameter Identification

This is the result of the final parameter identification:

Model Parameter	Optimized Value	Unit
`kcat` (CYP3A4)	8.57	1/min
`kcat` (CYP3A5) NM	1350.42	1/min
`kcat` (CYP3A5) PM/IM	0	1/min
`kcat` (BCRP)	932.28	1/min
`kcat` (OATP1B1/3)	1970.16	1/min
`kcat` (P-gp)	614.28	1/min

3 Results and Discussion

The PBPK model for atorvastatin acid was developed and evaluated using publicly available clinical pharmacokinetic data from studies listed in Section 2.2.2.

The next sections show:

1. The final model input parameters for the building blocks: Section 3.1.
2. The overall goodness of fit: Section 3.2.
3. Simulated vs. observed concentration-time profiles for the clinical studies used for model building and for model verification: Section 3.3.

3.1 Atorvastatin acid final input parameters

The compound parameter values of the final PBPK model are illustrated below.

3.1.1 Compound: Atorvastatin

3.1.1.1 Parameters

Name	Value	Value Origin	Alternative	Default
Solubility at reference pH	1.22 mg/ml	Publication-In Vitro-Morse et al. 2019	Morse 2019 (a)	True
Reference pH	6	Publication-In Vitro-Morse et al. 2019	Morse 2019 (a)	True
Lipophilicity	4.07 Log Units	Publication-Duan et al. 2016	Lit.	True
Fraction unbound (plasma, reference value)	5.1 %	Publication-In Vitro-Zhang et al. 2015	Measurement	True
Specific intestinal permeability (transcellular)	0.000449 cm/s	Publication-Other-Morse et al. 2019	Literature	True
F	1	Publication-Zhang et al. 2015		
Is small molecule	Yes			
Molecular weight	558.66 g/mol	Publication-Zhang et al. 2015		
Plasma protein binding partner	Albumin			

3.1.1.2 Calculation methods

Name	Value
Partition coefficients	Schmitt
Cellular permeabilities	Charge dependent Schmitt

3.1.1.3 Processes

3.1.1.3.1 Metabolizing Enzyme: CYP3A4-Jacobsen 2000

Molecule: CYP3A4

3.1.1.3.1.1 Parameters

Name	Value	Value Origin
In vitro Vmax/recombinant enzyme	29.8 pmol/min/pmol rec. enzyme	Publication-In Vitro-Jacobsen et al. 2000
Km	25.6 µM	Publication-In Vitro-Jacobsen et al. 2000
kcat	8.5656178266 1/min	Parameter Identification-Parameter Identification

3.1.1.3.2 Transport Protein: OATP1B1/1B3-Vildhede 2014

Molecule: OATP1B1/1B3

3.1.1.3.2.1 Parameters

Name	Value	Value Origin
In vitro Vmax/transporter	0.284913793 pmol/min/pmol transporter	Publication-In Vitro-Vildhede et al. 2014
Km	0.77 µmol/l	Publication-In Vitro-Vildhede et al. 2014
kcat	1970.1599932122 1/min	Parameter Identification-Parameter Identification

3.1.1.3.3 Transport Protein: P-gp-Deng et al. 2021

Molecule: P-gp

3.1.1.3.3.1 Parameters

Name	Value	Value Origin
In vitro Vmax/transporter	0.609259259 pmol/min/pmol transporter	Publication-Deng et al. 2021

Name	Value	Value Origin
Km	10.7 µmol/l	Publication-Deng et al. 2021
kcat	614.2798089947 1/min	Parameter Identification-Parameter Identification

3.1.1.3.4 Transport Protein: BCRP-Deng et al. 2021

Molecule: BCRP

3.1.1.3.4.1 Parameters

Name	Value	Value Origin
In vitro Vmax/transporter	0.924657534 pmol/min/pmol transporter	Publication-Deng et al. 2021
Km	82.41 µmol/l	Publication-Deng et al. 2021
kcat	932.2770971151 1/min	Parameter Identification-Parameter Identification

3.1.1.3.5 Metabolizing Enzyme: CYP3A5-CYP3A5 PM/IM

Molecule: CYP3A5

3.1.1.3.5.1 Parameters

Name	Value	Value Origin
In vitro Vmax/recombinant enzyme	10.4 pmol/min/pmol rec. enzyme	Publication-Park et al. 2008
Km	42.6 µmol/l	Publication-Park et al. 2008
kcat	0 1/min	Unknown-assumed

3.1.1.3.6 Metabolizing Enzyme: CYP3A5-CYP3A5 NM

Molecule: CYP3A5

3.1.1.3.6.1 Parameters

Name	Value	Value Origin
In vitro Vmax/recombinant enzyme	10.4 pmol/min/pmol rec. enzyme	Publication-Park et al. 2008
Km	42.6 µmol/l	Publication-Park et al. 2008
kcat	1350.4213435857 1/min	Parameter Identification-Parameter Identification

3.2 Atorvastatin acid Diagnostics Plots

Below you find the goodness-of-fit visual diagnostic plots for the PBPK model performance of all data used presented in Section 2.2.2.

The first plot shows observed versus simulated plasma concentration, the second weighted residuals versus time.

Table 3-1: GMFE for Goodness of fit plot for plasma concentrations.

Group	GMFE
Atorvastatin acid (model building)	1.37
Atorvastatin acid (model verification)	1.79
All	1.70

Figure 3-1: Goodness of fit plot for plasma concentrations.

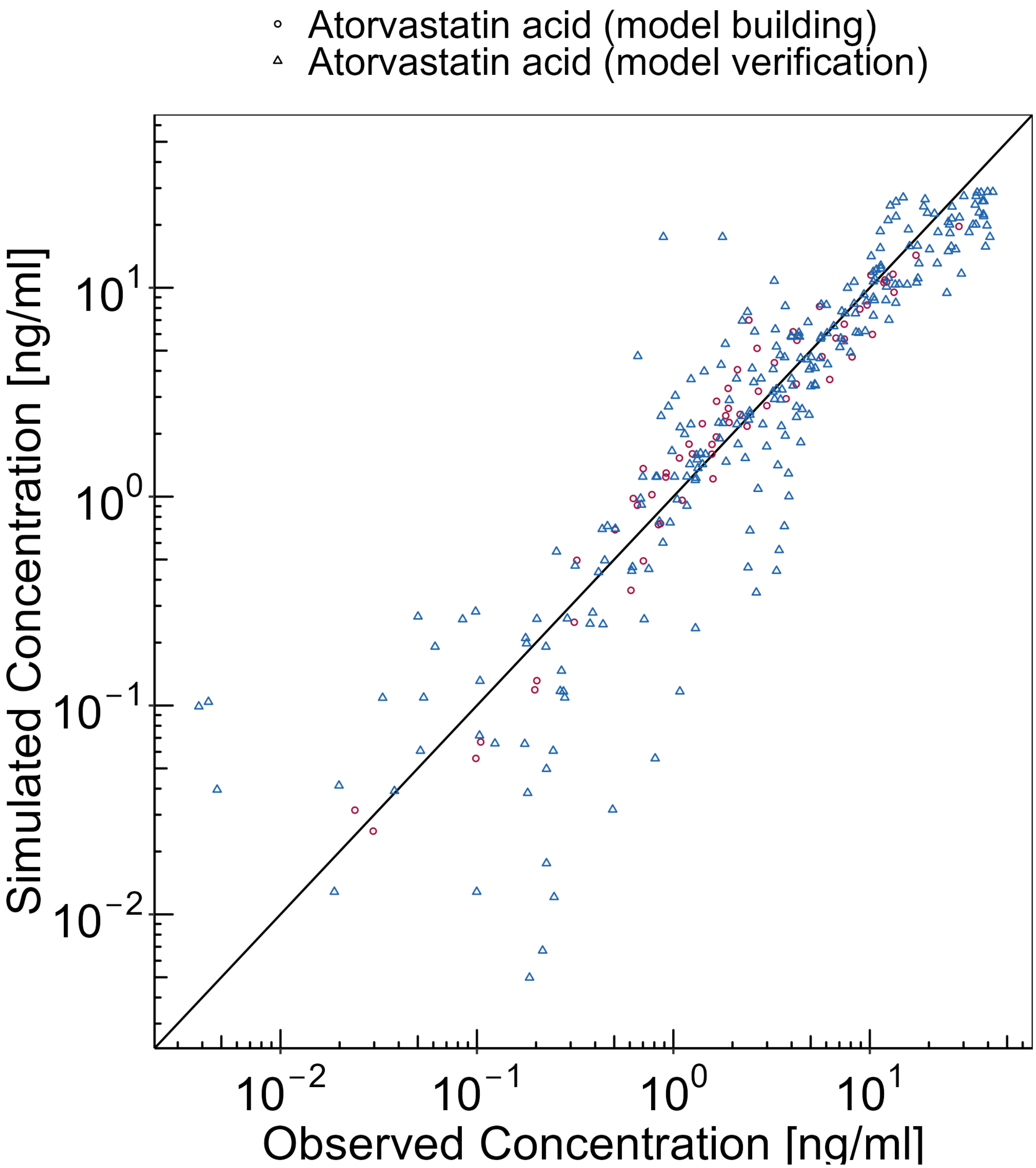

Figure 3-2: Goodness of fit plot for plasma concentrations.

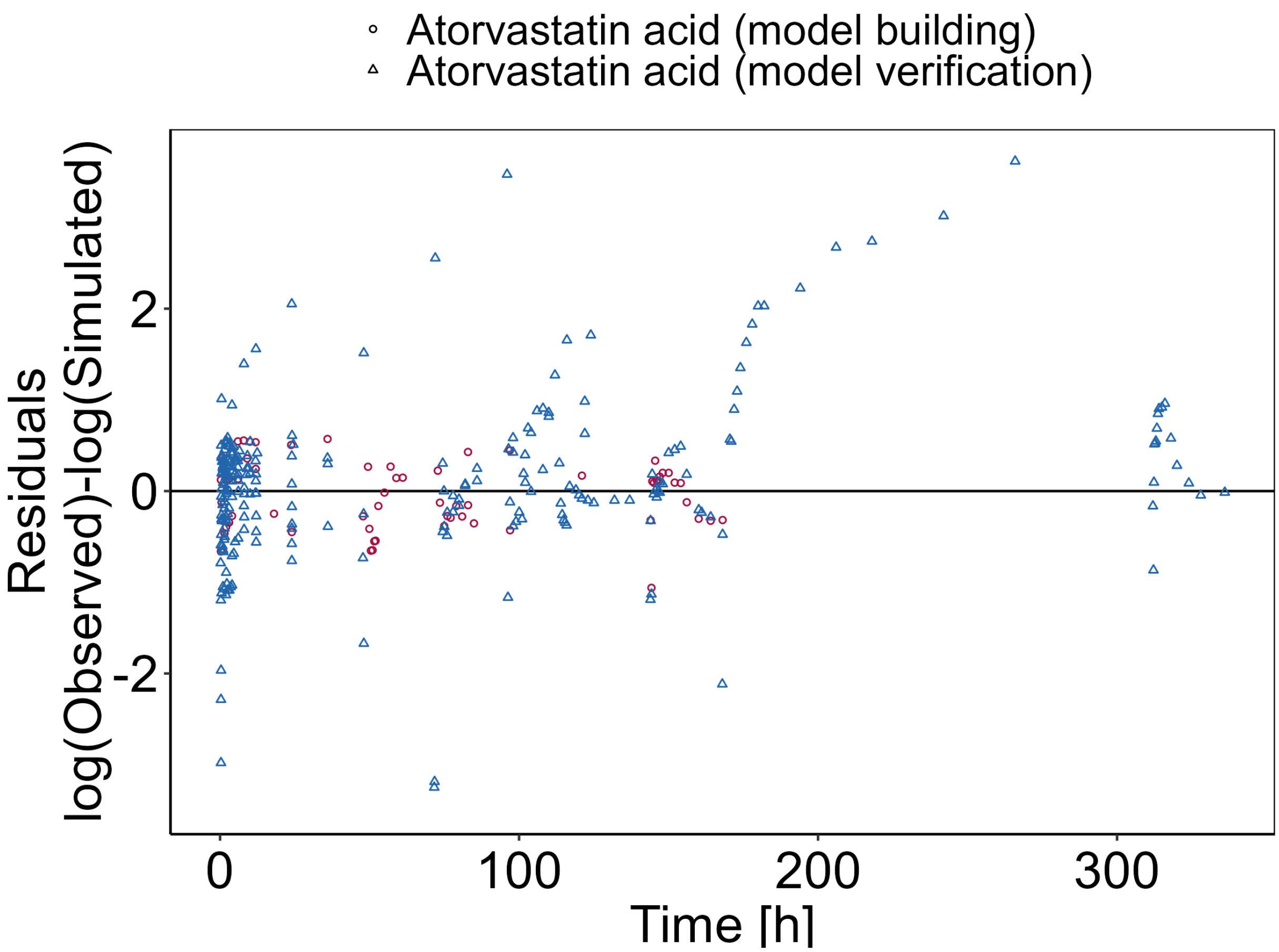

3.3 Concentration-Time Profiles

Simulated versus observed concentration-time profiles of all data listed in Section 2.2.2 are presented below.

3.3.1 Model Building

Figure 3-3: Time Profile Analysis

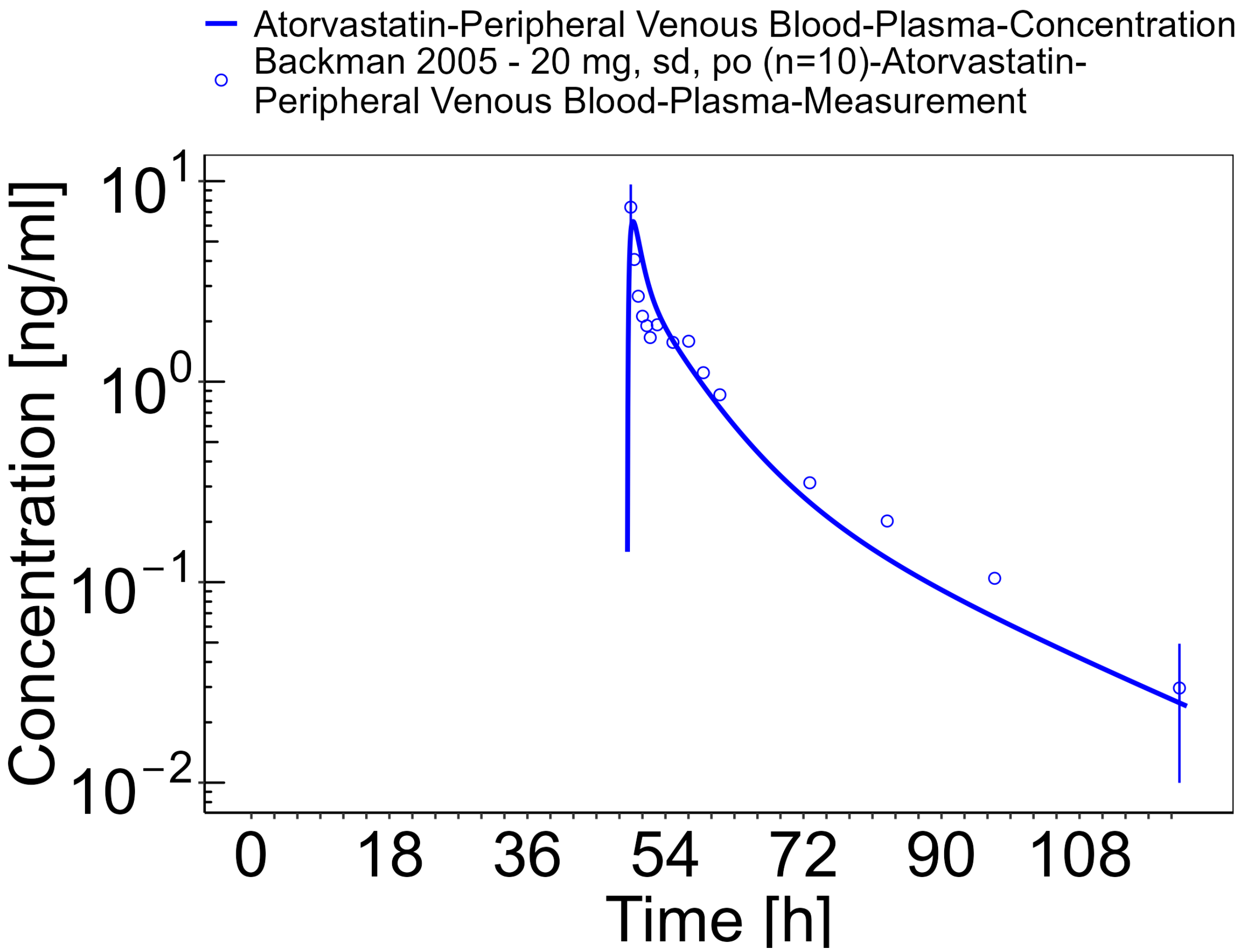

Figure 3-4: Time Profile Analysis

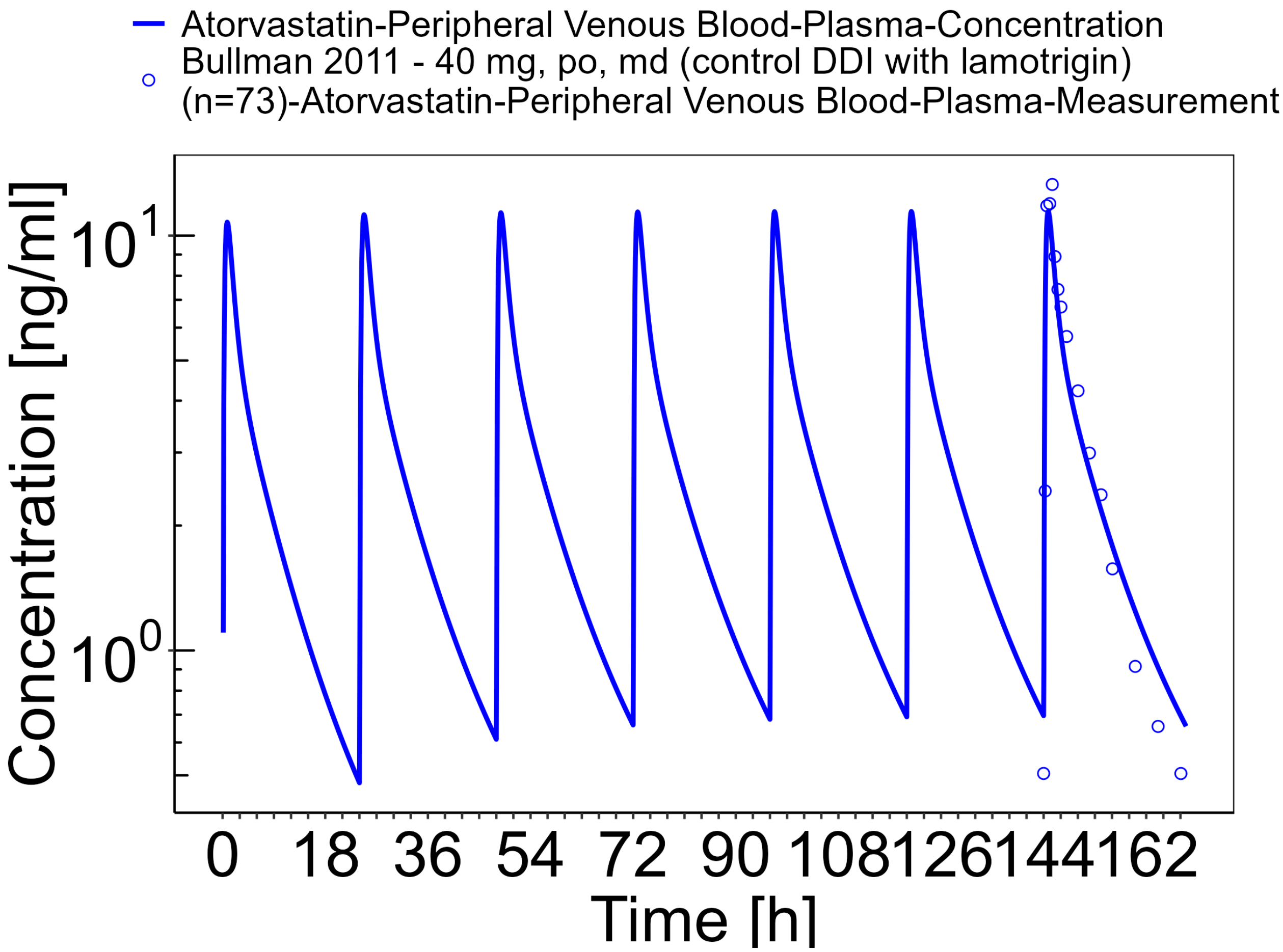

Figure 3-5: Time Profile Analysis

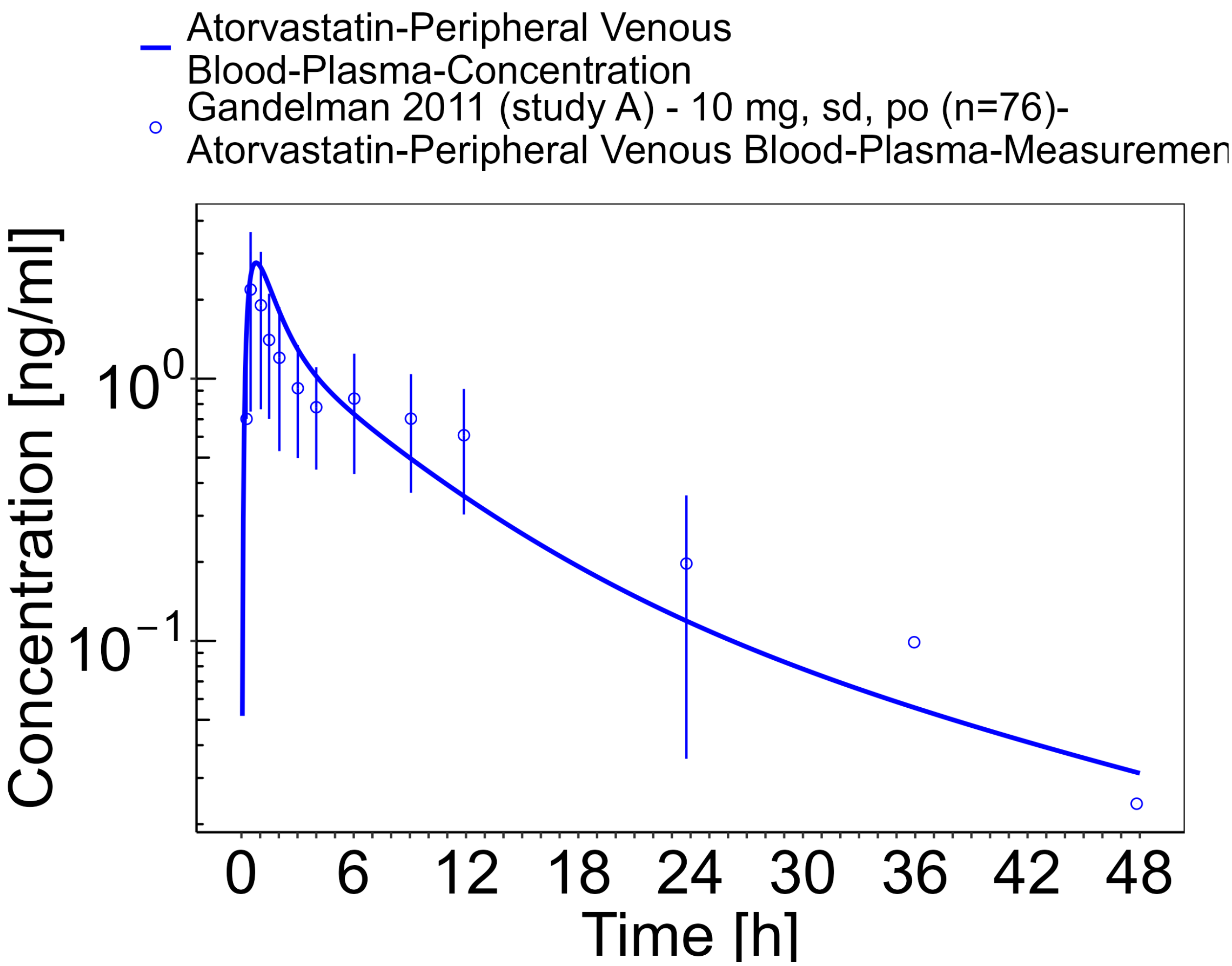

Figure 3-6: Time Profile Analysis

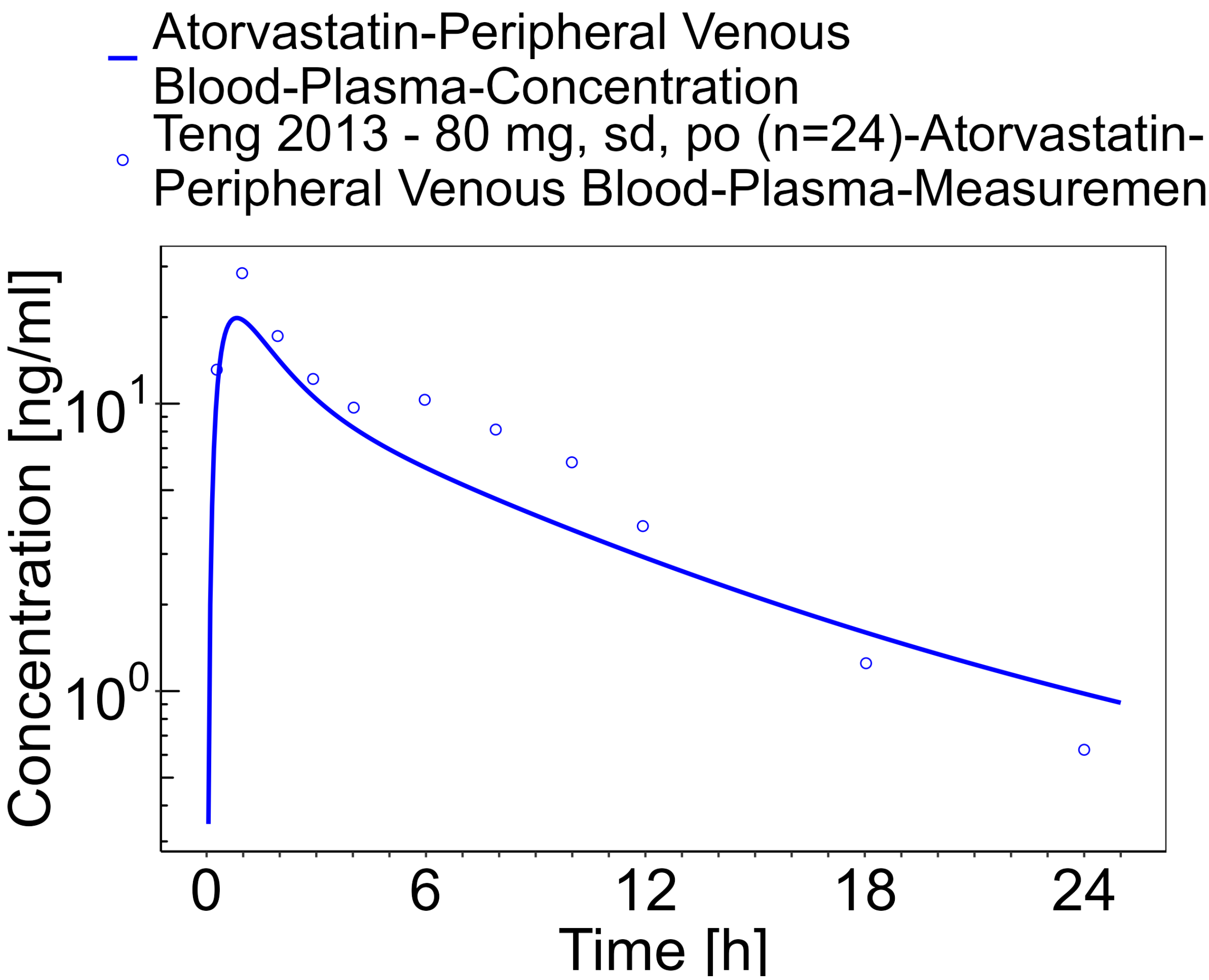

Figure 3-7: Time Profile Analysis

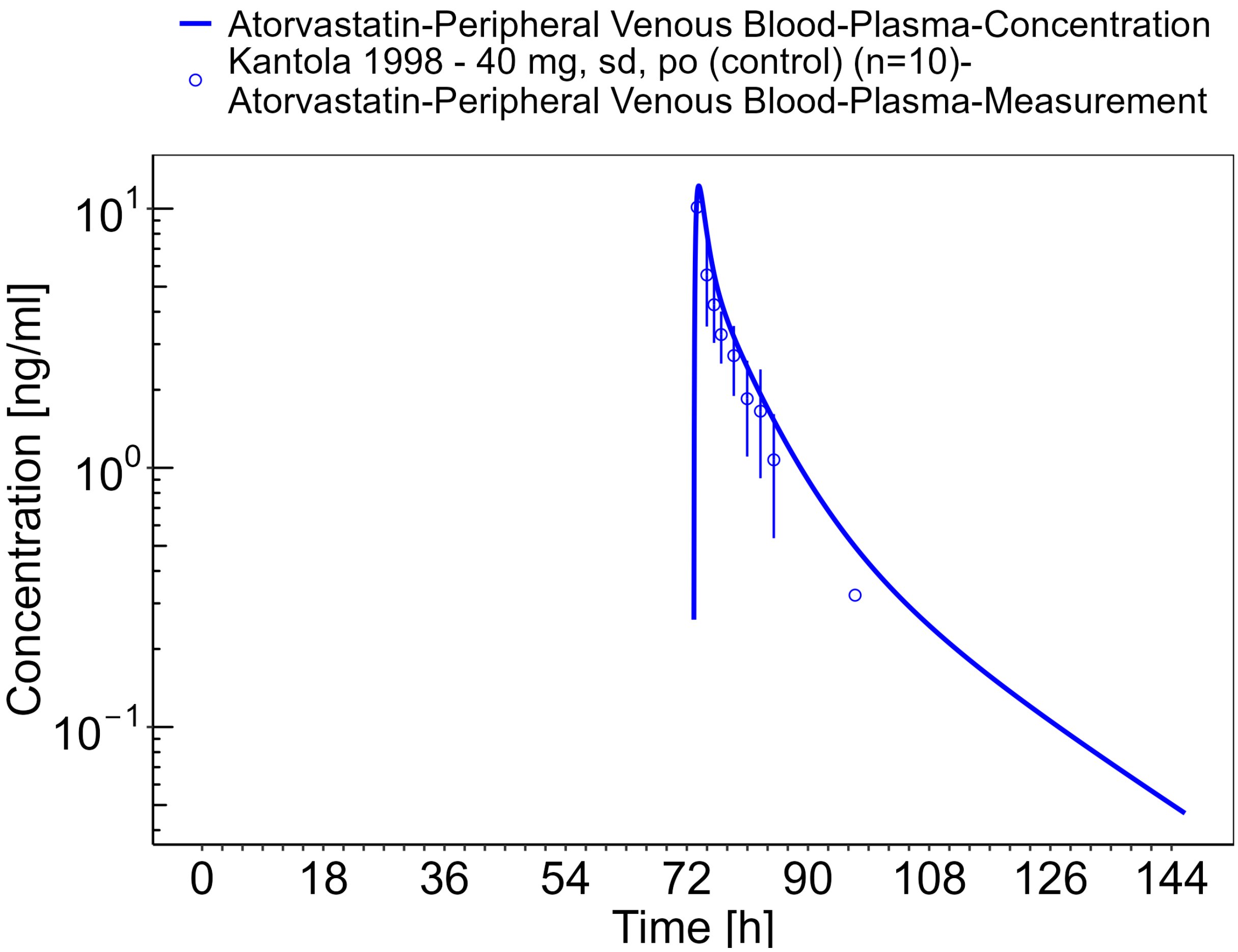

3.3.2 Model Verification

Figure 3-8: Time Profile Analysis

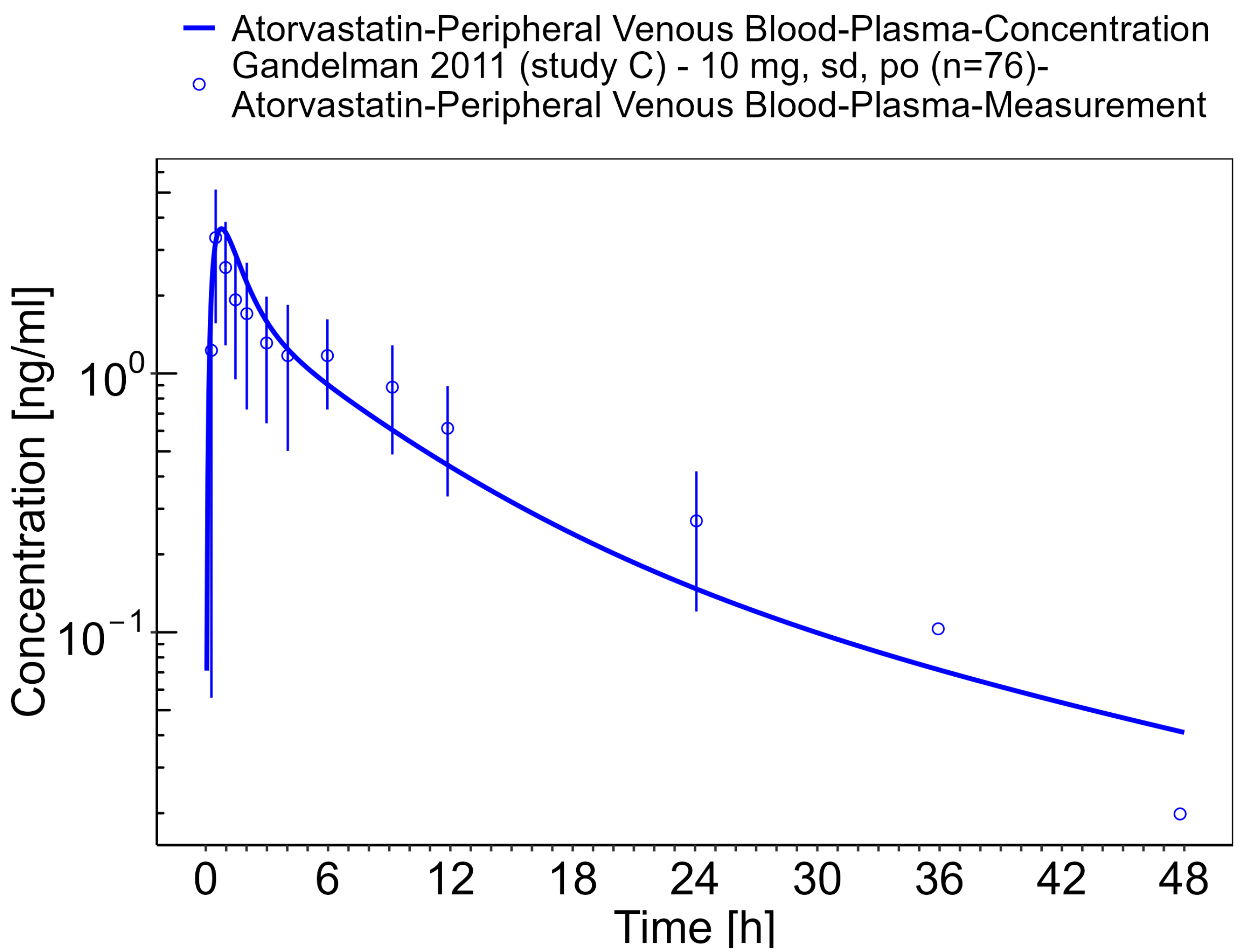

Figure 3-9: Time Profile Analysis

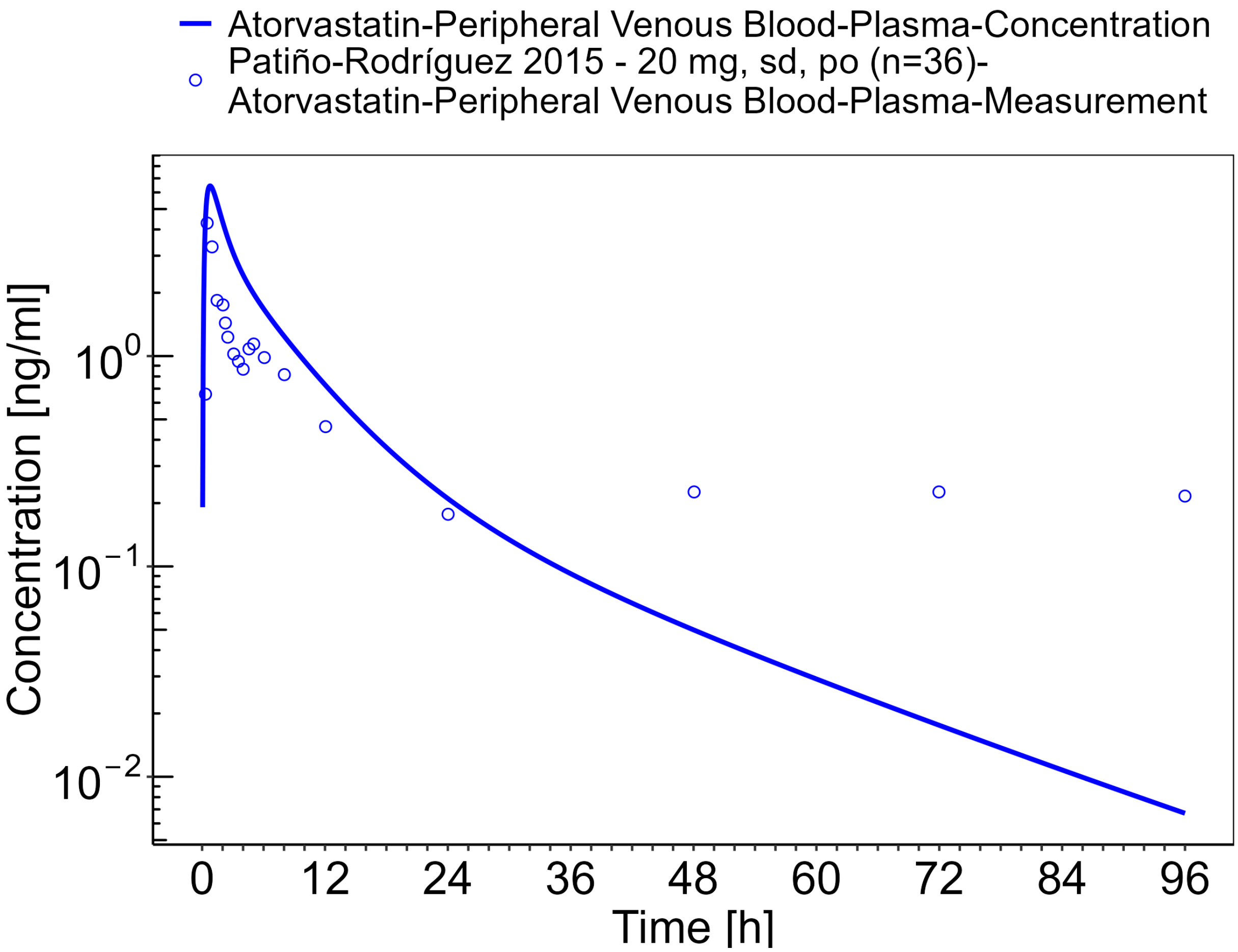

Figure 3-10: Time Profile Analysis

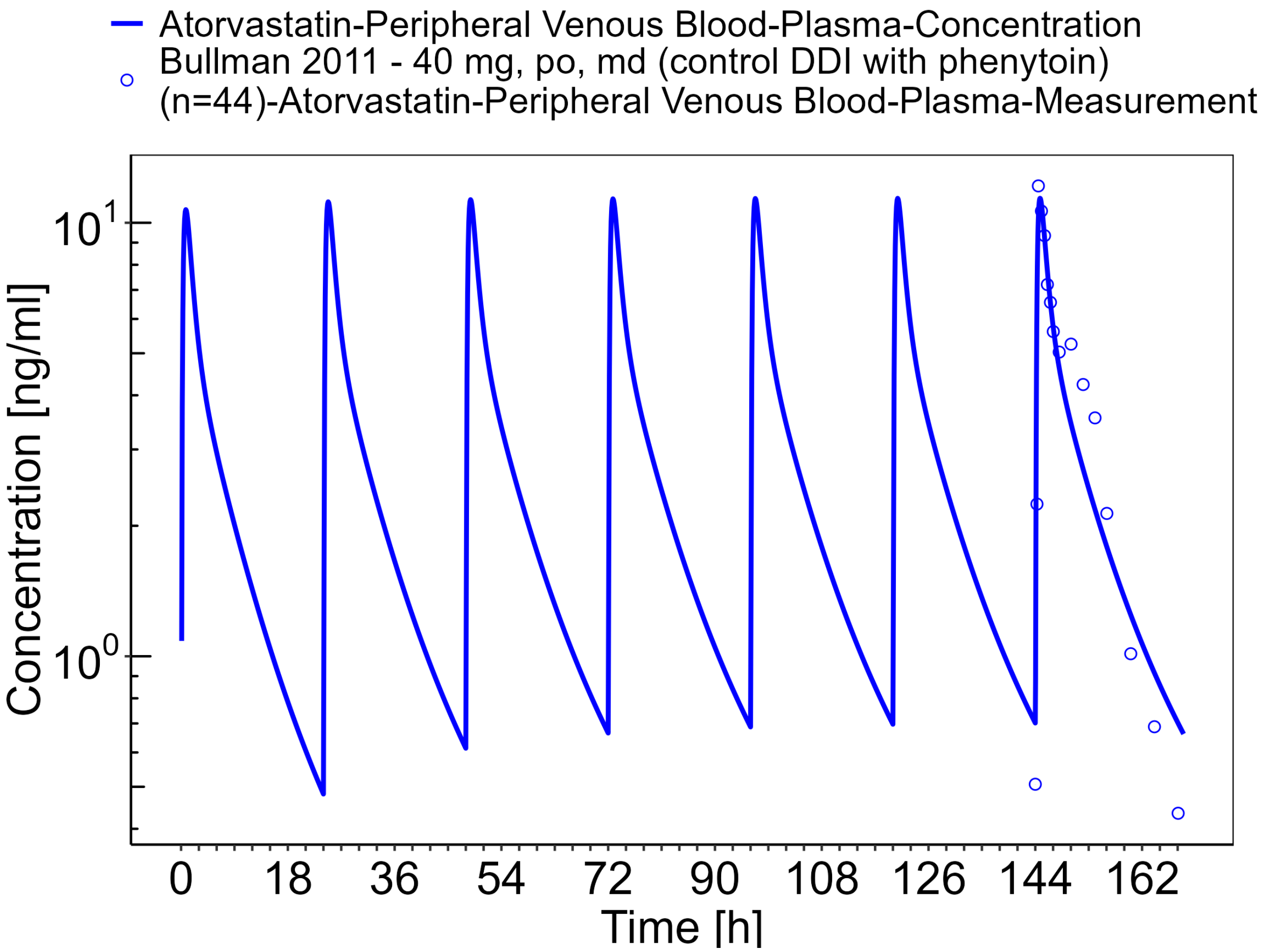

Figure 3-11: Time Profile Analysis

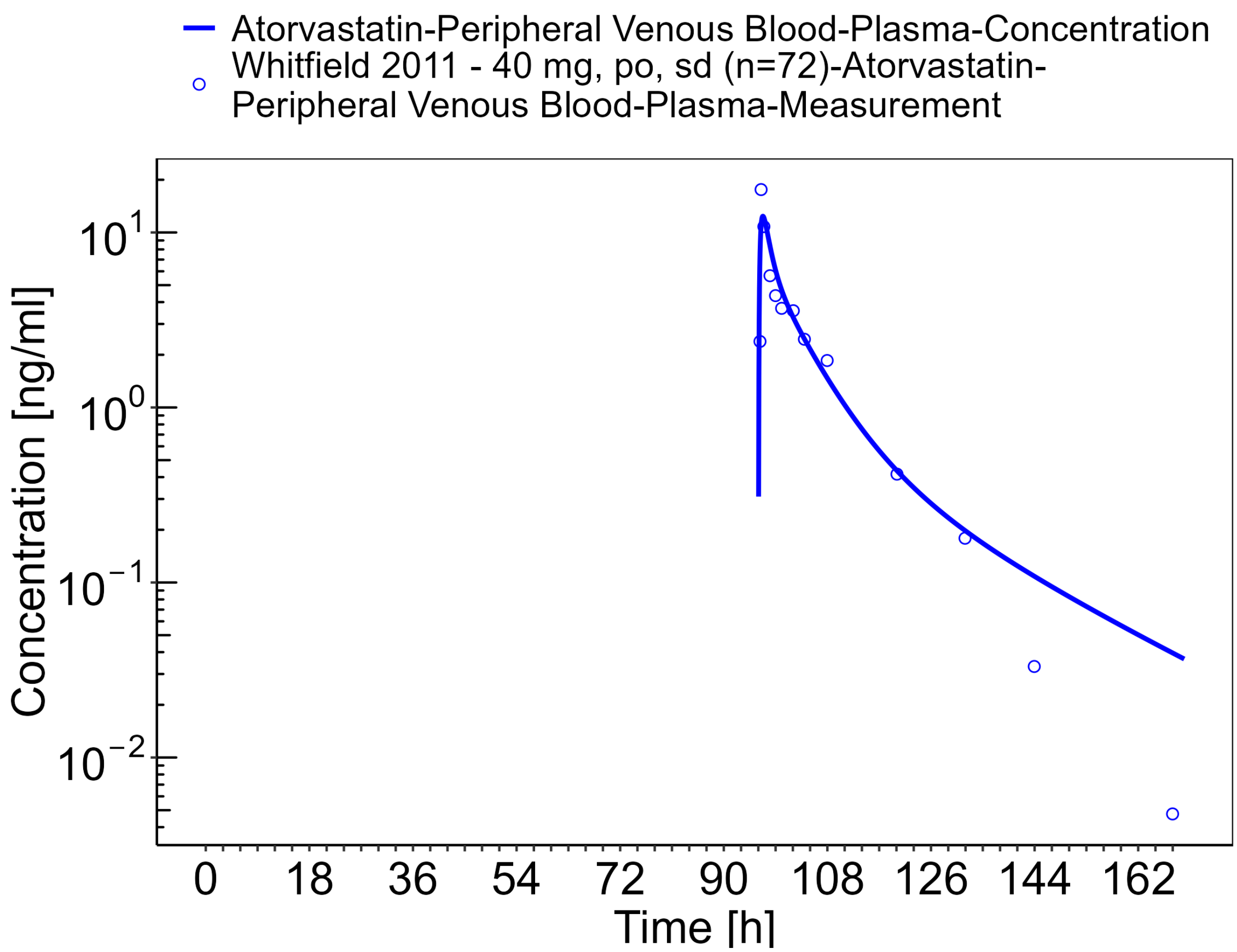

Figure 3-12: Time Profile Analysis

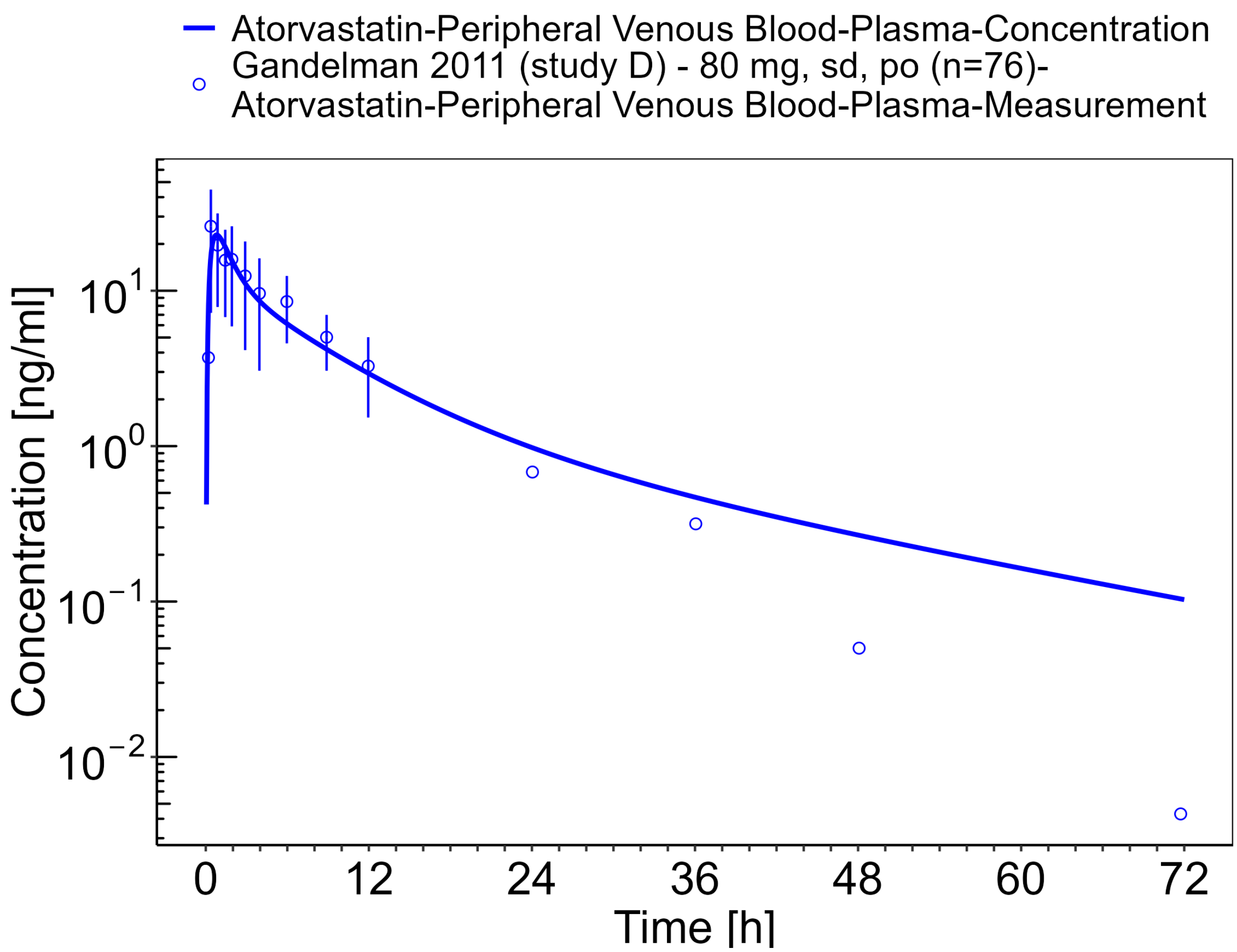

Figure 3-13: Time Profile Analysis

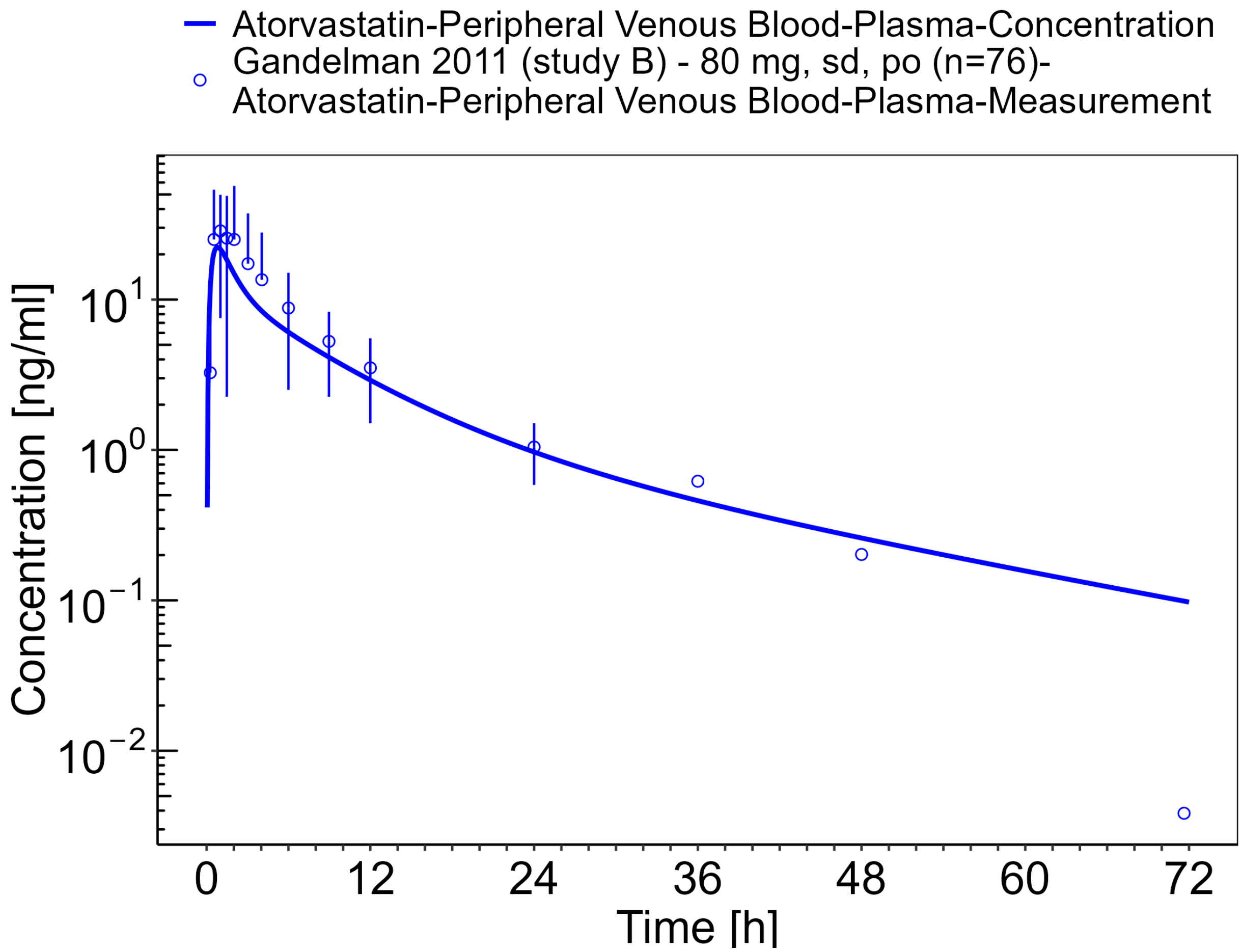

Figure 3-14: Time Profile Analysis

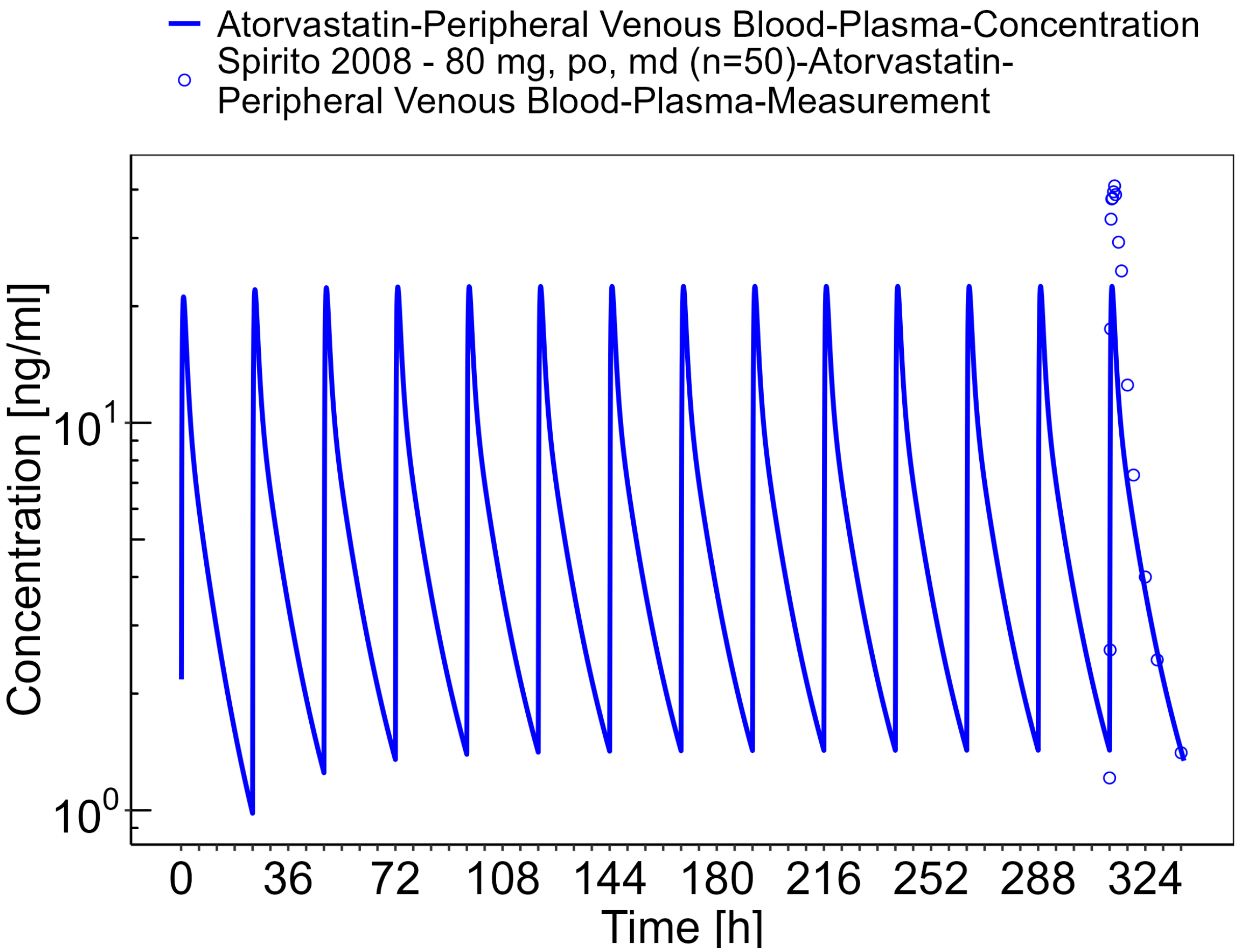

Figure 3-15: Time Profile Analysis

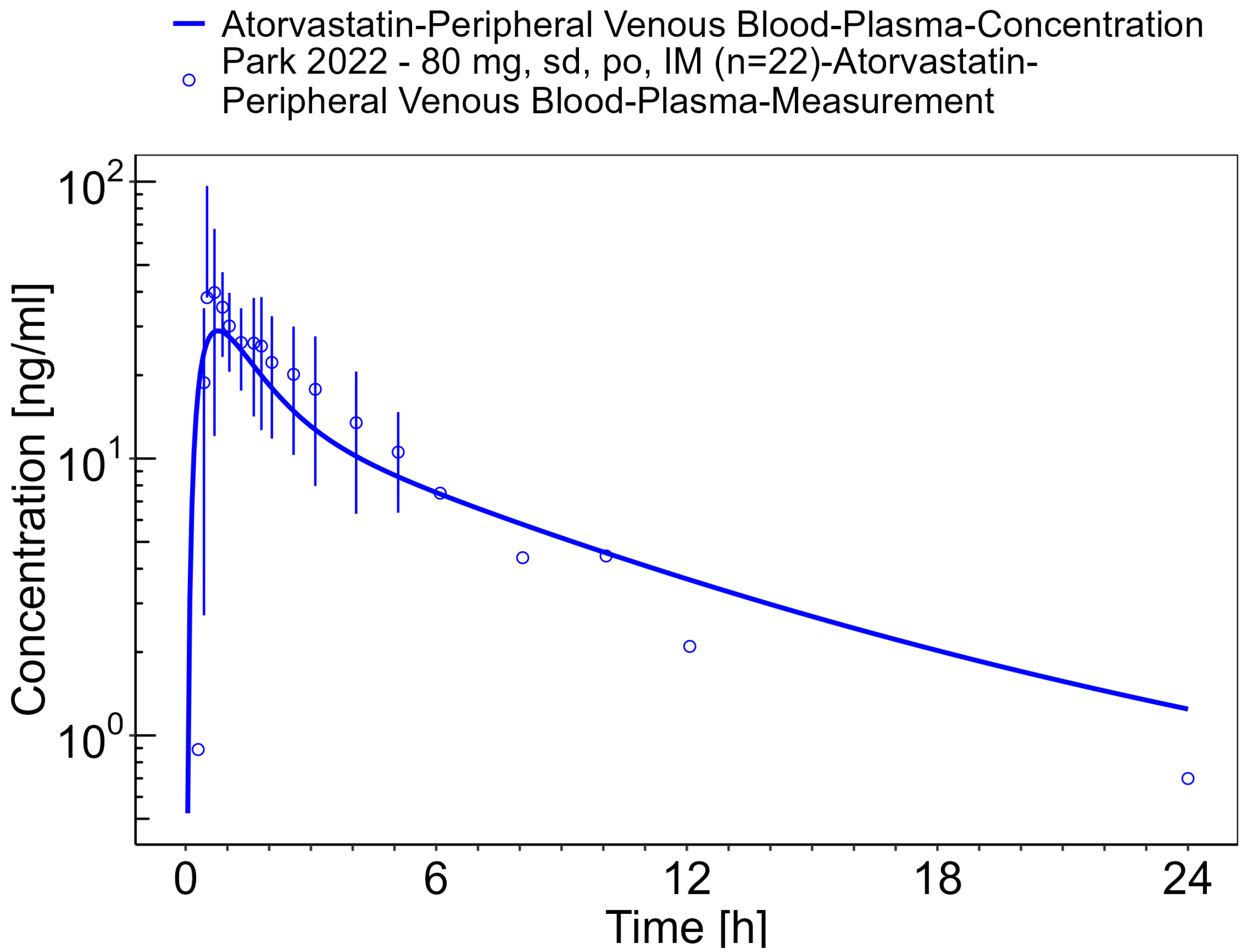

Figure 3-16: Time Profile Analysis

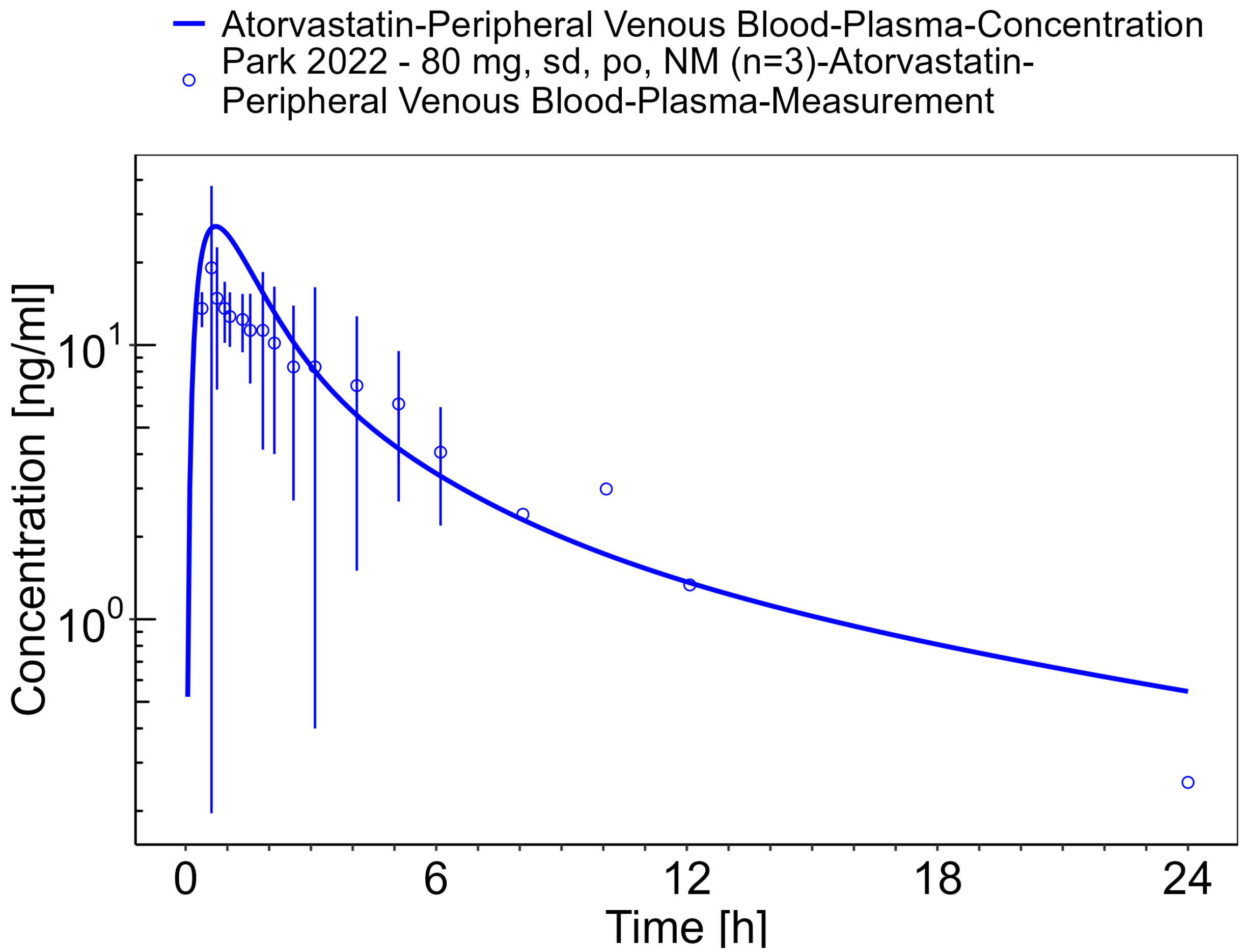

Figure 3-17: Time Profile Analysis

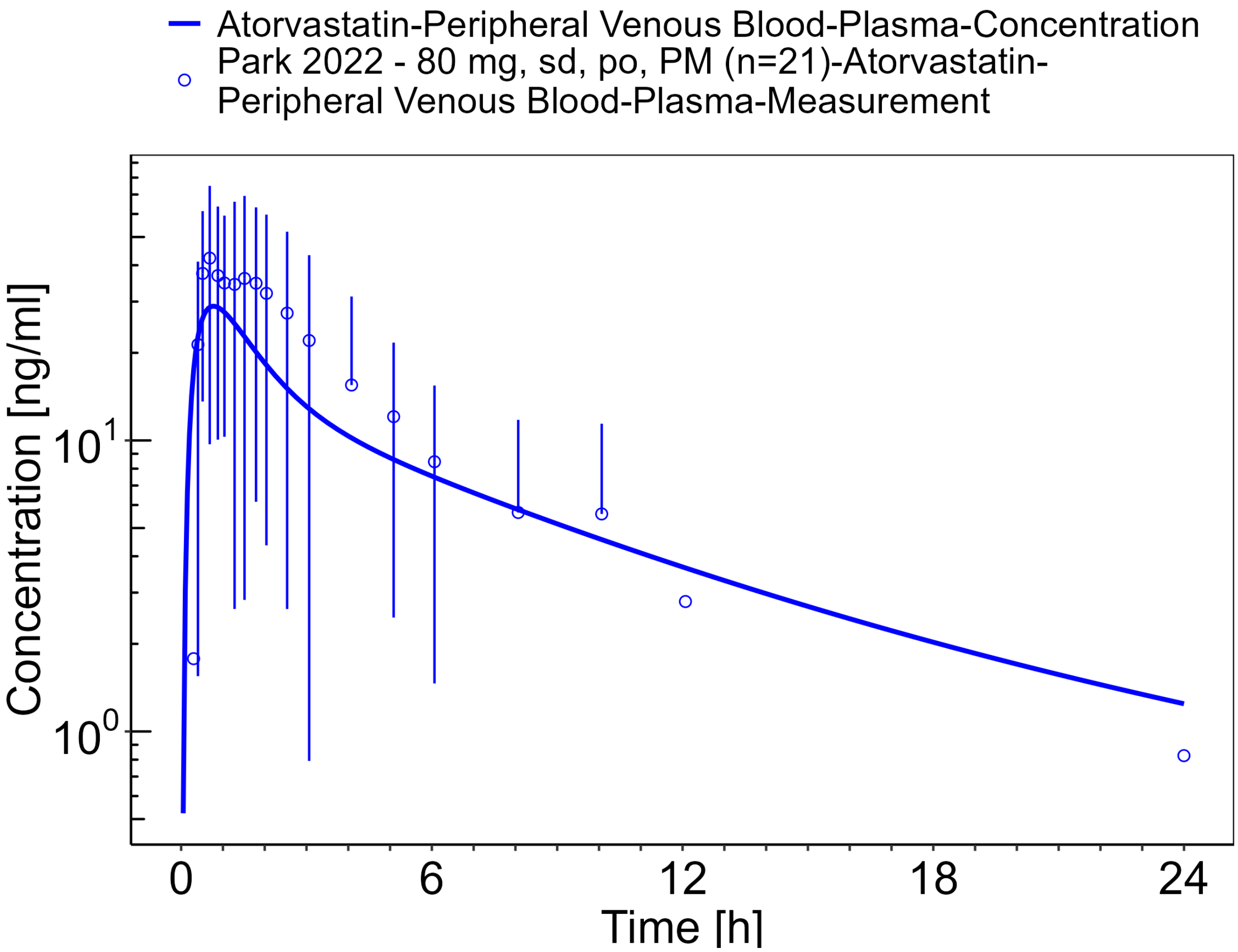

Figure 3-18: Time Profile Analysis

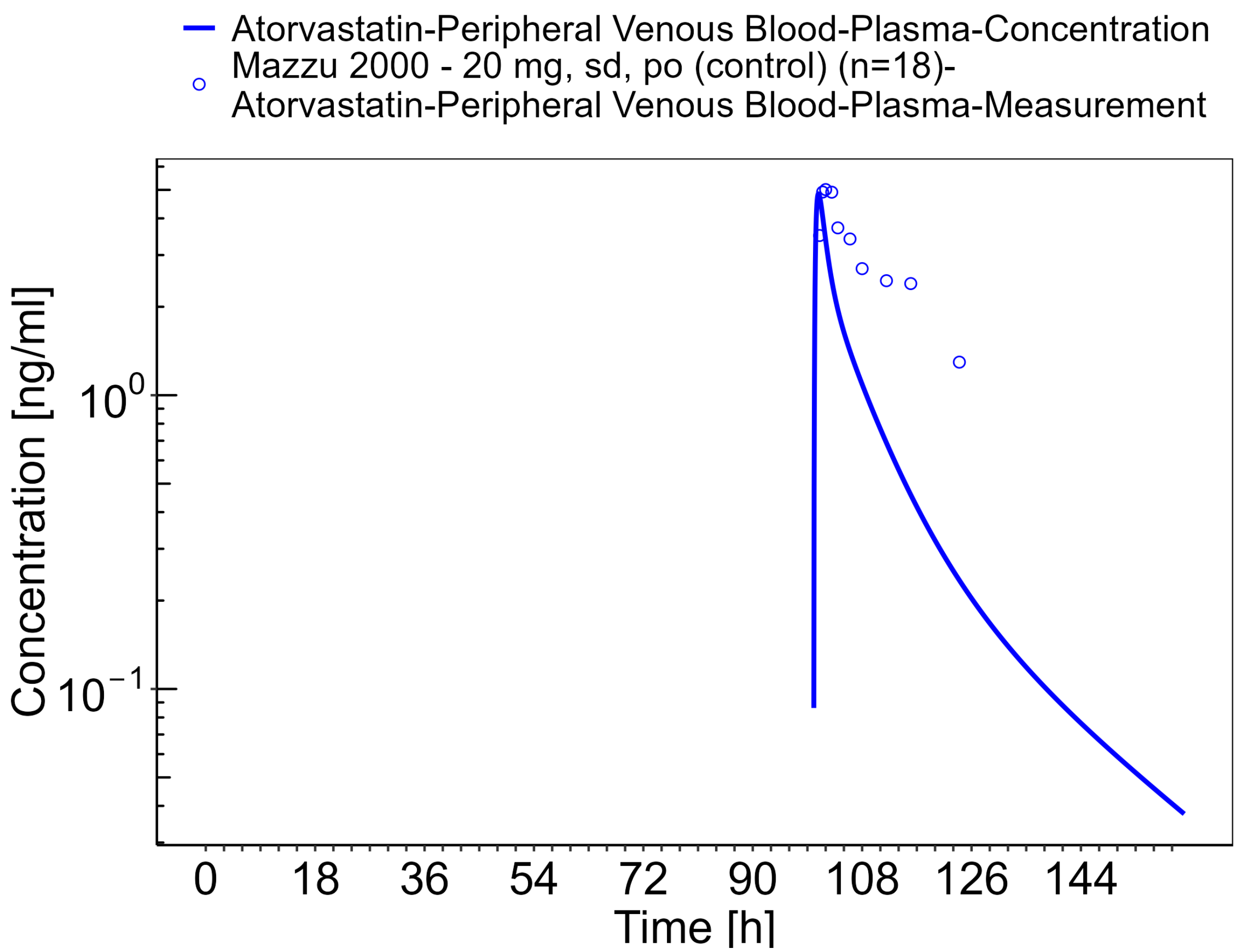

Figure 3-19: Time Profile Analysis

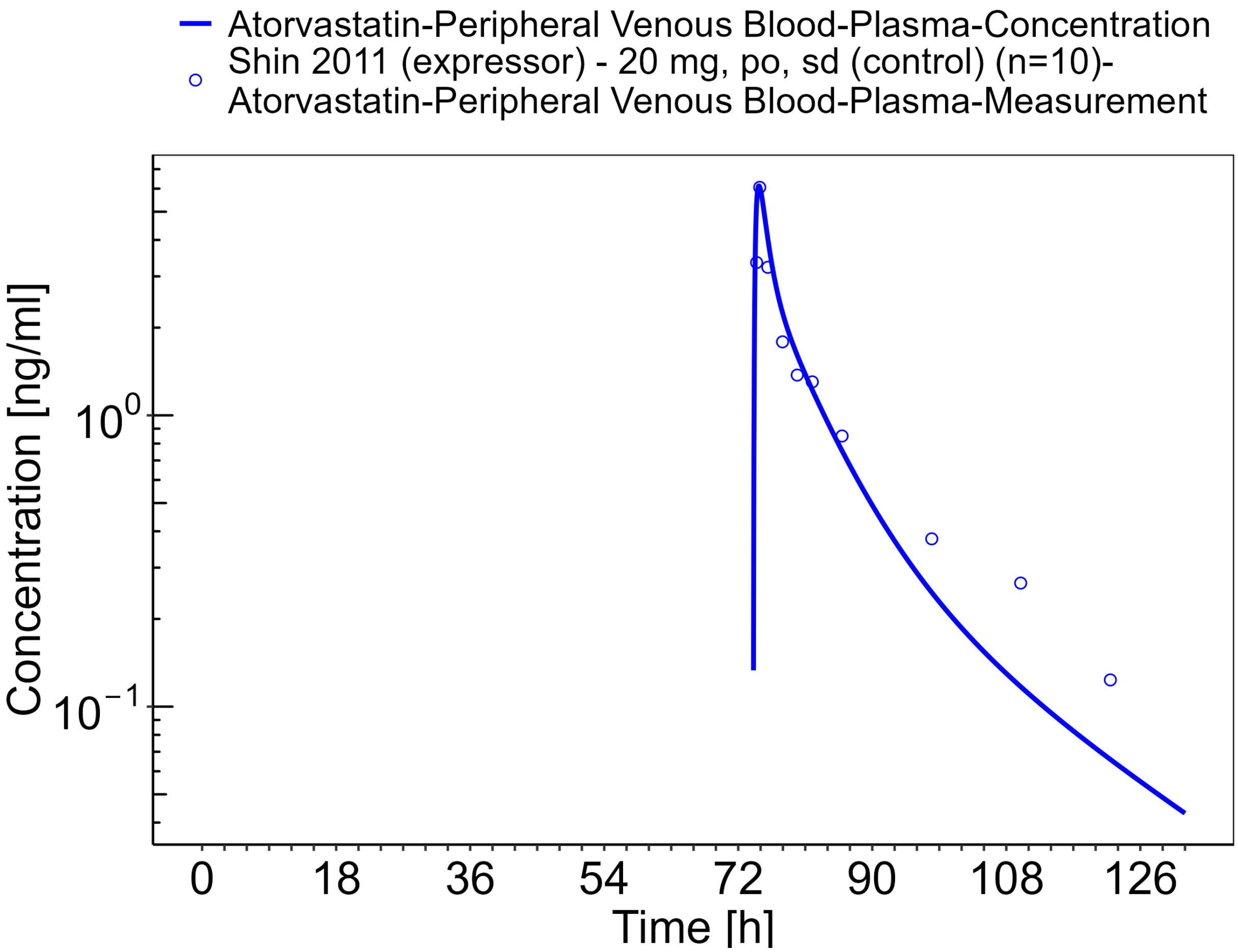

Figure 3-20: Time Profile Analysis

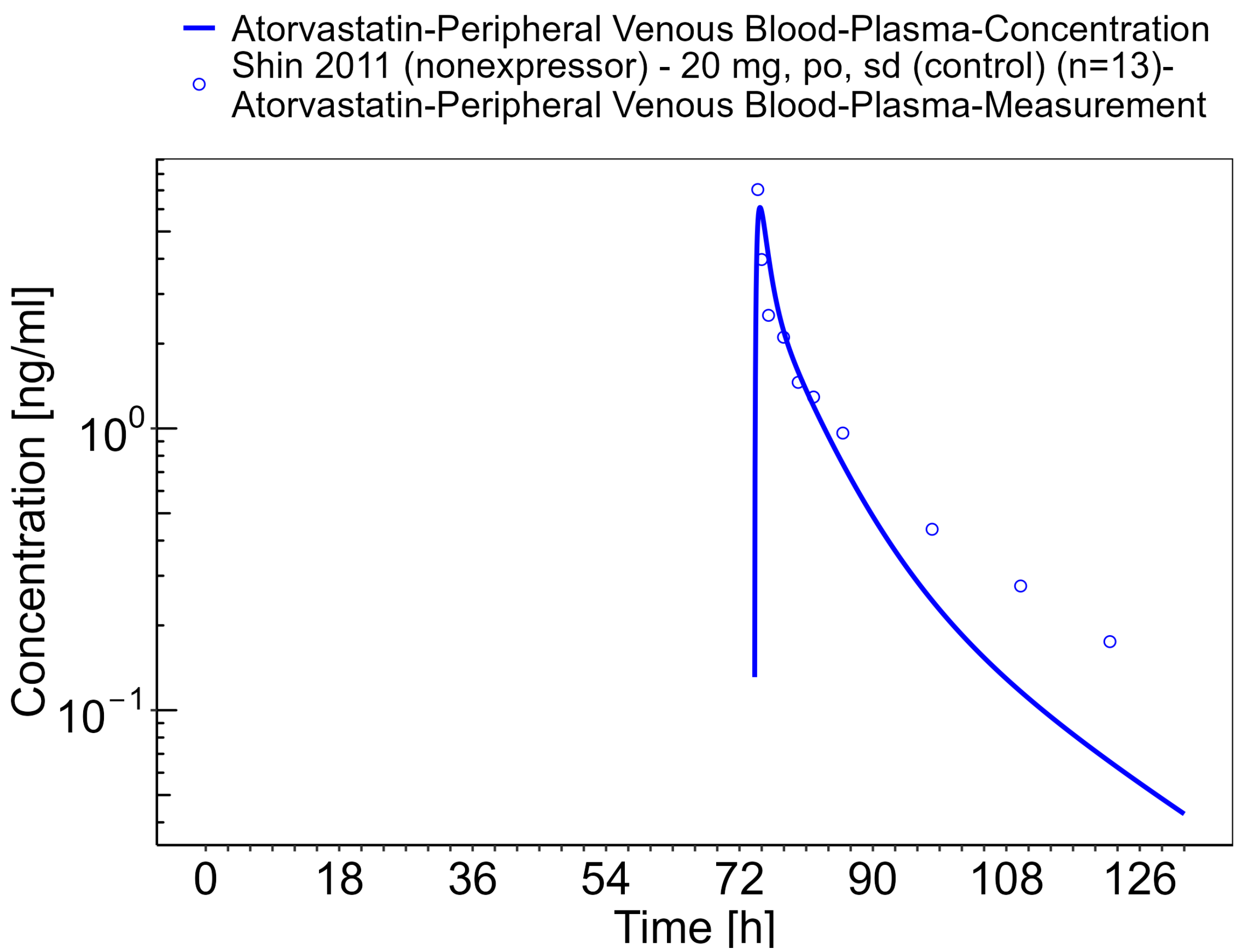

Figure 3-21: Time Profile Analysis

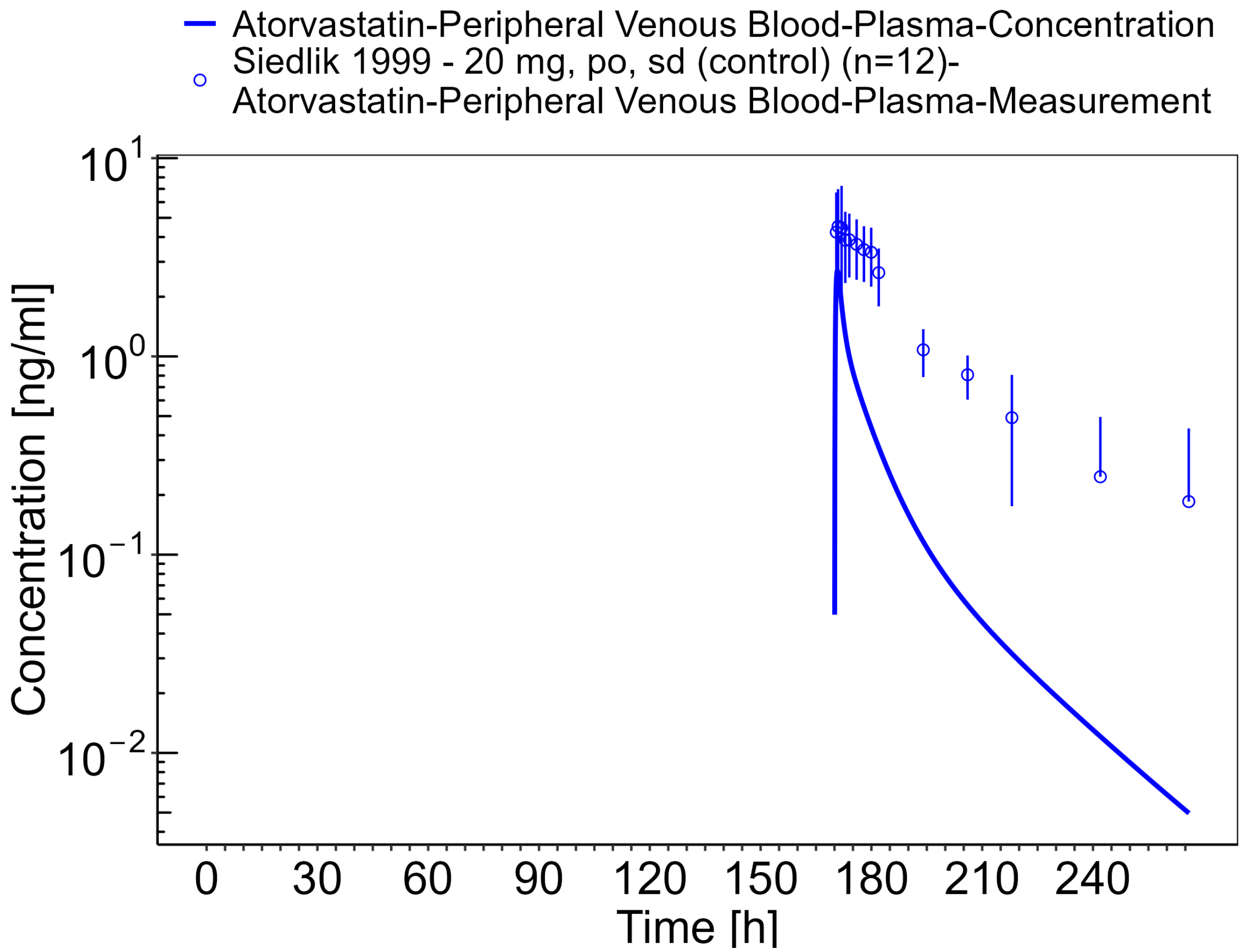

Figure 3-22: Time Profile Analysis

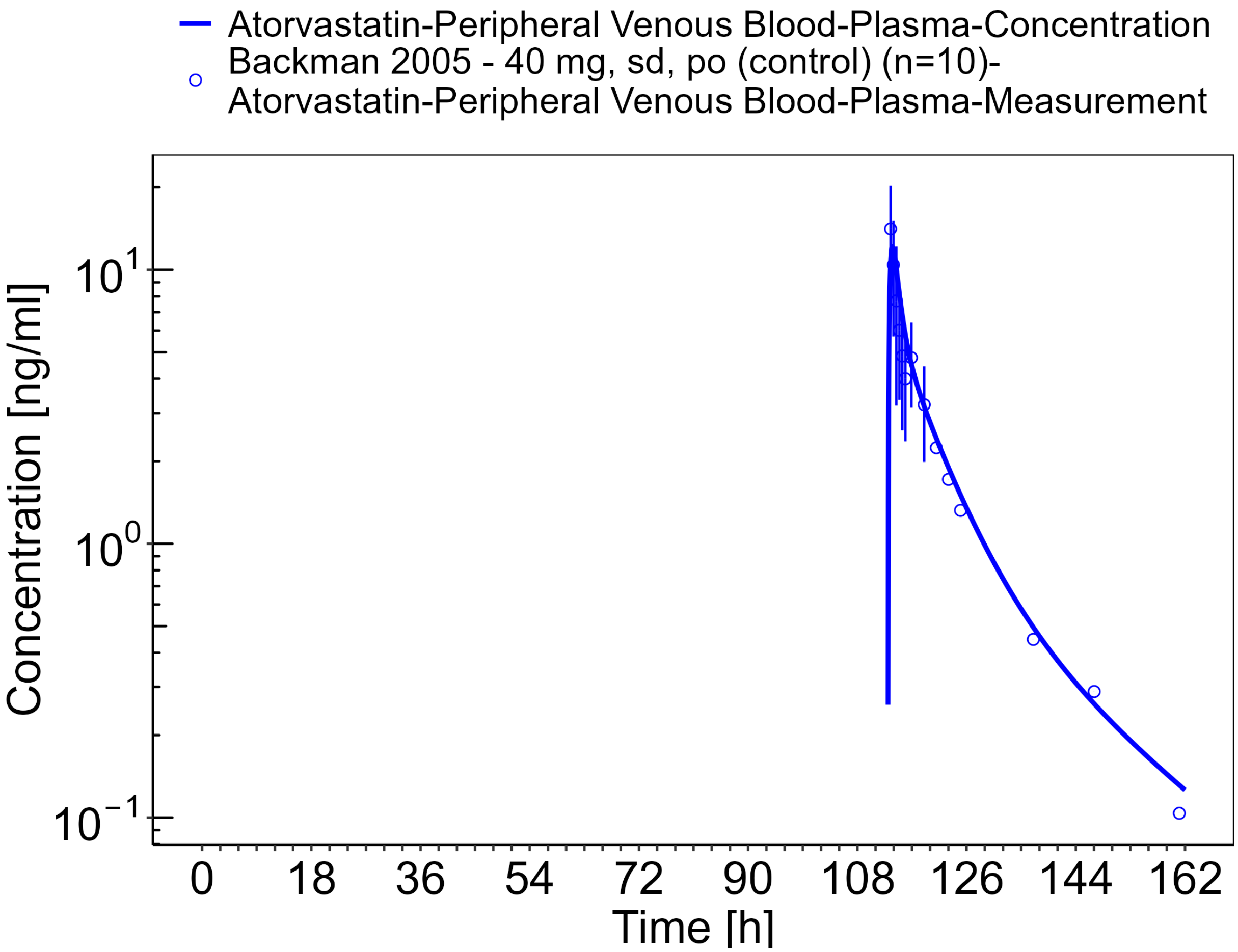

Figure 3-23: Time Profile Analysis

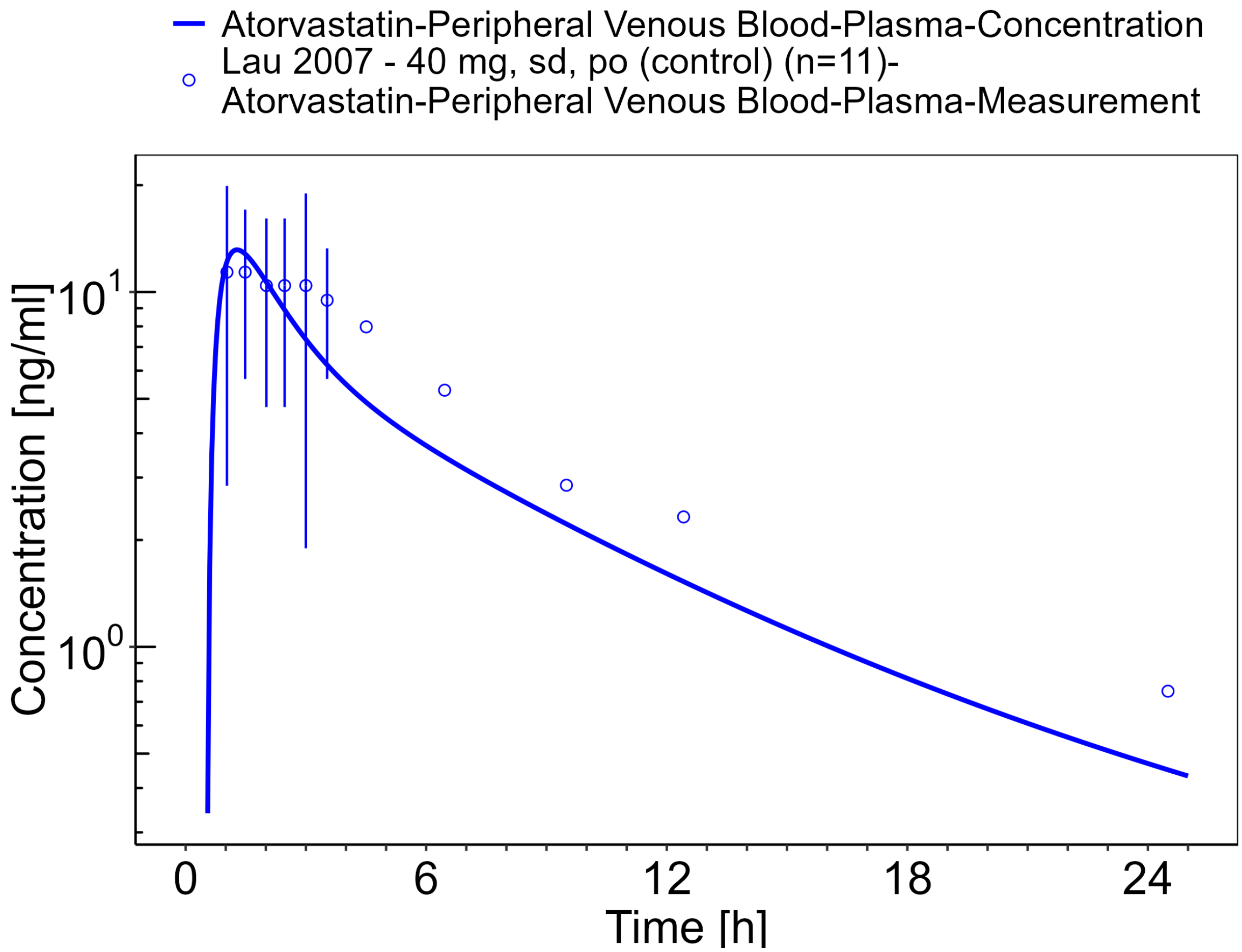

Figure 3-24: Time Profile Analysis

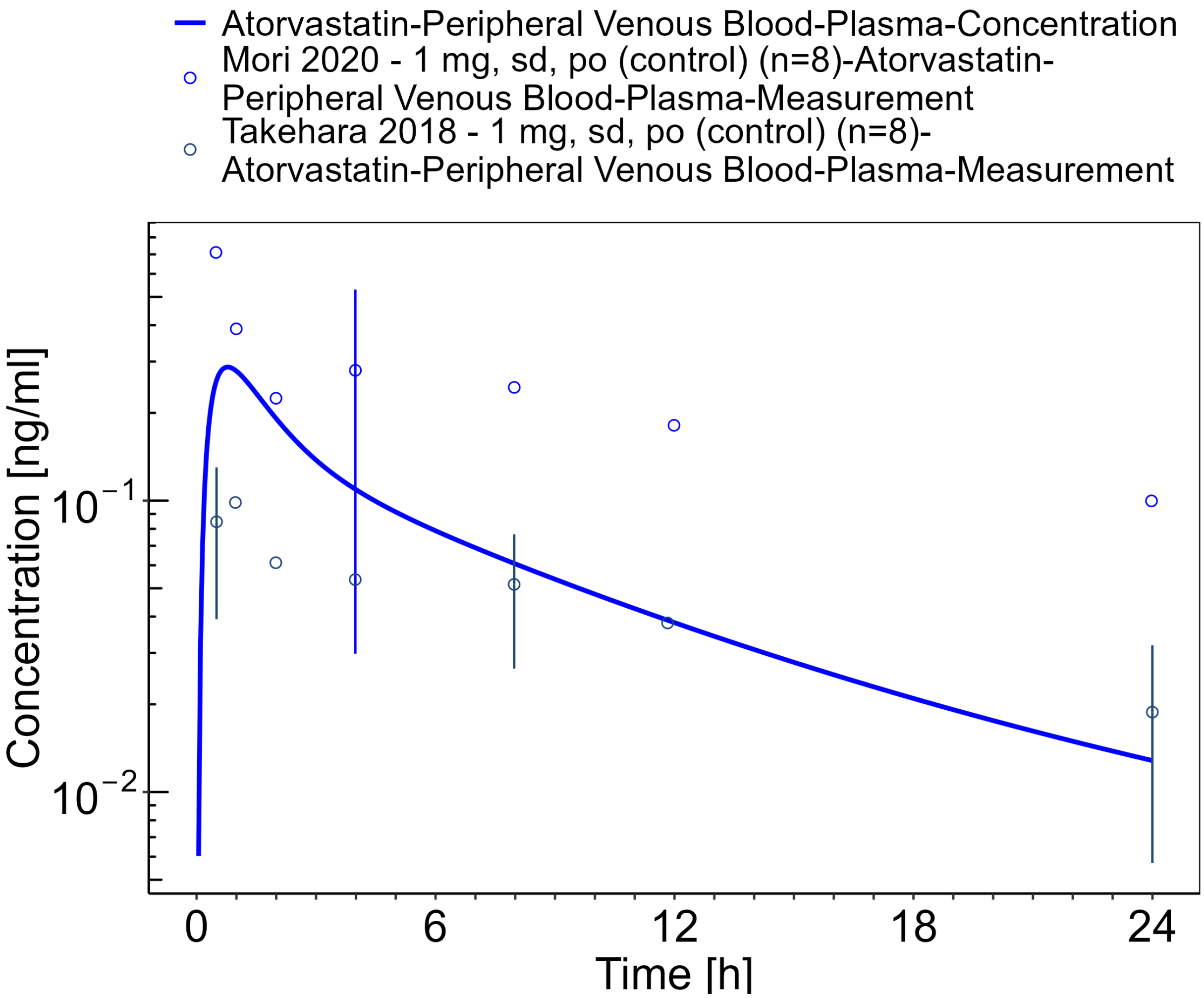

Figure 3-25: Time Profile Analysis

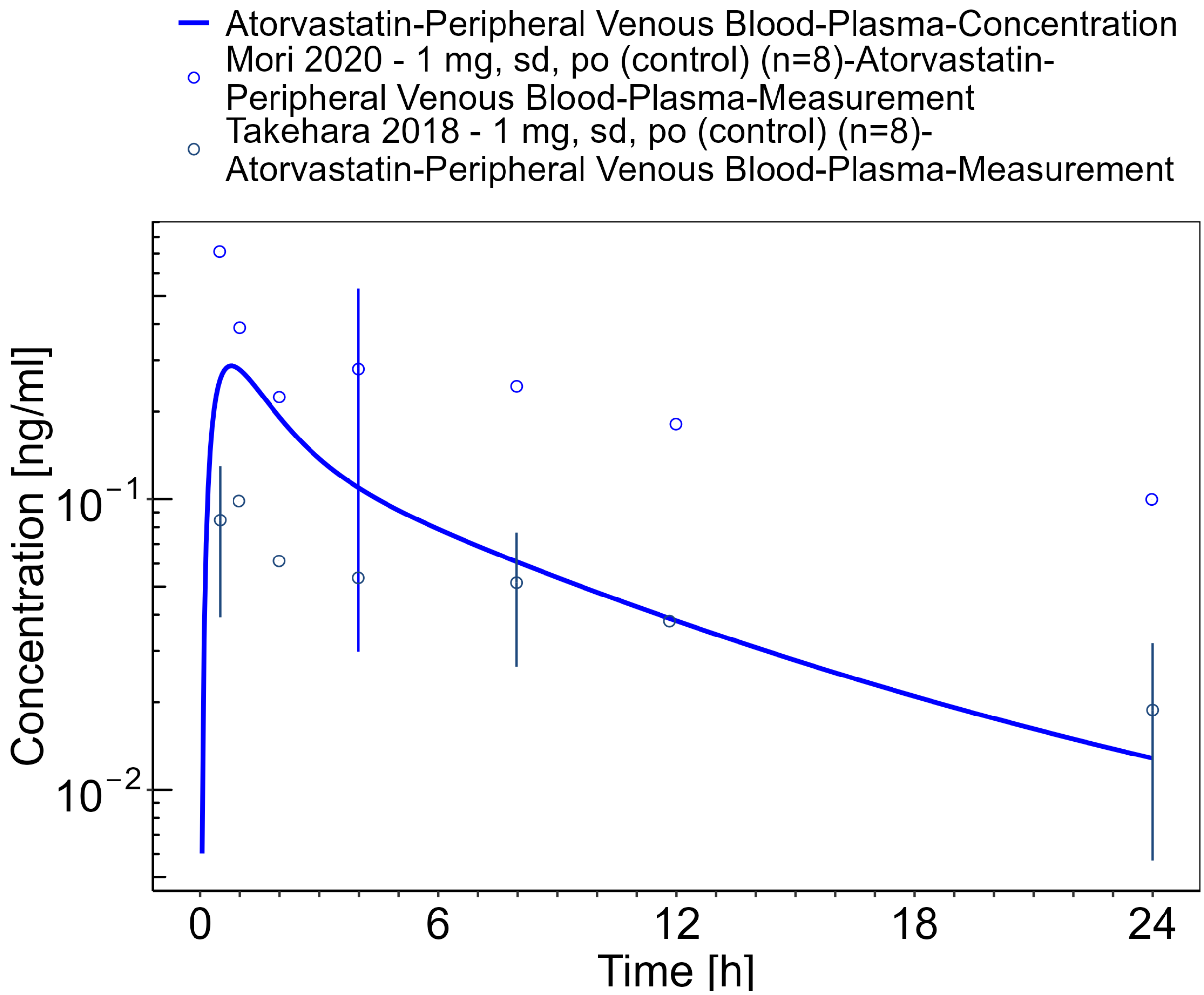

4 Conclusion

A whole-body PBPK model for atorvastatin acid has been successfully developed and verified integrating data of 15 clinical trials.The model is able to describe and predict atorvastatin acid plasma concentration-time profiles after single and multiple oral administrations across the range of 1 to 80 mg in healthy subjects.The final PBPK model comprises metabolism via CYP3A4 and if present, also metabolism via CYP3A5 as well as transport processes via BCRP, OATP1B1/1B3, and P-gp. Moreover, the established PBPK model enriches the openly accessible PBPK model library by a new CYP3A4 and OATP1B1/1B3 substrate.

5 References

Gandelman 2011 Gandelman K, Malhotra B, LaBadie RR, Crownover P, Bergstrom T (2011) Analytes of Interest and Choice of Dose: Two Important Considerations in the Design of Bioequivalence Studies with Atorvastatin. *Journal of Bioequivalence & Bioavailability 3*(4):062–068.

Patiño-Rodríguez 2015 Patiño-Rodríguez O, Martínez-Medina RM, Torres-Roque I, Martínez-Delgado M, Mares-García AS, Escobedo-Moratilla A, Covarrubias-Pinedo A, Arzola-Paniagua A, Herrera-Torres JL, Pérez-Urizar J (2015) Absence of a significant pharmacokinetic interaction between atorvastatin and fenofibrate: a randomized, crossover, study of a fixed-dose formulation in healthy Mexican subjects. *Front Pharmacol. 6*(4).

Bullman 2011 Bullman J, Nicholls A, Van Landingham K, Fleck R, Vuong A, Miller J, Alexander S, Messenheimer J (2011) Effects of lamotrigine and phenytoin on the pharmacokinetics of atorvastatin in healthy volunteers. *Epilepsia 52*(7):1351–8.

Di Spirito 2008 Di Spirito M, Morelli G, Doyle RT, Johnson J, McKenney J (2008) Effect of omega-3-acid ethyl esters on steady-state plasma pharmacokinetics of atorvastatin in healthy adults. *Expert Opin Pharmacother. 9*(17):2939–45.

Mazzu 2000 Mazzu AL, Lasseter KC, Shamblen EC, Agarwal V, Lettieri J, Sundaresen P (2008) Itraconazole alters the pharmacokinetics of atorvastatin to a greater extent than either cerivastatin or pravastatin. *Clin Pharmacol Ther. 68*(4):391–400.

Shin 2011 Shin J, Pauly DF, Pacanowski MA, Langaee T, Frye RF, Johnson JA (2011) Effect of cytochrome P450 3A5 genotype on atorvastatin pharmacokinetics and its interaction with clarithromycin. *Pharmacotherapy 31*(10):942–50.

Siedlik 1999 Siedlik PH, Olson SC, Yang BB, Stern RH. Erythromycin coadministration increases plasma atorvastatin concentrations (1999) *J Clin Pharmacol. 39*(5):501–4.

Backman 2005 Backman JT, Luurila H, Neuvonen M, Neuvonen PJ (2005) Rifampin markedly decreases and gemfibrozil increases the plasma concentrations of atorvastatin and its metabolites. *Clin Pharmacol Ther. 78*(2):154–67.

Whitfield 2010 Whitfield LR, Porcari AR, Alvey C, Abel R, Bullen W, Hartman D (2011) Effect of gemfibrozil and fenofibrate on the pharmacokinetics of atorvastatin. *J Clin Pharmacol. 51*(3):378–88.

Kantola 1998 Kantola T, Kivistö KT, Neuvonen PJ (1998) Effect of itraconazole on the pharmacokinetics of atorvastatin. *Clin Pharmacol Ther. 64*(1):58–65.

Lau 2007 Lau YY, Huang Y, Frassetto L, Benet LZ (2007) effect of OATP1B transporter inhibition on the pharmacokinetics of atorvastatin in healthy volunteers. *Clin Pharmacol Ther. 81*(2):194–204.

Mori 2020 Mori D, Kimoto E, Rago B, Kondo Y, King-Ahmad A, Ramanathan R, Wood LS, Johnson JG, Le VH, Vourvahis M, David Rodrigues A, Muto C, Furihata K, Sugiyama Y, Kusuhara H (2020) Dose-Dependent Inhibition of OATP1B by Rifampicin in Healthy Volunteers: Comprehensive Evaluation of Candidate Biomarkers and OATP1B Probe Drugs. *Clin Pharmacol Ther. 107*(4):1004–1013.

Takehara 2018 Takehara I, Yoshikado T, Ishigame K, Mori D, Furihata KI, Watanabe N, Ando O, Maeda K, Sugiyama Y, Kusuhara H (2018) Comparative Study of the Dose-Dependence of OATP1B Inhibition by Rifampicin Using Probe Drugs and Endogenous Substrates in Healthy Volunteers. *Pharm Res. 35*(7):138.

Teng 2013 Teng R, Mitchell PD, Butler KA (2013) Pharmacokinetic interaction studies of co-administration of ticagrelor and atorvastatin or simvastatin in healthy volunteers. *Eur J Clin Pharmacol. 69*(3):477–87.

Kuepfer 2016 Kuepfer L, Niederalt C, Wendl T, Schlender JF, Willmann S, Lippert J, Block M, Eissing T, Teutonico D. (2016). Applied Concepts in PBPK Modeling: How to Build a PBPK/PD Model. *CPT Pharmacometrics Syst Pharmacol*. Oct;5(10), 516-531.

Willmann 2007 Willmann S, Höhn K, Edginton A, Sevestre M, Solodenko J, Weiss W, Lippert J, Schmitt W. (2007). Development of a physiology-based whole-body population model for assessing the influence of individual variability on the pharmacokinetics of drugs. *J Pharmacokinet Pharmacodyn.* 34(3), 401-431.

PK-Sim Ontogeny Database Version 7.3 Open Systems Pharmacology Suite Community (2018) PK-Sim® Ontogeny Database Documentation version 7.3. https://github.com/Open-Systems-Pharmacology/OSPSuite.Documentation/blob/master/PK-SimOntogenyDatabaseVersion7.3.pdf.

Nishimura 2003 Nishimura M, Yaguti H, Yoshitsugu H, Naito S, Satoh T (2003) Tissue distribution of mRNA expression of human cytochrome P450 isoforms assessed by high-sensitivity real-time reverse transcription PCR. *Journal of the Pharmaceutical Society of Japan 123*(5):369–75.

Kolesnikov 2015 Kolesnikov N, Hastings E, Keays M, Melnichuk O, Tang YA, Williams E, Dylag M, Kurbatova N, Brandizi M, Burdett T, Megy K, Pilicheva E, Rustici G, Tikhonov A, Parkinson H, Petryszak R, Sarkans U, Brazma A (2015) ArrayExpress update—simplifying data submissions. *Nucleic Acids Research 43*(D1):D1113–D1116.

Nishimura 2005 Nishimura M, Naito S (2005) Tissue-specific mRNA expression profiles of human ATP-binding cassette and solute carrier transporter superfamilies. *Drug metabolism and pharmacokinetics 20*(6):452–77.

Zhang 2015 Zhang T (2015) Physiologically based pharmacokinetic modeling of disposition and drug-drug interactions for atorvastatin and its metabolites. *Eur J Pharm Sci. 77*:216–29.

Morse 2019 Morse BL, Alberts JJ, Posada MM, Rehmel J, Kolur A, Tham LS, Loghin C, Hillgren KM, Hall SD, Dickinson GL (2019) Physiologically-Based Pharmacokinetic Modeling of Atorvastatin Incorporating Delayed Gastric Emptying and Acid-to-Lactone Conversion. *CPT Pharmacometrics Syst Pharmacol.* 2019 *8*(9):664–675.

Duan 2017 Duan P, Zhao P, Zhang L (2017) Physiologically Based Pharmacokinetic (PBPK) Modeling of Pitavastatin and Atorvastatin to Predict Drug-Drug Interactions (DDIs). *Eur J Drug Metab Pharmacokinet.* *42*(4):689–705.

Jacobsen 2000 Jacobsen W, Kuhn B, Soldner A, Kirchner G, Sewing KF, Kollman PA, Benet LZ, Christians U (2000) Lactonization is the critical first step in the disposition of the 3-hydroxy-3-methylglutaryl-CoA reductase inhibitor atorvastatin. *Drug Metab Dispos.* *28*(11):1369–78.

Deng 2021 Deng F, Tuomi SK, Neuvonen M, Hirvensalo P, Kulju S, Wenzel C, Oswald S, Filppula AM, Niemi M (2021) Comparative Hepatic and Intestinal Efflux Transport of Statins. *Drug Metab Dispos.* *49*(9):750–759.

Vildhede 2014 Vildhede A, Karlgren M, Svedberg EK, Wisniewski JR, Lai Y, Norén A, Artursson P (2014) Hepatic uptake of atorvastatin: influence of variability in transporter expression on uptake clearance and drug-drug interactions. *Drug Metab Dispos.* *42*(7):1210–8.

Hanke 2018 Hanke N, Frechen S, Moj D, Britz H, Eissing T, Wendl T, Lehr T (2018) PBPK models for CYP3A4 and P-gp DDI prediction: A modeling network of rifampicin, itraconazole, clarithromycin, midazolam, alfentanil, and digoxin. *CPT: Pharmacometrics & Systems Pharmacology* *7*(10):647–659.

Park 2022 Park J-W, Kim J-M, Lee H-Y, Noh J, Kim K-A, Park J-Y (2022) CYP3A5*3 and SLCO1B1 c.521T>C Polymorphisms Influence the Pharmacokinetics of Atorvastatin and 2-Hydroxy Atorvastatin.* Pharmaceutics* *14*(7):1491.

Park 2008 Park JE, Kim KB, Bae SK, Moon BS, Liu KH, Shin JG (2008) Contribution of cytochrome P450 3A4 and 3A5 to the metabolism of atorvastatin. *Xenobiotica* *38*(9):1240-1251.

Pfizer 2019 Pfizer (2019) https://labeling.pfizer.com/ShowLabeling.aspx?id=587. Accessed on 2025-02-11.
