# Supplementary material for: Coproporphyrin I PBPK model in the open systems pharmacology suite enables precise prediction of OATP 1B- mediated drug interactions through calibrated In vivo inhibition constants
Source: Front Pharmacol. 2026 Jul 28;17:1828033. doi: 10.3389/fphar.2026.1828033 (PMC13457544; doi:10.3389/fphar.2026.1828033)
Supplement: Supplementary file 4 [file Supplementaryfile1.docx]

Supplementary Material

# 1 Supplementary Figures

**Supplementary Figure 1.** Flowchart of CP-I model development, model validation, and PBPK-DDI network qualification steps. C_max_, maximum plasma concentration, CP-I, Coproporphyrin I; DDI, Drug-drug interaction; K_i,OATP1B_, *in vivo* inhibition constant for Organic Anion Transporting Polypeptides 1B; PROB, probenecid, RIF, rifampicin.

**Supplementary Figure 2.** Flowchart of model application. C_max_, maximum plasma concentration, CP-I, Coproporphyrin I; DDI, Drug-drug interaction; K_i,OATP1B_, *in vivo* inhibition constant for OATP1B; NCE, new chemical entity; OATP, Organic Anion Transporting Polypeptides;

**Supplementary Figure 3.** Verification of the baseline PBPK CP-I model using observed plasma concentration-time profiles. The solid blue line represents the simulated steady-state plasma concentration of CP-I. Colored circles represent the observed mean from five independent clinical studies (Lai et al., 2016; Shen et al., 2018; Takehara et al., 2018; Mori et al., 2020; Zhang et al., 2020), with error bars indicating the standard deviation (where reported). CP-I, Coproporphyrin I; PBPK, physiologically based pharmacokinetic; SD, standard deviation.

**Supplementary Figure 4.** Observed and predicted plasma concentration-time profiles of CP-I in the absence and presence of rifampicin. (A–D) Blue circles represent observed plasma concentrations of CP-I in the control group. Symbols of other colors (red, green, purple) represent observed concentrations during co-administration with rifampicin (mean and reported variability). The specific dosing regimens were: (A) 600 mg (red) rifampicin (Lai et al., 2016); (B) 300 mg (red) and 600 mg (green) rifampicin (Takehara et al., 2018); (C) 300 mg (red), 600 mg (green), and 150 mg (purple) rifampicin (Mori et al., 2020); and (D) 600 mg (red) rifampicin (Shen et al., 2018). Solid lines represent the PBPK model-predicted profiles for the control (blue) and DDI (red, green, purple) scenarios. CP-I, Coproporphyrin I; DDI, Drug-drug interaction; PBPK, physiologically based pharmacokinetic.

**Supplementary Figure 5.** Observed and predicted plasma concentration-time profiles of CP-I in the absence and presence of cyclosporine A (CsA). (A–C) Light blue circles represent observed plasma concentrations of CP-I in the control group, and dark blue circles represent observed concentrations during co-administration with CsA (mean and reported variability)(Mochizuki et al., 2022). The solid lines represent the PBPK model-predicted profiles for the control (light blue) and DDI (dark blue) scenarios. The dark blue data show the interaction with 20 mg (~1.5 h), 75 mg (~1.5 h), and and 75 mg (~3 h) CsA, respectively. CP-I, Coproporphyrin I; CsA, Cyclosporine A; DDI, Drug-drug interaction; PBPK, physiologically based pharmacokinetic; SD, standard deviation.

**Supplementary Figure 6.** Observed and predicted plasma concentration-time profiles of pitavastatin in the absence and presence of cyclosporine A (CsA). (A–C) Light green circles represent observed plasma concentrations of pitavastatin in the control group, and dark green circles represent observed concentrations during co-administration with CsA (mean and reported variability) (Mochizuki et al., 2022). The solid lines represent the PBPK model-predicted profiles for the control (light green) and DDI (dark green) scenarios. The dark green data show the interaction with 20 mg (~1.5 h), 75 mg (~1.5 h), and and 75 mg (~3 h) CsA, respectively. CsA, Cyclosporine A; DDI, Drug-drug interaction; PBPK, physiologically based pharmacokinetic.

**Supplementary Figure 7.** Observed and predicted plasma concentration-time profiles of rosuvastatin in the absence and presence of cyclosporine A (CsA). (A–C) Yellow circles represent observed plasma concentrations of CP-I in the control group, and orange circles represent observed concentrations during co-administration with CsA (mean and reported variability) (Mochizuki et al., 2022). The solid lines represent the PBPK model-predicted profiles for the control (yellow) and DDI (orange) scenarios. The orange data show the interaction with 20 mg (~1.5 h), 75 mg (~1.5 h), and and 75 mg (~3 h) CsA, respectively. CsA, Cyclosporine A; DDI, Drug-drug interaction; PBPK, physiologically based pharmacokinetic.

# 2 Supplementary Tables

**Supplementary Table 1.** Clinical study data used for CP-I PBPK model development

| Route | Rifampicin dose [mg] | n | Females [%] | Age range [years] | Race or Ethnicity | Body weight [kg] | BMI [kg/m^2^] | References |
| --- | --- | --- | --- | --- | --- | --- | --- | --- |
| p.o. (s.d.) | 0, 600 | 12 | 0 | 18-45 | Indian | ≥50 | 18.5-25.0 | Lai et al. (2016) |
| p.o.  (s.d.) | 600 | 12 | -* | -* | -* | -* | -* | Kunze et al. (2018) |
| p.o. (s.d.) | 600 | 8 | 0 | 26-36 | Japanese | 53.5-71.7 | 19.1-24.8 | Mori et al. (2020) |
| p.o.  (s.d.) | 600 | 16 | 0 | 26-47 | Hispanic, White, and Black | -* | 21.6-31.2 | Shen et al. (2018) |
| p.o.  (s.d.) | 0 | 8 | 0 | 26-36 | Japanese | 53.5-71.7 | 19.1-24.8 | Takehara et al. (2018) |
| p.o.  (s.d.) | 0, 1000 | 14 | 0 | -* | Indian | -* | -* | Zhang et al. (2020) |

*Data not reported in the original source. All included subjects are healthy volunteers. CP-I, Coproporphyrin I; i.v., intravenous; n, number of subjects; p.o, oral administration; PBPK, physiologically based pharmacokinetic; s.d., single dose.

**Supplementary Table 2.** Overview of OSP PBPK models and qualification reports used in this analysis.

| Compound | OSP Suite Version | Repository / Qualification report URL |
| --- | --- | --- |
| Object |  |  |
| Atorvastatin | 11.2 | <https://github.com/Open-Systems-Pharmacology/Atorvastatin-Model> |
| Pitavastatin | 11.2 | <https://github.com/Open-Systems-Pharmacology/Pitavastatin-Model> |
| Rosuvastatin | 11.2 | [https://github.com/Open-Systems-Pharmacology/Rosuvastatin-Model](https://www.google.com/search?q=https://github.com/Open-Systems-Pharmacology/Rosuvastatin-Model) |
| Coproporphyrin-I | 11.2 | https://github.com/Open-Systems-Pharmacology/Coproporphyrin-I |
| Precipitants |  |  |
| Rifampicin | 11.2 | <https://github.com/Open-Systems-Pharmacology/Rifampicin-Model> |
| Probenecid | 11.2 | <https://github.com/Open-Systems-Pharmacology/Probenecid-Model> |
| Cyclosporine A | 9.1 | <https://github.com/Open-Systems-Pharmacology/Cyclosporine-Model> |

**Supplementary Table 3.** Summary table of clinical DDI studies used for PBPK-DDI network qualification.

| Precipitant | Object | Precipitant Dosing | Object Dosing | Clinical Study References |
| --- | --- | --- | --- | --- |
| Rifampicin | Coproporphyrin I | 150-600 mg oral | Endogenous | Lai et al. (2016); Kunze et al. (2018); Shen et al. (2018); Takehara et al. (2018); Mori et al. (2020) |
| Rifampicin | Atorvastatin | 150-600 mg oral/i.v., single/multiple | 1-40 mg | Backman et al. (2005); Lau et al. (2007); Takehara et al. (2018); Mori et al. (2020) |
| Rifampicin | Pitavastatin | 600 mg oral/i.v., single | 0.2-4 mg | Chen et al. (2013) ; Prueksaritanont et al. (2014); Takehara et al. (2018); Mori et al. (2020) |
| Rifampicin | Rosuvastatin | 600 mg oral/i.v., single | 0.5-20 mg | Prueksaritanont et al. (2014, 2017); Wu et al. (2017); Wiebe et al. (2020) |
| Probenecid | Coproporphyrin I | 1000 mg oral | Endogenous | Zhang et al. (2020) |
| Probenecid | Rosuvastatin | 1000 mg oral, multiple | 10 mg | Wiebe et al. (2020) |

DDI, drug-drug interaction; i.v., intravenous; PBPK, physiologically based pharmacokinetic.

**Supplementary Table 4**. Literature sources of clinical data for CP-I, rosuvastatin, atorvastatin, and pitavastatin used for the PBPK modeling of DDIs with rifampicin and probenecid.

| **Source** | **Route** | **Dose [mg] / Schedule*** | **Pop.** | **N** | **Purpose^+^** |
| --- | --- | --- | --- | --- | --- |
| *Coproporphyrin I – rifampicin* | | | | |  |
| Lai 2016 | p.o. | 600 mg rifampicin | HV | 12 | Development |
| Kunze 2018 | p.o. | 600 mg rifampicin | HV | 12 | Development |
| Mori 2020 | p.o. | 150 mg rifampicin | HV | 8 | Qualification |
| Mori 2020 | p.o. | 300 mg rifampicin | HV | 8 | Qualification |
| Mori 2020 | p.o. | 600 mg rifampicin | HV | 8 | Development |
| Shen 2018 | p.o. | 600 mg rifampicin | HV | 16 | Development |
| Takehara 2018 | p.o. | 300 mg rifampicin | HV | 8 | Qualification |
| Takehara 2018 | p.o. | 600 mg rifampicin | HV | 8 | Qualificationt |
| *Coproporphyrin I – probenecid* | | | | |  |
| Zhang 2020 | p.o. | 1000 mg probenecid | HV | 14 | Development |
| *Rosuvastatin – rifampicin* | | | | |  |
| Prueksaritanont 2014 | i.v./p.o. | 600 mg i.v. rifampicin / 5 mg p.o. rosuvastatin | HV | 8 | Qualification |
| Prueksaritanont 2014 | p.o. | 600 mg p.o. rifampicin / 5 mg p.o. rosuvastatin | HV | 8 | Qualification |
| Prueksaritanont 2017 | p.o. | 600 mg p.o rifampicin / 0.5 mg p.o. rosuvastatin | HV | 8 | Qualification |
| Wiebe 2020 | p.o. | 600 mg p.o rifampicin / 10mg p.o. rosuvastatin | HV | 11 | Qualification |
| Wu 2017 | i.v./p.o. | 600 mg i.v. rifampicin / 20 mg p.o. rosuvastatin | HV | 7 | Qualification |
| *Rosuvastatin – probenecid* |  |  |  |  |  |
| Wiebe 2020 | p.o. | 1000 mg p.o probenecid b.i.d / 10 mg p.o. rosuvastatin | HV | 13 | Qualification |
| *Atorvastatin – rifampicin* | | | | |  |
| Lau 2007 | i.v./p.o. | 600 mg i.v. rifampicin / 40 mg p.o. atorvastatin | HV | 11 | Qualification |
| Backmann 2005 | p.o. | 600 mg MD rifampicin / 40 mg atorvastatin | HV | 10 | Qualification |
| Mori 2020 | p.o. | 150 mg rifampicin / 1 mg atorvastatin | HV | 8 | Qualification |
| Mori 2020 | p.o. | 300 mg rifampicin / 1 mg atorvastatin | HV | 8 | Qualification |
| Mori 2020 | p.o. | 600 mg rifampicin / 1 mg atorvastatin | HV | 8 | Qualification |
| Takehara 2018 | p.o. | 300 mg rifampicin / 1 mg atorvastatin | HV | 8 | Qualification |
| Takehara 2018 | p.o. | 600 mg rifampicin / 1 mg atorvastatin | HV | 8 | Qualification |
| *Pitavastatin – rifampicin* | | | | |  |
| Chen 2013 | p.o. | 600 mg rifampicin / 4 mg pitavastatin | HV | 12 | Qualification |
| Mori 2020 | p.o. | 150 mg rifampicin / 0.2 mg pitavastatin | HV | 8 | Qualification |
| Mori 2020 | p.o. | 300 mg rifampicin / 0.2 mg pitavastatin | HV | 8 | Qualification |
| Mori 2020 | p.o. | 600 mg rifampicin / 0.2 mg pitavastatin | HV | 8 | Qualification |
| Takehara 2018 | p.o. | 300 mg rifampicin / 0.2 mg pitavastatin | HV | 8 | Qualification |
| Takehara 2018 | p.o. | 600 mg rifampicin / 0.2 mg pitavastatin | HV | 8 | Qualification |
| Prueksaritanont 2014 | i.v./p.o. | 600 mg i.v. rifampicin / 1 mg p.o. pitavastatin | HV | 8 | Qualification |
| Prueksaritanont 2014 | p.o. | 600 mg p.o. rifampicin / 1 mg p.o. pitavastatin | HV | 8 | Qualification |

*Single dose unless stated otherwise. ^+^Clinical data sources are categorized by their purpose in the modeling workflow, stating if data was used for development or network qualification. Lai 2016 refers to Lai et al. (2016); Kunze 2018 refers to Kunze et al. (2018); Shen 2018 refers to Shen et al. (2018); Takehara 2018 refers to Takehara et al. (2018); Mori 2020 refers to Mori et al. (2020); Backman 2005 refers to Backman et al. (2005); Lau 2007 refers to Lau et al. (2007); Chen 2013 refers to Chen et al. (2013) ; Prueksaritanont 2014 refers to Prueksaritanont et al. (2014); Prueksaritanont 2017 refers to Prueksaritanont et al. (2017); Wu 2017 refers to Wu et al. (2017); Wiebe 2020 refers to Wiebe et al. (2020); Zhang 2020 refers to Zhang et al. (2020). CP-I, Coproporphyrin I; DDI, drug-drug interaction; HV, healthy volunteers; i.v., intravenous; MD, multiple doses; N, number of subjects; p.o, oral administration; PBPK, physiologically based pharmacokinetic; Pop., population.

**Supplementary Table 5**. Summary of predictive accuracy for the qualification of the OATP1B1/3 DDI network.

|  | AUC_last_ | | C_max_ | |
| --- | --- | --- | --- | --- |
|  | Count | Percentage [%]* | Count | Percentage [%]* |
| Total studies | 30 | - | 30 | - |
| Within Guest et al. limits ^+^ | 25 | 83.3 | 28 | 93.3 |
| Within 2-fold | 27 | 90.0 | 29 | 96.7 |

*Values represent the percentage of studies within the specified fold-error limits (calculated as the count within limits as a percentage of the total). ^+^Limits of the predictive measure proposed by Guest et al. (2011) with 1.25-fold variability. AUC_last_, area under the plasma concentration-time curve from time zero to the last measurable concentration; C_max_, maximum plasma concentration.

**Supplementary Table 6**. Comparison of *in vivo* K_i,OATP1B_ for rifampicin fitted separately for each study vs fitted together.

| Study | Ki [µM] | 95 % CI [µM] |
| --- | --- | --- |
| fitted together | 0.17 | 0.12-0.22 |
| Lai 2016 | 0.08 | 0.04 - 0.12 |
| Kunze 2018 | 0.36 | 0.18 - 0.54+-0.176 |
| Shen 2018 | 0.18 | 0.17 -0.19 |
| Takahara 2018 | 0.16 | 0.068 - 0.25 |

# 3 References

Backman, J., Luurila, H., Neuvonen, M., and Neuvonen, P. (2005). Rifampin markedly decreases and gemfibrozil increases the plasma concentrations of atorvastatin and its metabolites. *Clin. Pharmacol. Ther.* 78, 154–167. doi: 10.1016/j.clpt.2005.04.007

Chen, Y., Zhang, W., Huang, W., Tan, Z., Wang, Y., Huang, X., et al. (2013). Effect of a single-dose rifampin on the pharmacokinetics of pitavastatin in healthy volunteers. *Eur. J. Clin. Pharmacol.* 69, 1933–1938. doi: 10.1007/s00228-013-1554-0

Guest, E. J., Aarons, L., Houston, J. B., Rostami-Hodjegan, A., and Galetin, A. (2011). Critique of the Two-Fold Measure of Prediction Success for Ratios: Application for the Assessment of Drug-Drug Interactions. *Drug Metab. Dispos.* 39, 170–173. doi: 10.1124/dmd.110.036103

Kunze, A., Ediage, E. N., Dillen, L., Monshouwer, M., and Snoeys, J. (2018). Clinical Investigation of Coproporphyrins as Sensitive Biomarkers to Predict Mild to Strong OATP1B-Mediated Drug–Drug Interactions. *Clin. Pharmacokinet.* 57, 1559–1570. doi: 10.1007/s40262-018-0648-3

Lai, Y., Mandlekar, S., Shen, H., Holenarsipur, V. K., Langish, R., Rajanna, P., et al. (2016). Coproporphyrins in Plasma and Urine Can Be Appropriate Clinical Biomarkers to Recapitulate Drug-Drug Interactions Mediated by Organic Anion Transporting Polypeptide Inhibition. *J. Pharmacol. Exp. Ther.* 358, 397–404. doi: 10.1124/jpet.116.234914

Lau, Y. Y., Huang, Y., Frassetto, L., and Benet, L. Z. (2007). Effect of OATP1B Transporter Inhibition on the Pharmacokinetics of Atorvastatin in Healthy Volunteers. *Clin. Pharmacol. Ther.* 81, 194–204. doi: 10.1038/sj.clpt.6100038

Mochizuki, T., Aoki, Y., Yoshikado, T., Yoshida, K., Lai, Y., Hirabayashi, H., et al. (2022). Physiologically‐based pharmacokinetic model‐based translation of OATP1B ‐mediated drug–drug interactions from coproporphyrin I to probe drugs. *Clin. Transl. Sci.* 15, 1519–1531. doi: 10.1111/cts.13272

Mori, D., Ishida, H., Mizuno, T., Kusumoto, S., Kondo, Y., Izumi, S., et al. (2020). Alteration in the Plasma Concentrations of Endogenous Organic Anion–Transporting Polypeptide 1B Biomarkers in Patients with Non–Small Cell Lung Cancer Treated with Paclitaxel. *Drug Metab. Dispos.* 48, 387–394. doi: 10.1124/dmd.119.089474

Prueksaritanont, T., Chu, X., Evers, R., Klopfer, S. O., Caro, L., Kothare, P. A., et al. (2014). Pitavastatin is a more sensitive and selective organic anion‐transporting polypeptide 1B clinical probe than rosuvastatin. *Br. J. Clin. Pharmacol.* 78, 587–598. doi: 10.1111/bcp.12377

Prueksaritanont, T., Tatosian, D., Chu, X., Railkar, R., Evers, R., Chavez‐Eng, C., et al. (2017). Validation of a microdose probe drug cocktail for clinical drug interaction assessments for drug transporters and CYP3A. *Clin. Pharmacol. Ther.* 101, 519–530. doi: 10.1002/cpt.525

Shen, H., Christopher, L., Lai, Y., Gong, J., Kandoussi, H., Garonzik, S., et al. (2018). Further Studies to Support the Use of Coproporphyrin I and III as Novel Clinical Biomarkers for Evaluating the Potential for Organic Anion Transporting Polypeptide 1B1 and OATP1B3 Inhibition. *Drug Metab. Dispos.* 46, 1075–1082. doi: 10.1124/dmd.118.081125

Takehara, I., Yoshikado, T., Ishigame, K., Mori, D., Furihata, K., Watanabe, N., et al. (2018). Comparative Study of the Dose-Dependence of OATP1B Inhibition by Rifampicin Using Probe Drugs and Endogenous Substrates in Healthy Volunteers. *Pharm. Res.* 35, 138. doi: 10.1007/s11095-018-2416-3

Wiebe, S. T., Giessmann, T., Hohl, K., Schmidt-Gerets, S., Hauel, E., Jambrecina, A., et al. (2020). Validation of a Drug Transporter Probe Cocktail Using the Prototypical Inhibitors Rifampin, Probenecid, Verapamil, and Cimetidine. *Clin. Pharmacokinet.* 59, 1627–1639. doi: 10.1007/s40262-020-00907-w

Wu, H.-F., Hristeva, N., Chang, J., Liang, X., Li, R., Frassetto, L., et al. (2017). Rosuvastatin Pharmacokinetics in Asian and White Subjects Wild Type for Both OATP1B1 and BCRP Under Control and Inhibited Conditions. *J. Pharm. Sci.* 106, 2751–2757. doi: 10.1016/j.xphs.2017.03.027

Zhang, Y., Holenarsipur, V. K., Kandoussi, H., Zeng, J., Mariappan, T. T., Sinz, M., et al. (2020). Detection of Weak Organic Anion–Transporting Polypeptide 1B Inhibition by Probenecid with Plasma-Based Coproporphyrin in Humans. *Drug Metab. Dispos.* 48, 841–848. doi: 10.1124/dmd.120.000076
